# Investigation of Radiolabeled KISS1R Ligands as Promising Tools for Diagnosis and Treatment of Triple-Negative Breast Cancer

**DOI:** 10.1021/acs.molpharmaceut.5c01853

**Published:** 2026-03-03

**Authors:** Harun Taş, Martin Schäfer, Aneeba Shuja-Uddin, Ulrike Bauder-Wüst, Luciana Kovacs Dos Santos, Lisa Bartnitzky, Felix Oden, Magdalena Platzk, Tim König, Patrick Leopold Rüther, Elisabeth Pook, Kateřina Dvořáková Bendová, Zbyněk Nový, Miloš Petřík, Urs B. Hagemann, Martina Benešová-Schäfer

**Affiliations:** 1 Research Group Translational Radiotheranostics, German Cancer Research Center (DKFZ), Im Neuenheimer Feld 280, Heidelberg 69120, Germany; 2 Service Unit for Radiopharmaceuticals and Preclinical Studies, German Cancer Research Center (DKFZ), Im Neuenheimer Feld 280, Heidelberg 69120, Germany; 3 1569 Bayer AG, Berlin 13342, Germany; 4 1569 Bayer AG, Wuppertal 42113, Germany; 5 Institute of Molecular and Translational Medicine, Faculty of Medicine and Dentistry, Palacký University and University Hospital Olomouc, Olomouc 77900, Czech Republic; 6 Czech Advanced Technology and Research Institute, Palacký University, Olomouc 77900, Czech Republic; 7 Institute of Molecular and Translational Medicine, University Hospital Olomouc, Olomouc 77900, Czech Republic

**Keywords:** GPR54, KISS1 receptor, kisspeptin, triple-negative breast cancer (TNBC), targeted radionuclide therapy (TRT), radiotheranostics

## Abstract

Kisspeptins (KPs) and their receptor (KISS1R) promote metastasis and tumor progression in various cancers such as triple-negative breast cancer (TNBC). Targeting KISS1R holds great promise for molecular imaging and targeted radionuclide therapy of aggressively disseminated cancers. First ligand-based approaches using Ga-68/Lu-177-labeled KPs (KP-10, KP-54) have demonstrated feasibility but suffer from proteolytic degradation and low uptake in KISS1R positive tumors. However, lead structure optimization alone is insufficient, as KISS1R biology remains unexplored in a radiotheranostic context. In this study, *N*-terminally functionalized conjugates of KP-10, KP-54, and the hybrid peptide KiSS-34 (AMBA-2-Nal-Gly-Leu-Arg-Trp-NH_2_), including scrambled controls, were synthesized in high purity (≥95%) for comparative studies. The conjugation to 1,4,7,10-tetraazacyclododecane-1,4,7,10-tetraacetic acid (DOTA) and Alexa-Fluor-488 (AF-488) functionalities preserved biological activity, confirmed by (sub)­nanomolar EC_50_-values (0.05–0.85 nM) in calcium mobilization assays in transfected CHO-KISS1R cells. Conventional target detection methods using antibodies (Abs) and AF-488-KPs failed to visualize KISS1R in both model (CHO-KISS1R) and native cancer cell lines, likely due to unspecific Abs and rapid KISS1R internalization upon agonist stimulation. However, rapid KISS1R internalization was successfully visualized *via* live-cell imaging using AF-488-KP-10 and novel analogue AF-488-KiSS-34. Furthermore, DOTA-KPs were radiolabeled with Lu-177 in high efficiencies (≥95%) and examined in internalization assays, showing highest uptake (4.8%) and internalization rate (45.9%) for [^177^Lu]­Lu-DOTA-KiSS-34 in CHO-KISS1R cells compared to its KP-10 analogue (total uptake: 1.3%; internalization rate: 37.6%). Higher uptakes likely derive from faster binding kinetics, improved KISS1R targeting, and/or slower dissociation as evidenced by oil-based kinetics assays showing higher total uptake for [^177^Lu]­Lu-DOTA-KiSS-34 (15.3%) compared to KP-10 (3.8%) and KP-54 (4.5%) counterparts after 30 min. Positron emission tomography/computerized tomography (PET/CT) imaging, urine analysis, and all *in vitro* studies indicate that Ga-68/Lu-177-labeled DOTA-KiSS-34 exhibits superior pharmacodynamics, pharmacokinetics, and *in vivo* stability compared to its KP-10 and KP-54 analogues, which are critically suffering from rapid *in vivo* degradation. These results position DOTA-KiSS-34 as a strong structural lead for KISS1R-based radiotheranostics. Nevertheless, the dynamics between KPs and KISS1R need to be further investigated to fully harness the radiotheranostic potential of KISS1R for TNBC and other cancers.

## Graphical abstract

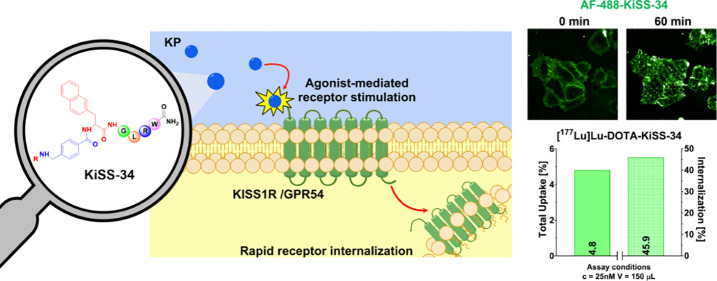

## 1 Introduction

Breast cancer (BCa) is one of the most prevalent cancers among women, with an estimated 2.3 million new cases and 685,000 deaths annually, making it the leading malignant tumor globally.^1,2^ Among these cases, triple-negative breast cancer (TNBC) accounts for approximately 15–20%,^3,4^ notorious for its aggressive nature, high mortality rates, and significantly lower 5-year survival rates compared to other BCa subtypes.^5,6^ TNBC is characterized by the absence of estrogen receptor α (ERα), progesterone receptor (PR), and human epidermal growth factor receptor 2 (HER2).^7^ The lack of targeted therapies against TNBC results in poor prognosis and limited survival due to early chemotherapy resistance and high metastasis rates,^8,9^ highlighting the urgent need for evaluation of novel theranostic targets.

Recent studies have revealed that KISS1R, a G-protein coupled receptor (GPCR), can be upregulated in selected cancer types, such as TNBC,^10^ hepatocellular carcinoma (HCC),^11^ renal cell carcinoma (RCC),^12^ and lung adenocarcinoma,^13^ verified at both RNA (RT-PCR) and protein (Western blot, immunohistochemistry) levels. In comparison, upregulation of KISS1R can exist in tumor tissue, while KISS1R expression remains relatively low in most healthy organs and tissues.^14^ This offers potential avenues for molecular imaging and targeted radionuclide therapy (TRT) options, both with regard to TNBC and further cancer types.

In detail, KISS1R is activated by its native ligands, kisspeptins (KPs, KiSS), derived from the *Kiss1* gene, which was first discovered in the late 1990s as a suppressor to melanoma cell metastasis.^15,16^ In humans, *Kiss1* is initially present as a 145-amino acid sequence that rapidly degrades into shorter peptides, including kisspeptin-54 (KP-54, metastin), kisspeptin-15 (KP-15), kisspeptin-14 (KP-14), and kisspeptin-10 (KP-10).^17^ KPs are categorized as RF-amides and possess an Arg^9^-Phe^10^-NH_2_ moiety, which is vital for KISS1R interaction to regulate the reproductive and pubertal axis in humans and animals.^17,18^

Despite its essential role in reproductive biology, the role and function of KISS1R in cancer biology remain controversial and not fully understood. This stems from both the cancer promoting and suppressing characteristics of KISS1R observed in different cancer types and stages. KISS1R has been reported to exhibit antimetastatic and/or antitumoral roles in numerous cancers, e.g., ovary, colorectal, pancreas, prostate, or thyroid.^19–22^ In contrast, high KISS1R upregulation in TNBC has been linked to increased tumor invasiveness, drug resistance, and tumor promotion.^10,23^ This paradoxical behavior necessitates the investigation of both KISS1R expression and localization to clarify its distinct role in cancer biology in order to advance drug development and physiological research.

Currently, the lack of highly specific antibodies (Abs) critically limits the evaluation of biological activity of KISS1R, resulting in inconsistent and unreliable results in KISS1R assessment.^14^ Based on their high-affinity binding, native KPs have been evaluated as alternative tools for Ab-based KISS1R detection. Hasegawa et al. have successfully used a FITC-labeled KP-14 derivative in Western Ligand Blot (WLB) and Ligand Derivative Stain (LDS) methods.^24^ The target visualizing potential of KP-54 has also been demonstrated in positron emission tomography (PET) imaging using a Ga-68-labeled NODAGA-KP-54 derivative.^25^ Additionally, first theranostic potentials of Lu-177-labeled DOTA-KP-10 have been assessed.^26^ Still, native KPs are prone to rapid *in vivo* degradation and unfavorable biodistribution,^24,27^ limiting their translational potential.

In previous studies, structural modifications of KP-10 have been reported to improve *in vivo* properties, e.g., proteolytic stability, receptor affinity, and bioactivity. This was achieved through substitutions with d-forms or unnatural amino acids and distinct synthetic alterations while keeping overall potency intact. Examples include (i) the Arg^9^-substitution with *N*-methylarginine to improve resistance to trypsin cleavage,^28^ (ii) Gly^7^-substitution with azaGly to increase metabolic stability and agonistic activity, further increased through combination with d-Trp^3^-insertion,^29^ (iii) insertion of triazoles between Gly^7^-Leu^8^ moieties, and (iv) *N*-terminal derivatization through acetylation^30^ or an albumin binding motif.^31^ These optimizations led to more stable and potent KP analogues, such as TAK-448,^32,33^ TAK-683,^33^ FTM080,^34^ or compound 34,^35^ simply named KiSS-34 in this article (Table 1).

**Table 1:** Compounds and Structures of KP-10 and Analogues with Improved Biological Properties

compound	structure
KP-10 ^17^	Tyr^1^-Asn^2^-Trp^3^-Asn^4^-Ser^5^-Phe^6^-Gly^7^-Leu^8^-Arg^9^-Phe^10^-NH_2_
TAK-448 ^32,33^	d-Tyr-Hyp-Asn-Thr-Phe-azaGly-Leu-Arg(Me)-Trp-NH_2_
TAK-683 ^33^	d-Tyr-d-Trp-Asn-Thr-Phe-azaGly-Leu-Arg(Me)-Trp-NH_2_
FTM080 ^34^	4-fluoro-benzoyl-Phe-Gly-Leu-Arg-Trp-NH_2_
KiSS-34 ^35^	AMBA-2-Nal-Gly-Leu-Arg-Trp-NH_2_

KiSS-34 is a hybrid hexapeptide agonist of comparable potency to KP-10, with structural replacements to the Ser^5^-Phe^6^ moiety through AMBA^5^ (4-aminomethylbenzoic acid) and 2-Nal^6^ (3-(2-naphthyl)-l-alanine). The latter modification suggests the presence of a large binding pocket in KISS1R. This optimization yielded high agonistic activity and offers improved pharmacokinetic properties due to its short peptide structure compared to those of KP-54 and KP-10. Thus, KiSS-34 is an interesting radiotheranostic lead against KISS1R-expressing cancers, which has not been assessed in previous studies.

In this study, we synthesized KiSS-34 and *N*-terminally functionalized chelator (DOTA) and dye (Alexa-Fluor-488; AF-488) conjugates to evaluate their potential in KISS1R detection and radiotheranostic applications. For comparative studies, chelator and dye analogues of KP-10 and KP-54 were successfully synthesized and evaluated in analogy, with scrambled controls for all KPs.

First, all conjugates were assessed for ligand potency in a CHO cell model engineered to express KiSS1R. Afterward, KISS1R target analyses were conducted on a model, TNBC and further native cancer cell lines using Abs and AF-488-KPs in conventional assays and live-cell imaging. Upon successful radiolabeling of DOTA-KPs with both Ga-68 and Lu-177, *in vitro* and *in vivo* properties were evaluated, including cell internalization assays in model and selected native cancer cell lines, oil-based kinetics assays, and PET/CT imaging in healthy BALB/c mice, followed by *in vivo* stability studies.

## 2 Experimental Section

### 2.1 Chemicals and Radionuclides

All chemicals (≥95% pure; ultrapure for radiolabeling) and solvents (HPLC-grade; metal-free for radiolabeling) were purchased from abcr, Bachem, Carbolution, CheMatech, Fluka, Iris Biotech, Lumiprobe, Macrocyclics, Merck Group, Carl Roth, or Sigma-Aldrich and were used as received unless noted otherwise. Silicon oil for high temperatures was purchased from Sigma-Aldrich. Pure mineral oil was purchased from Acros Organics. No-carrier-added ^177^LuCl_3_ in 0.04 M HCl was purchased from Isotope Technologies Munich SE (ITM), München, Germany.

### 2.2 Solid-Phase Peptide Syntheses (SPPS)

All peptide syntheses, including the scrambled controls, were conducted with an Applied Biosystems ABI 433A Peptide Synthesizer operated with SynthAssist 3.1 software. The FastMoc 0.10 mmol program was used running on a HBTU-mediated automated Fmoc protocol (amino acid (AA) (mmol) = 1.00, cycle time (min) = 24, waste per cycle (mL) = 50, ratio of AA:resin = 10:1) on 0.1 mmol of rink amide resin (155 mg, loading: 0.645 mmol/g, 100–200 mesh).

Next, the KP-functionalized resin was washed with 6 × 10 mL of Et_2_O and dried *in vacuo*. Subsequent cleavage from the resin was performed with a mixture of 3 mL of trifluoroacetic acid (TFA), 75 μL of distilled H_2_O, and 75 μL of triisopropylsilane (TIPS) for 4 h. The cleaved-off KP was precipitated in 50 mL of Et_2_O, centrifuged, decanted, taken up in 6–10 mL of acetonitrile (ACN):H_2_O (1:1, *v*/*v*), and filtrated using a syringe filtration cap (hydrophobic PTFE, 13 mm, 0.45 μm) prior to semipreparative HPLC purification.

### 2.3 Syntheses of DOTA and Alexa-Fluor-488 Conjugates

The syntheses of DOTA-KPs were performed on the KP-functionalized resin, resulting from Section 2.2. A syringe with 0.1 mmol of resin was loaded with a solution of 148 mg (3.92 equiv, 0.392 mmol) of HBTU, 115 mg (2 equiv, 0.2 mmol) of DOTA-tris­(*t*Bu)­ester, and 300 μL (1.7 mmol) of DIPEA in 3 mL of DMF and kept on a rotary mixer overnight. Next, the resin was washed with 6 × 10 mL of DMF, 6 × 10 mL of DCM, and 6 × 10 mL of Et_2_O and dried *in vacuo* afterward. Subsequent cleavage from the resin was performed with a mixture of 3 mL of TFA, 75 μL of distilled H_2_O, and 75 μL of TIPS for 4 h. The cleaved-off DOTA-KP was precipitated in 50 mL of Et_2_O, centrifuged, decanted, taken up in 6–10 mL of ACN:H_2_O (1:1, *v*/*v*), and filtrated using a syringe filtration cap (hydrophobic PTFE, 13 mm, 0.45 μm) prior to semipreparative HPLC purification.

AF-488-KPs were synthesized by addition of a solution containing 5 mg (0.06 mmol) of AF-488 NHS ester and 10 μL (0.056 mmol) of DIPEA in 200 μL of DMF or DMSO to 0.01 mmol of free KP in 150 μL of DMF or DMSO and subsequent stirring at room temperature overnight. Afterward, the reaction solution was taken up in 6–10 mL of ACN:H_2_O (1:1, *v*/*v*), sonicated in a warm water bath, if necessary to improve the solubility, and purified in analogy to the DOTA-KPs. All KP-54-based constructs were purchased from BioCat GmbH (Heidelberg, Germany).

### 2.4 Purification and Quality Control

Semipreparative HPLC was conducted using a LATEK P-402 pump coupled to a Merck Hitachi L-7420 UV/vis detector. The semipreparative purification of KPs and their DOTA conjugates was conducted using an Orbit 100 C18 RP-HPLC column (MZ Analysentechnik; 5 μm, 100 Å, 250 × 30 mm, MZ0901–2503000) with the following gradients, unless stated otherwise: (i) eluents: (A) water + 0.1% TFA and (B) ACN + 0.1% TFA; gradient: 0–40 min 5–95% B; flow: 30 mL/min; wavelength: 214 nm; temperature: RT; or (ii) eluents: (A) water + 0.1% TFA, (B) ACN + 0.1% TFA; gradient: 0–40 min 5–60% B; flow: 30 mL/min; wavelength: 214 nm; temperature: RT.

AF-488-KPs were purified using a NUCLEODUR HILIC HPLC-column (Macherey Nagel; 5 μm, 110 Å, 250 × 21 mm) with the following gradient, unless stated otherwise: Eluents: (A) water + 0.2% formic acid (FA), (B) ACN + 0.2% FA; gradient: 0–40 min 97–50% B; flow: 15 mL/min; wavelength: 214 nm; temperature: RT.

Unless noted otherwise, analytical HPLC was conducted by using a Thermo Fisher UltiMate 3000 HPLC with a variable wavelength detector. A Phenomenex Aeris Peptide 3.8u XB-C18 LC-column (Phenomenex; 3.6 μm, 100 Å, 150 × 4.60 mm) with the following gradient was used: (i) eluents: (A) water + 0.1% TFA, (B) ACN + 0.1% TFA; gradient: 0–17 min 5–95%, 17–22 min 95–5%, 22–24 min 5% B; flow: 0.8 mL/min; injection volume: 0.5 μL, wavelength: 214 nm, temperature: RT; or (ii) eluents: (A) water + 0.1% TFA, (B) ACN + 0.1% TFA; gradient: 0–12 min 5–95%, 12–15 min 95%, 15–18 min 95–5%, 18–20 min 5% B; flow: 0.8 mL/min; injection volume: 0.5 μL, wavelength: 254 nm, temperature: RT.

MS spectra were acquired using a Bruker Esquire 600 instrument equipped with an ion trap and an LT2 Plus instrument (Scientific Analysis Instruments, SAI) equipped with a time-of-flight (TOF) detector.

### 2.5 Cell Lines

Cell culture media and supplements were purchased from PAN Biotech, Gibco, and Sigma-Aldrich. The transfected CHO-KISS1R and Hep3B2 (hepatocarcinoma) cell lines were provided by Bayer AG (Wuppertal, Berlin). C33A (cervical cancer), LNCaP (prostate cancer), and SKOV3 (ovarian cancer) were purchased from the American Type Culture Collection (ATCC).

### 2.6 Cell Culture

*In vitro* assays were performed using the transfected cell line CHO-KISS1R and native C33A, Hep3B2, LNCaP, and SKOV3. The cell line was housed in a humidified atmosphere at 37 °C and 5% CO_2_. CHO-KISS1R was grown in a DMEM/F12 medium supplemented with 2% GlutaMAX, 2% HEPES, 2% sodium bicarbonate, 1.4% sodium pyruvate, 1% Pen/Strep, 10% FCS, and 1 mg/mL Geneticin (after recovery). C33A was grown in a DMEM/F12 medium supplemented with 2% GlutaMAX, 1% Pen/Strep, and 10% FCS. Hep3B2 was grown in MEM Earle’s medium supplemented with 1.1% GlutaMAX and 10% FCS. Finally, LNCaP and SKOV3 were grown in a RPMI-1640 medium supplemented with 10% FCS and 1.0% l-glutamine. Routine cell culture was performed twice a week using room-tempered phosphate-buffered saline (PBS; pH 7.4) for washing and 0.05% trypsin for cell detachment.

### 2.7 Calcium Mobilization Assay

4 × 10^3^ CHO-KISS1R cells, expressing the photoprotein mObelin, were seeded in 30 μL of medium per well on a black 384 Microtiter Plate (MTP) with a clear bottom and incubated overnight at 37 °C, 5% CO_2_. The next day, the medium was exchanged with 30 μL/well of 2 mM Ca-Tyrode and 5 μg/mL coelenterazine. Plates were incubated for 3 h at 37 °C, 5% CO_2_. KPs were diluted in 2 mM Ca-Tyrode in 4-fold concentration. 10 μL hereof was added to the cell plates in the FLIPR Tetra (Molecular Devices), and luminescence was measured immediately for 1 min. Every concentration was measured in quadruplicate. Values were normalized to controls without compound addition.

### 2.8 Fluorescence-Activated Cell Sorting (FACS) and Immunohistochemistry (IHC)

Transfected (CHO-KISS1R), control (CHO-luc hEP4), and native cancer cell lines (MCF-7, LNCaP, TALL-1, HepG2, VCaP, TT, NCI-H1781, NUGC-4, RPMI-8226, T-47D, MDA-MB-231, and MDA-MB-435) were used for flow cytometry assessment. 2 × 10^5^ cells per well were incubated with Abs and AF-488-KPs. For antibody staining, cells were treated with Fc-block, examined *via* PE-labeled secondary antibodies, and additionally checked with isotype controls. In dye-labeled staining, a FACS buffer was used instead. After washing steps, cells were resuspended in diluted Sytox Blue and were measured using a BD FACS Canto II instrument and analyzed using FlowJo software. All experiments were conducted in triplicate. Detailed experimental setups, cell lines, protocols, and all parameters are listed in SI2.1.

IHC studies were conducted by Nuvisan ICB GmbH (Berlin, Germany) using in-house best-practice protocols for Ab validation and tissue staining on formalin-fixed paraffin-embedded (FFPE) cell sections (NCI-H1048, CAL-51, HCT15, HT29, A549, and MDA-MB-453) and human tissue (placenta, colon). Ligand-based staining using AF-488-KPs (10, 100 μM) was conducted on cryo sections (5 μm) of RPMI-8226 cells and TALL-1 as control and scanned using a panoramic scanner. Detailed experimental setups, cell material, protocols, and all parameters are listed in SI2.2.

### 2.9 Proteomics/MS

For MS analysis, cells were grown to 70% confluency, washed 3 times with cold PBS, and then lysed in a buffer containing 1% SDS, 10 mM TCEP, 20 mM CAA, and 50 mM HEPES (pH 8.0). The lysates were heated to 90 °C for 10 min, and the DNA was sheered using a probe sonicator (Qsonica Q125). The protein concentration was determined by a BCA assay (Pierce).

The proteins were digested by protein aggregation capture (PAC) by mixing 28 μL of lysate (∼1 mg/mL protein concentration) with 2 μL of magnetic amine beads (ResynBio MR-AMN010) and 70 μL of acetonitrile. The beads were washed with 100 μL of 70% ACN in water, 100 μL of 80% ethanol in water, and 100 μL of 100% ACN. For digestion, 30 μL of 50 mM TEAB buffer (pH 8.0) containing 0.5 μg of trypsin (Serva) and 0.5 μg of LysC (Promega) was added, and the samples were incubated at 37 °C overnight. Peptides were acidified with 1% (final) TFA, and the beads were removed. 1 μg of peptides was analyzed on a Thermo Fisher UltiMate 3000 RSLC nano chromatograph coupled to a Thermo Fisher Orbitrap Eclipse MS. Peptides were separated on a 15 cm C18 column (PepSep) with 75 μm diameter and 1.5 μm particles with the following gradient: eluents: (A) water + 0.1% FA and (B) 80% ACN + 0.1% FA + 19.9% water; gradient: 0–30 min 8–24%, 30–42 min 24–38%, 42–47 min wash at 98% B; flow: 250 nL/min; temperature: 50 °C. The MS was operated in parallel reaction monitoring mode (PRM) targeting KISS1R-specific precursors listed in SI2.3. Orbitrap MS1 scans were acquired at 60k resolution, 300% AGC, and 20 ms max. injection time over a range of 250–1400 *m*/*z*. Precursors were isolated using a 0.8 *m*/*z* window, fragmented by HCD (30% NCE), and analyzed in the Orbitrap at 240k resolution, 700% AGC, and 502 ms max. injection time. PRM data were analyzed with Skyline 21.2.0.425 and R 4.5.1 software. Reported quantities are TIC-normalized peptide intensities.

### 2.10 Live-Cell Imaging

2.5 × 10^3^ CHO cells overexpressing human KISS1R were seeded in 384 well PhenoPlates (Revvity; 6057302) in 40 μL of DMEM/F12 medium per well, which was supplemented with 10% fetal bovine serum (FBS), 2% Glutamax, 1% penicillin/streptomycin (Pen/Strep), 2% sodium bicarbonate, 1.7% sodium pyruvate, and 2% HEPES. The cells were incubated overnight at 37 °C in a humidified atmosphere containing 5% CO_2_. On the following day, a series of dilutions of KPs were prepared in DMSO and subsequently diluted 10-fold in Tyrode buffer containing 2 mM calcium chloride and 0.01% bovine serum albumin. Cells were stained with Hoechst 33342 (final concentration: 0.35 μg/mL; Thermo Fisher Scientific: H3570) and PhenoVue Fluor 555 WGA (final concentration: 1 μg/mL; Revvity: CP15551) by adding 5 μL of a 10× stock solution prepared in the media. After 5 min of incubation, 5 μL from each KP concentration was transferred to the cell plate, and samples were imaged using Revvity’s Opera Phenix equipped with a 20× water immersion objective in confocal mode using the live-cell option at 37 °C and 5% CO_2_.

### 2.11 [^177^Lu]­Lu-Labeling and Quality Control

1.5–6.0 μL of ^177^LuCl_3_ (3.0–10.0 MBq/81.1–270.3 μCi) was transferred into a 1.5 mL Eppendorf tube (Protein LoBind), and 50 μL of NaOAc buffer (0.4 M, pH 5.0, Merck, Darmstadt, Germany) was added. All evaluated DOTA-KPs (10 mM in DMSO) were diluted to 1 mM with water (DMSO/water at 1:9 (*v*/*v*)), and 1.0–1.2 μL (1.0–1.2 nmol) was pipetted to the activity in NaOAc buffer (pH 5.0) and was incubated for 30 min at 95 °C. Quality control was performed by RP-TLC and radio-RP-HPLC. [^177^Lu]­Lu-DOTA-KP-10, [^177^Lu]­Lu-DOTA-KiSS-34, [^177^Lu]­Lu-DOTA-KP-54, [^177^Lu]­Lu-PSMA-617, and [^177^Lu]­Lu-DOTA-TATE were analyzed over silica gel 60 RP-18 plates (Merck, Darmstadt, Germany), serving as the stationary phase, while sodium citrate (0.1 M, Merck, Darmstadt, Germany) was used as the mobile phase. Radiolabeling efficiencies exceeded 96.5% for all DOTA-KPs. Hence, the resulting [^177^Lu]­Lu-DOTA-KPs were used without any further purification steps.

The radiolytic stability of [^177^Lu]­Lu-DOTA-KPs was determined 3 h postradiolabeling using a Thermo Fisher UltiMate 3000 HPLC equipped with a variable wavelength detector and an Elysia-Raytest Gabi flow cell gamma detector. Column: Chromolith C18 RP-HPLC end-capped, 2 μm, 130 Å, 100 × 4.6 mm; eluents: (A) water + 0.1% TFA, (B) ACN + 0.1% TFA; solvent gradient: 0–15 min 5–95% B; flow 2 mL/min; wavelength: 254 nm; temperature: RT.

### 2.12 Cellular Internalization Assay

The internalization rates of [^177^Lu]­Lu-DOTA-KP-10, [^177^Lu]­Lu-DOTA-KiSS-34, and [^177^Lu]­Lu-DOTA-KP-54 were examined on transfected CHO-KISS1R and native C33A, Hep3B2, and LNCaP cells. [^177^Lu]­Lu-PSMA-617 and [^177^Lu]­Lu-DOTA-TATE were used as controls on the CHO-KISS1R cells. The cells (5 × 10^5^ cells/well) were seeded in a 24-well plate 24 h before the experiment. In the case of LNCaP, the cells were seeded in analogy using a poly-l-lysine-coated 24-well plate. Incubation was performed 30, 60, or 120 min at 37 or 4 °C with 100, 50, 30, 25, or 10 nM of each [^177^Lu]­Lu-DOTA-KPs in 250 or 150 μL of Opti-MEM (Gibco). In particular cases, one set of cells was treated with blocking substances (KP-10, KiSS-34; 100, 50, 25, or 10 μM/well) to subtract the nonspecific binding.

After incubation, the activity was removed and cells were washed three times with 1 mL of ice-cold PBS. Surface-bound radioactivity was removed by incubating the cells twice with 500 μL of glycine buffer (50 mM; pH 2.8) for 5 min. After washing the cells once with 1 mL of ice-cold PBS, the internalized fraction was determined by subsequent cell lysis with 500 μL of NaOH (0.3 M; pH 14). The collected glycine and hydroxide fractions were measured in a gamma counter (Cobra Autogamma B5003, Canberra, Packard; Frankfurt, Germany) and calculated as percentage of total applied activity (% AA) specifically bound on either the cell surface or internalized inside the cells. The experiment was performed in triplicate.

### 2.13 Oil-Based Binding Kinetics Assay

The binding kinetics of [^177^Lu]­Lu-DOTA-KP-10, [^177^Lu]­Lu-DOTA-KiSS-34, and [^177^Lu]­Lu-DOTA-KP-54 were tested on CHO-KISS1R cells. The cells (5 × 10^6^ cells/Eppendorf tube (Protein LoBind)/compound) were incubated at 37 °C with 10 nM [^177^Lu]­Lu-DOTA-KP in 350 μL of Opti-MEM (Gibco) at five different incubation time-points (0, 5, 15, 30, and 60 min). After reaching the desired incubation period, 10 μL of the radiolabeled compound was transferred into a 0.4 mL PE reaction tube (Type Beckman) filled with a mixture of silicon-mineral oil (ratio 4:1). The tubes were centrifuged at 12,000 rpm for 2 min and immediately frozen in liquid nitrogen afterward. Subsequently, the tubes were cut approximately 0.5 cm from the bottom in order to separate the cell pellet (cell-bound radiolabeled compound) on the bottom of the tube and nonbound radiolabeled compound in the remaining part of the tube. The collected fractions were measured in a gamma counter (Cobra Autogamma B5003, Canberra, Packard; Frankfurt, Germany) and calculated as the percentage of total applied activity (% AA) specifically bound to CHO-KISS1R cells. The experiment was performed in quadruplicate.

### 2.14 [^68^Ga]­Ga-Labeling for PET/CT Imaging

Ga-68-labeling was performed using ^68^GaCl_3_ eluted from a ^68^Ge/^68^Ga generator (type IGG100, Eckert & Ziegler, Berlin, Germany) with 0.1 M HCl (Fluka, Buchs, Switzerland). A 5 μg (1 μg/μL) portion of each DOTA-KP was mixed with 30 μL of sodium acetate (1.14 M), and 300 μL of ^68^GaCl_3_ (50 MBq) was added. This labeling mixture (pH 3–4) was incubated for 15 min at 95 °C. Afterward, 100 μL of sodium acetate was added to increase the pH to 6–7 for suitable *in vivo* application. Quality control was performed by RP-HPLC using the Dionex UltiMate 3000 system (Thermo Scientific, Waltham, MA, USA) in combination with a radiometric detector (GABI Star, Raytest, Straubenhardt, Germany).

RP-HPLC was conducted using a Nucleosil 120–5 C18 column (250 × 40 mm, WATREX, Prague, Czech Republic) with the following gradients: eluents: (A) water + 0.1% TFA, (B) ACN + 0.1% TFA; gradient: 0–3 min 0%, 3–10 min 0–50%, 10–13 min 50–80%, 13–15 min 80–0% B; flow rate: 1 mL/min; wavelength: 225, 250 nm, temperature: RT.

### 2.15 PET/CT Imaging

All animal experiments were approved by the Czech Ministry of Education, Youth, and Sports (MSMT-35035/2019–3) and the Institutional Animal Welfare Committee of the Faculty of Medicine and Dentistry, Palacký University, Olomouc. The experiments were conducted in accordance with the regulations and guidelines set forth in the Czech Animal Protection Act (no. 246/1992). For this study, female 6–8-week-old BALB/c mice (Envigo, Horst, The Netherlands) were used and allowed to acclimatize to the laboratory conditions for a minimum of 1 week prior to initiation of the experiments. The mice were housed in individually ventilated cages on sawdust and had access to animal feed and water *ad libitum*.

PET/CT imaging studies were conducted in normal, healthy mice. The mice were anesthetized with isoflurane and were retro-orbitally (r.o.) injected with 100–150 μL of radiotracers corresponding to 4–6 MBq of radiotracer per animal. Mice were positioned prone in the Mediso NanoScan PET/CT small animal imaging system (Mediso Medical Imaging Systems, Budapest, Hungary), and static imaging was initiated 30 and 90 min postinjection (p.i.). A single PET field of view (FOV) of 98.5 mm was imaged for 10 min, followed by a whole-body helical CT scan (50 kVp/980 μA and 720 projections). The images were reconstructed using Mediso Tera-Tomo 3D PET iterative reconstruction (Mediso Medical Imaging Systems, Budapest, Hungary). Visualization and processing of the images were performed using a Mediso InterView FUSION (Mediso Medical Imaging Systems, Budapest, Hungary). The scans were normalized to the injected activity and animal weight.

### 2.16 *In Vivo* Stability Analysis of [^68^Ga]­Ga-DOTA-KPs

The experimental animals were intravenously injected with [^68^Ga]­Ga-DOTA-KPs (5 MBq, corresponding to 1–2 nmol of peptide per animal). Urine samples were collected 30 and 90 min postinjection and subsequently analyzed *via* HPLC in analogy to radiochemical purity assessments described in Section 2.14. Finally, the radiochromatograms of urine samples were compared with that of native [^68^Ga]­Ga-DOTA-KPs to assess *in vivo* stability.

## 3 Results

### 3.1 Synthesis and Analysis

All KPs were synthesized *via* SPPS and subsequently *N*-terminally conjugated to DOTA and AF-488 derivatives by HBTU-mediated on-resin coupling with DOTA-tris­(*t*Bu)­ester and an AF-488-NHS ester route with free KPs, respectively. The DOTA moiety was chosen for Ga-68/Lu-177-radiolabeling protocols, and the AF-488 moiety was chosen as a dye tag for target detection studies.

In the case of longer peptides, e.g., KP-10 and KP-54, DMSO offers improved solubility for AF-488 conjugation. The *N*-terminal triple Glu­(E)-derivatization of KP-10 and KiSS-34 was conducted to potentially improve the solubility and hydrophilicity for physiological conditions.

All compounds were synthesized with high purity (≥95%, Table 2), which was verified by analytical HPLC and ESI-MS. Furthermore, the main binding sequences (KP-10, KiSS-34) were scrambled and reacted to DOTA and AF-488 conjugates to serve as controls in biological assays. All further analyses, including scrambled controls, are listed in the Supporting Information (SI1).

**Table 2:** Overview of the Synthesized Main Pharmacophores for KISS1R Binding

sample	compound	structure	MW [g/mol]	yield [%]
KPs	KP-54 ^36^	Gly-Thr-Ser-Leu-Ser-Pro-Pro-Pro-Glu-Ser-Ser-Gly-Ser-Arg-Gln-Gln-Pro-Gly-Leu-Ser-Ala-Pro-His-Ser-Arg-Gln-Ile-Pro-Ala-Pro-Gln-Gly-Ala-Val-Leu-Val-Gln-Arg-Glu-Lys-Asp-Leu-Pro-Asn-Tyr-Asn-Trp-Asn-Ser-Phe-Gly-Leu-Arg-Phe-NH_2_	5857.5	--^a^
	KP-10 ^36^	Tyr-Asn-Trp-Asn-Ser-Phe-Gly-Leu-Arg-Phe-NH_2_	1302.4	53
	KP-10-EEE	Glu-Glu-Glu-Tyr-Asn-Trp-Asn-Ser-Phe-Gly-Leu-Arg-Phe-NH_2_	1689.8	49
	KiSS-34 ^35^	AMBA-2-Nal-Gly-Leu-Arg-Trp-NH_2_	860.0	21
	KiSS-34-EEE^(γ)^	(γ)Glu-Glu-Glu-AMBA-2-Nal-Gly-Leu-Arg-Trp-NH_2_	1247.4	20
DOTA-KPs	DOTA-KP-54	DOTA-Gly-Thr-Ser-Leu-Ser-Pro-Pro-Pro-Glu-Ser-Ser-Gly-Ser-Arg-Gln-Gln-Pro-Gly-Leu-Ser-Ala-Pro-His-Ser-Arg-Gln-Ile-Pro-Ala-Pro-Gln-Gly-Ala-Val-Leu-Val-Gln-Arg-Glu-Lys-Asp-Leu-Pro-Asn-Tyr-Asn-Trp-Asn-Ser-Phe-Gly-Leu-Arg-Phe-NH_2_	6243.8	--^a^
	DOTA-KP-10	DOTA-Tyr-Asn-Trp-Asn-Ser-Phe-Gly-Leu-Arg-Phe-NH_2_	1688.8	41
	DOTA-KP-10- EEE^(γ)^	DOTA-Glu-Glu-Glu-Tyr-Asn-Trp-Asn-Ser-Phe-Gly-Leu-Arg-Phe-NH_2_	2076.2	32
	DOTA-KiSS-34	DOTA-AMBA-2-Nal-Gly-Leu-Arg-Trp-NH_2_	1246.4	10
	DOTA-KiSS-34-EEE^(γ)^	DOTA-(γ)Glu-Glu-Glu-AMBA-2-Nal-Gly-Leu-Arg-Trp-NH_2_	1633.8	18
AF-488-KPs	AF-488-KP-54	AF-488-Gly-Thr-Ser-Leu-Ser-Pro-Pro-Pro-Glu-Ser-Ser-Gly-Ser-Arg-Gln-Gln-Pro-Gly-Leu-Ser-Ala-Pro-His-Ser-Arg-Gln-Ile-Pro-Ala-Pro-Gln-Gly-Ala-Val-Leu-Val-Gln-Arg-Glu-Lys-Asp-Leu-Pro-Asn-Tyr-Asn-Trp-Asn-Ser-Phe-Gly-Leu-Arg-Phe-NH_2_	6375.0	--^a^
	AF-488-KP-10	AF-488-Tyr-Asn-Trp-Asn-Ser-Phe-Gly-Leu-Arg-Phe-NH_2_	1819.9	6
	AF-488-KP-10- EEE^(γ)^	AF-488-Glu-Glu-Glu-Tyr-Asn-Trp-Asn-Ser-Phe-Gly-Leu-Arg-Phe-NH_2_	2207.3	2
	AF-488-KiSS-34	AF-488-AMBA-2-Nal-Gly-Leu-Arg-Trp-NH_2_	1376.5	7
scrambled controls	KP-10s	Ser-Tyr-Phe-Asn-Trp-Asn-Phe-Arg-Leu-Gly-NH_2_	1302.4	34
	AF-488-KP-10s	AF-488- Ser-Tyr-Phe-Asn-Trp-Asn-Phe-Arg-Leu-Gly-NH_2_	1819.9	7
	DOTA-KP-10s	DOTA-Ser-Tyr-Phe-Asn-Trp-Asn-Phe-Arg-Leu-Gly-NH_2_	1688.8	14
	KiSS-34s	Leu-Trp-2-Nal-Arg-AMBA-Gly-NH_2_	860.0	36
	AF-488-KiSS-34s	AF-488-Leu-Trp-2-Nal-Arg-AMBA-Gly-NH_2_	1376.5	11
	DOTA-KiSS-34s	DOTA-Leu-Trp-2-Nal-Arg-AMBA-Gly-NH_2_	1246.4	14

a Commercially purchased.

### 3.2 Ligand Potency Assessment

In a calcium mobilization assay, the ligand potencies of selected KPs and *N*-terminal conjugates were examined in the transfected cell line CHO-KISS1R overexpressing the target receptor (Table 3). The ligand affinity toward KISS1R sites is not influenced by *N*-terminal derivatization. Overall, high affinity was observed for all tested ligands in the (sub)­nanomolar range.

**Table 3:** Ligand Potencies of KPs and Their AF-488 and DOTA Conjugates Assessed on a Transfected CHO-KISS1R Cell Line

KP derivative	EC_50_ ^a^ [M]
KP-10	8.50 × 10^–10^
AF-488-KP-10	5.10 × 10^–11^
DOTA-KP-10	1.20 × 10^–10^
AF-488-KP-10-EEE	1.50 × 10^–10^
DOTA-KP-10-EEE	1.90 × 10^–10^
KP-54	4.55 × 10^–10^
AF-488-KP-54	5.65 × 10^–10^
DOTA-KP-54	6.05 × 10^–10^
AF-488-KiSS-34	7.30 × 10^–11^
DOTA-KiSS-34	8.90 × 10^–11^
DOTA-KiSS-34-EEE^(γ)^	2.10 × 10^–10^

a EC_50_: half-maximal effective concentration.

### 3.3 Target Detection Studies

KISS1R expression was assessed using FACS and IHC. Using commercially available antibodies and AF-488-KP-10, no detectable or specific binding to KiSS1R was observed in transfected CHO-KISS1R, TNBC (FACS: MDA-MB-231; IHC: CAL51), and native cancer cells (FACS: MCF-7, LNCaP, TALL-1, HepG2, VCaP, TT, NCI-H1781, NUGC-4, RPMI-8226, T-47D, MDA-MB-435; IHC: NCI-H1048, HCT15, HT29, A549, and MDA-MB-453), consistent with previously reported challenges in KISS1R detection. In our target detection studies, we screened multiple cell lines, including different cancers, to better understand the KISS1R expression profiles and receptor dynamics.

As binding affinities of KP derivatives were successfully confirmed in the transfected CHO-KISS1R cell line, we wanted to confirm that our FACS and IHC studies failed due to methodological limitations. For this purpose, high-sensitivity proteomics analyses were performed, which successfully detected KISS1R proteins in both transfected CHO-KISS1R cells and native cell lines (Hep3B2, NCI-H1048, RPMI 8226, and VCaP; Figure 1). In remaining native cell lines, the KISS1R protein levels were below the detection limit (n.d.). As expected, transfected CHO-KISS1R cells showed a higher expression compared to the native cells. Regardless, both antibody– and ligand–receptor interactions were not observed, possibly due to complex receptor dynamics.

**Figure 1:**
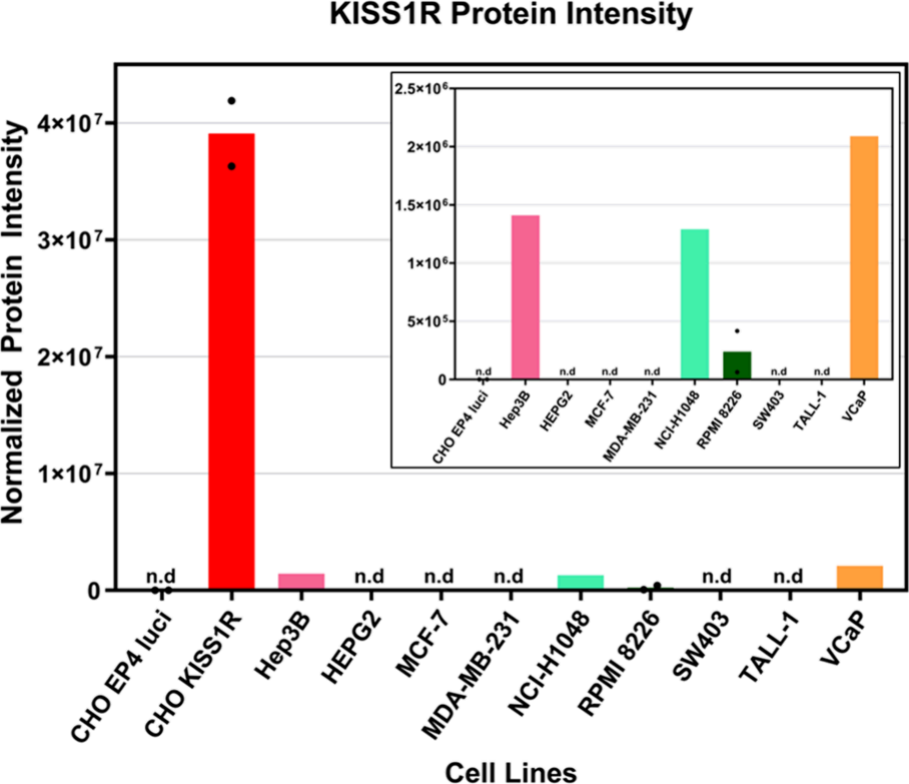
Identification of KISS1R positive cell lines through proteomics analyses by MS. Protein intensities with and without (inset) transfected CHO-KISS1R.

Under these conditions, we concluded that FACS and IHC alone are insufficient to evaluate the KISS1R mechanisms and behavior. We hypothesized rapid KISS1R internalization dynamics upon stimulation with agonists,^37,38^ resulting in methodological and experimental limitations. Hence, live-cell microscopy was used to study KISS1R and successfully visualized rapid internalization dynamics in transfected CHO-KISS1R cells upon treatment with AF-488-KP-10. Here, a rapid accumulation of AF-488-KP-10 at the plasma membrane of CHO-KISS1R cells is observed, followed by a fast internalization into vesicular structures over a time frame of 60 min (Figure 2). This process was accompanied by clear colocalization with wheat germ agglutinin (WGA).

**Figure 2:**
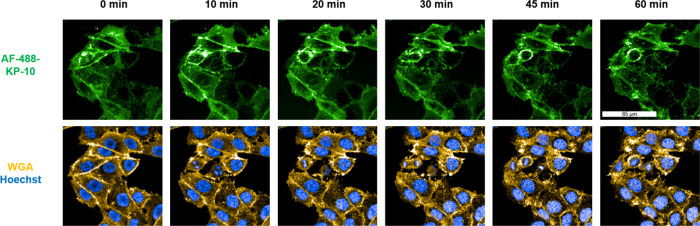
Binding and internalization of AF-488-KP-10 in transfected CHO-KISS1R cells through KISS1R; the cells were treated with 100 nM AF-488-KP-10 (green) and analyzed *via* live-cell microscopy over 60 min. The overlay images show the plasma membrane and endocytic vesicles, WGA (yellow), and nuclei (blue).

In addition, we conducted the same experiment using AF-488-KiSS-34 (Figure 3) and concluded that both AF-488-KP-10 and AF-488-KiSS-34 are accumulated in a similar fashion.

**Figure 3:**
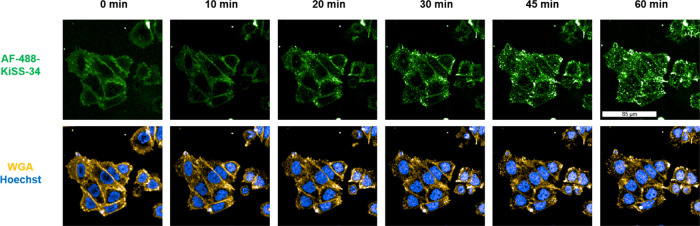
Binding and internalization of AF-488-KiSS-34 in transfected CHO-KISS1R cells through KISS1R; the cells were treated with 100 nM AF-488-KiSS-34 (green) and analyzed *via* live-cell microscopy over 60 min. The overlay images show the plasma membrane and endocytic vesicles, WGA (yellow), and nuclei (blue).

Control experiments confirmed the KISS1R specificity of this interaction, as no internalization of AF-488-KP-10 and AF-488-KiSS-34 was observed in CHO-WT cells lacking KISS1R expression (Figure 4). Additionally, scrambled KPs and their AF-488 conjugates showed no binding or internalization in CHO-KISS1R cells, indicating the structural specificity of KISS1R toward RF-amides.

**Figure 4:**
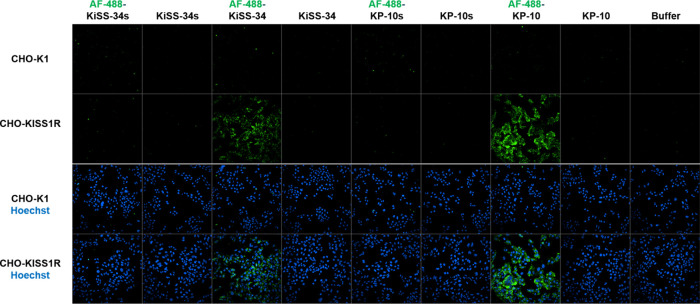
Specificity assessment of AF-488-KP-10 and AF-488-KiSS-34 compared to unlabeled (KP-10, KiSS-34) and scrambled controls (AF-488-KP-10s, AF-488-KiSS-34s) in transfected CHO-KISS1R and KISS1R-negative CHO-K1 cells. Cells were treated with 10 nM AF-488-KPs and incubated for 3 h before live-cell microscopy. The overlay images show the KISS1R (green) and nuclei (blue).

The specificity of KISS1R toward RF-amides was confirmed in CHO-KISS1R cells with siRNA-mediated knockdown of KISS1R (SI2.4,Figure S4). Here, no binding and internalization of examined AF-488-KPs were observed. Additionally, we expanded this setup to native cancer cell lines (NCI-H1048 and Hep3B2), in which KISS1R expression was confirmed by our proteomics studies. In these cell lines and siRNA knockdown variants, no KISS1R-specific binding or internalization of AF-488-KP-10 was observed. This could indicate that KISS1R expression levels in native cells may fall below the functional detection threshold of live-cell microscopy. The assay specificity was confirmed *via* scrambled control AF-488-KP-10s, which showed no signals in all examined cell lines.

### 3.4 Lu-177-Labeling and Radiolytic Stability Assessment

Having confirmed rapid KISS1R-specific internalization dynamics *via* live-cell microscopy, we aimed to quantitatively assess the internalization using Lu-177-labeled DOTA-KPs. Quality control by RP-TLC gave high radiolabeling efficiencies of ≥95% for all DOTA-KPs (Table 4) without the need for further necessary purification steps. In the first Lu-177-labeling procedures, EEE-modified DOTA-KPs consistently failed to reach required radiochemical purity (≥95%), despite application of identical radiolabeling protocols. Furthermore, rapid metabolic degradation of Ga-68-labeled EEE-analogues was observed in preliminary imaging studies (data not shown). As a result, EEE-DOTA-KPs have not been examined any further.

**Table 4:** Lu-177-Labeling Efficiencies of DOTA-KPs

DOTA-KP	mean radiolabeling efficiency [%]
[^177^Lu]Lu-DOTA-KP-10	99.1 (*n* = 8)
[^177^Lu]Lu-DOTA-KiSS-34	99.7 (*n* = 10)
[^177^Lu]Lu-DOTA-KP-54	96.5 (*n* = 4)

Overall, the compounds showed good radiolytic stability and no signs of Lu-177-decomplexation 3 h postradiolabeling (Figure 5), which is favorable for subsequent biological examinations.

**Figure 5:**
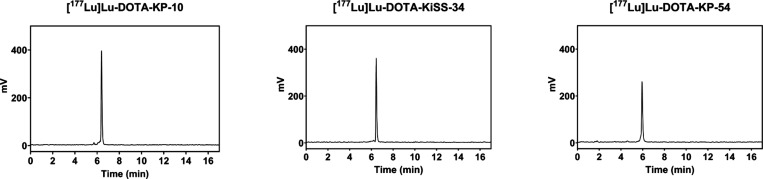
Radiolytic stability of [^177^Lu]­Lu-DOTA-KP-10, [^177^Lu]­Lu-DOTA-KiSS-34, and [^177^Lu]­Lu-DOTA-KP-54 3 h postradiolabeling determined by radio-HPLC.

### 3.5 Internalization Assays

The specific binding of Lu-177-labeled DOTA-KP-10 and DOTA-KiSS-34 to KISS1R was examined by internalization assays in transfected (CHO-KISS1R) and selected native cancer cells (LNCaP, C33A, and Hep3B2). In previous studies, KISS1R-positive LNCaP tumors were visualized using a KP-54-based radioligand,^25^ and we therefore chose to examine [^177^Lu]­Lu-DOTA-KiSS-34 and [^177^Lu]­Lu-DOTA-KP-10 in LNCaP, Hep3B2, and C33A^39^ cells, reported to show KISS1R expression. DOTA-KP-54 was excluded from this study due to well-known limitations such as reduced tumor penetration, lower internalization, and overall slower pharmacokinetics.

First, ideal conditions for the internalization assays were determined in CHO-KISS1R, as the results can be highly dependent on the concentration of the applied radioligands and the total volume of cell medium added per well. Therefore, setups with different radioligand concentrations (100, 25, 10 nM), incubation times (0.5, 1, and 2 h), and cell medium volumes (Opti-MEM; 250, 150 μL) were examined (SI3, Figures S5–S7). At the 1 h incubation mark, using lower radioligand concentrations (25 nM) in combination with lower total volumes (150 μL) resulted in a 2- to 3-fold increase of uptake with slightly higher internalization rates for both DOTA-KPs (Figure 6).

**Figure 6:**
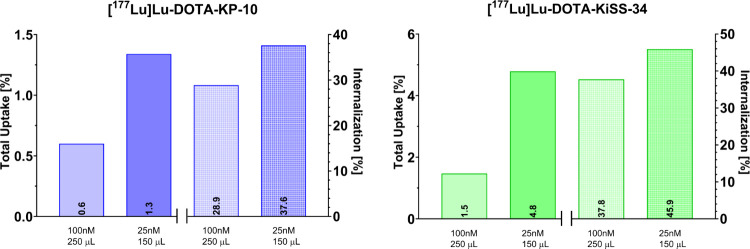
Cellular uptake of [^177^Lu]­Lu-DOTA-KP-10 and [^177^Lu]­Lu-DOTA-KiSS-34 in CHO-KISS1R cells at different concentrations (100, 25 nM) and different total cell medium volumes (Opti-MEM; 250, 150 μL) incubated for 1 h at 37 °C. Uptake represents the total amount of bound radioligand (extra- and intracellularly), whereas internalization rates reflect the percentage of total uptake internalized into the cell.

At these conditions, [^177^Lu]­Lu-DOTA-KiSS-34 showed the highest uptake of 4.8% and an internalization rate of 45.9% compared to [^177^Lu]­Lu-DOTA-KP-10 (total uptake: 1.3%; internalization rate: 37.6%). Uptake and internalization rates reached their optimum at the 1 h incubation mark, followed by a noticeable decrease after 2 h (SI3, Figure S7). The impact of specific activity on total binding and internalization rates was ruled out, as shown by experiments using different specific activities (5, 10 MBq/nmol) with [^177^Lu]­Lu-DOTA-KP-10 (SI3, Figure S8, Figure 6). In all instances, total uptake and internalization rates were higher for [^177^Lu]­Lu-DOTA-KiSS-34.

With an increased ligand–receptor specificity at lower concentrations, subsequent assays were therefore performed with a radioligand concentration of 10 nM, a total Opti-MEM volume of 150 μL, and an incubation period of 1 h. At these conditions, the total uptake of [^177^Lu]­Lu-DOTA-KP-10 (0.6%, Figure 7A) was notably lower compared to that of [^177^Lu]­Lu-DOTA-KiSS-34 (2.4%, Figure 7B) in CHO-KISS1R cells.

**Figure 7:**
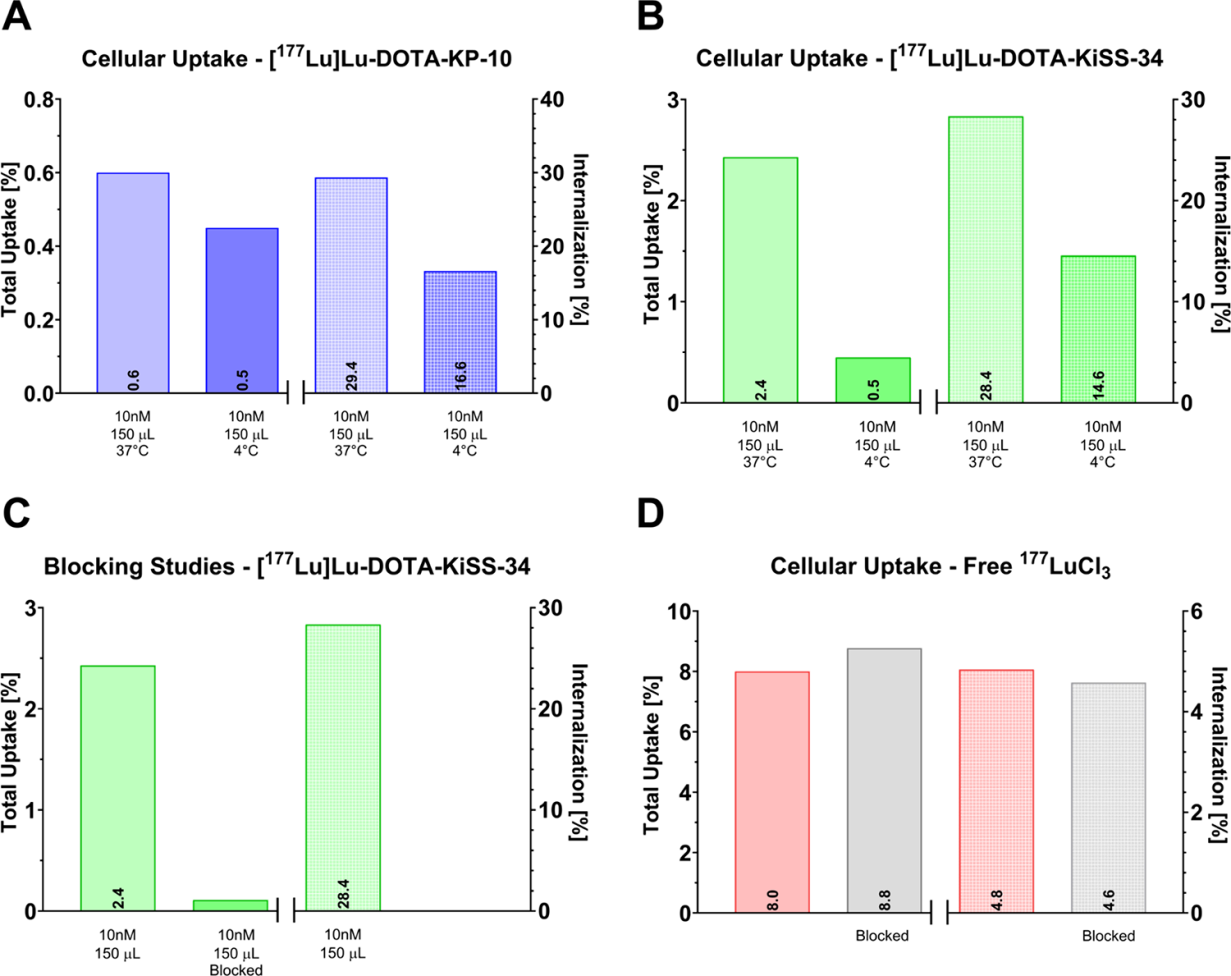
Cellular uptake of (A) [^177^Lu]­Lu-DOTA-KP-10 and (B) [^177^Lu]­Lu-DOTA-KiSS-34 (10 nM) in CHO-KISS1R cells incubated for 1 h at 4 and 37 °C. (C) Blocking studies of [^177^Lu]­Lu-DOTA-KiSS-34 in CHO-KISS1R cells using 10 μM KiSS-34 as a blocking agent. (D) Cellular uptake of free ^177^LuCl_3_ in CHO-KISS1R cells.

Furthermore, the KISS1R-mediated specificity and energy dependency of this interaction were examined by comparison of uptake and internalization at different incubation temperatures (37 °C, 4 °C). At lower temperatures, a strong decrease was observed for [^177^Lu]­Lu-DOTA-KP-10 (uptake: 0.6% to 0.5%; internalization: 29.4% to 16.6%) and [^177^Lu]­Lu-DOTA-KiSS-34 (uptake: 2.4% to 0.5%; internalization: 28.4% to 14.6%) in CHO-KISS1R cells (Figure 7A,B). These results indicate a strong but partial energy dependency. Blocking studies further confirmed the KISS1R-mediated specificity of applied radioligands. In CHO-KISS1R cells, the uptake of [^177^Lu]­Lu-DOTA-KiSS-34 was blocked by addition of 10 μM KiSS-34 (Figure 7C). Internalization assays with negative controls ([^177^Lu]­Lu-PSMA-617 – Pluvicto, [^177^Lu]­Lu-DOTA-TATE – Lutathera) showed no binding (total uptake: <0.09%; internalization: <0.65%) in CHO-KISS1R cells, highlighting the specificity of examined [^177^Lu]­Lu-DOTA-KPs.

Next, the importance of high radiolabeling efficiencies was investigated by the cellular uptake of free, noncoordinated ^177^LuCl_3_ (Figure 7D) in CHO-KISS1R cells. Here, a substantial uptake (8.0%) was observed, albeit with much lower internalization rates (<5%), which was not blocked by addition of KiSS-34. This hints a nonspecific binding mechanism, which is KISS1R-independent and stresses the importance of high radiolabeling efficiencies, which we readily achieved. At this point, potential contributions from media components such as Opti-MEM cannot be excluded and require further investigation.

Taking all data into consideration, we hypothesized that a higher uptake of [^177^Lu]­Lu-DOTA-KiSS-34 might result from faster binding kinetics, especially when compared to its KP-10 counterpart. To validate this, we performed an oil-based kinetics assay on transfected CHO-KISS1R cells using Lu-177-labeled DOTA-KP-54, -KP-10, and -KiSS-34 (Table 5).

**Table 5:** Total Uptake Assessment of [^177^Lu]­Lu-DOTA-KPs in an Oil-Based Binding Kinetics Assay Using Transfected CHO-KISS1R Cells^a^

	total uptake [%]		
time [min]	**[** ^177^ **Lu]Lu-DOTA-KP-54**	**[** ^177^ **Lu]Lu-DOTA-KP-10**	**[** ^177^ **Lu]Lu-DOTA-KiSS-34**
0	2.1	0	0
5	4.1	7.8	10.2
15	4.6	7.0	11.6
30	4.5	3.8	15.3
60	4.3	3.4	9.2

a 5 × 10^6^ cells; conducted in a mixture of silicon-mineral oil (4:1 ratio), *c* ([^177^Lu]­Lu-DOTA-KP) = 10 nM.

Overall, [^177^Lu]­Lu-DOTA-KiSS-34 exhibited much higher total uptakes compared with KP-10- and KP-54-based derivatives. The total uptake of [^177^Lu]­Lu-DOTA-KiSS-34 peaked after 30 min (15.3%) and was higher compared to the larger [^177^Lu]­Lu-DOTA-KP-10 (3.8%) and [^177^Lu]­Lu-DOTA-KP-54 (4.5%), which is in line with our expectations. The KISS1R-mediated specificity of this interaction was confirmed in KISS1R-negative SKOV3 cells (SI4, Table S12).

In the next step, we expanded our experimental setup to native cancer cell lines. In LNCaP cells, [^177^Lu]­Lu-DOTA-KiSS-34 and [^177^Lu]­Lu-DOTA-KP-10 demonstrated high total uptakes but notably low internalization rates of <5% (Figure 8A,B) compared to transfected CHO-KISS1R cells. However, blocking studies on LNCaP cells indicated a non-KISS1R specific uptake mechanism, as 10 μM KISS-34 did not displace the uptakes in LNCaP cells at all (Figure 8C). In Hep3B2 and C33A cells, very low uptakes were observed, indicating a very low level of KISS1R expression (Figure 8D).

**Figure 8:**
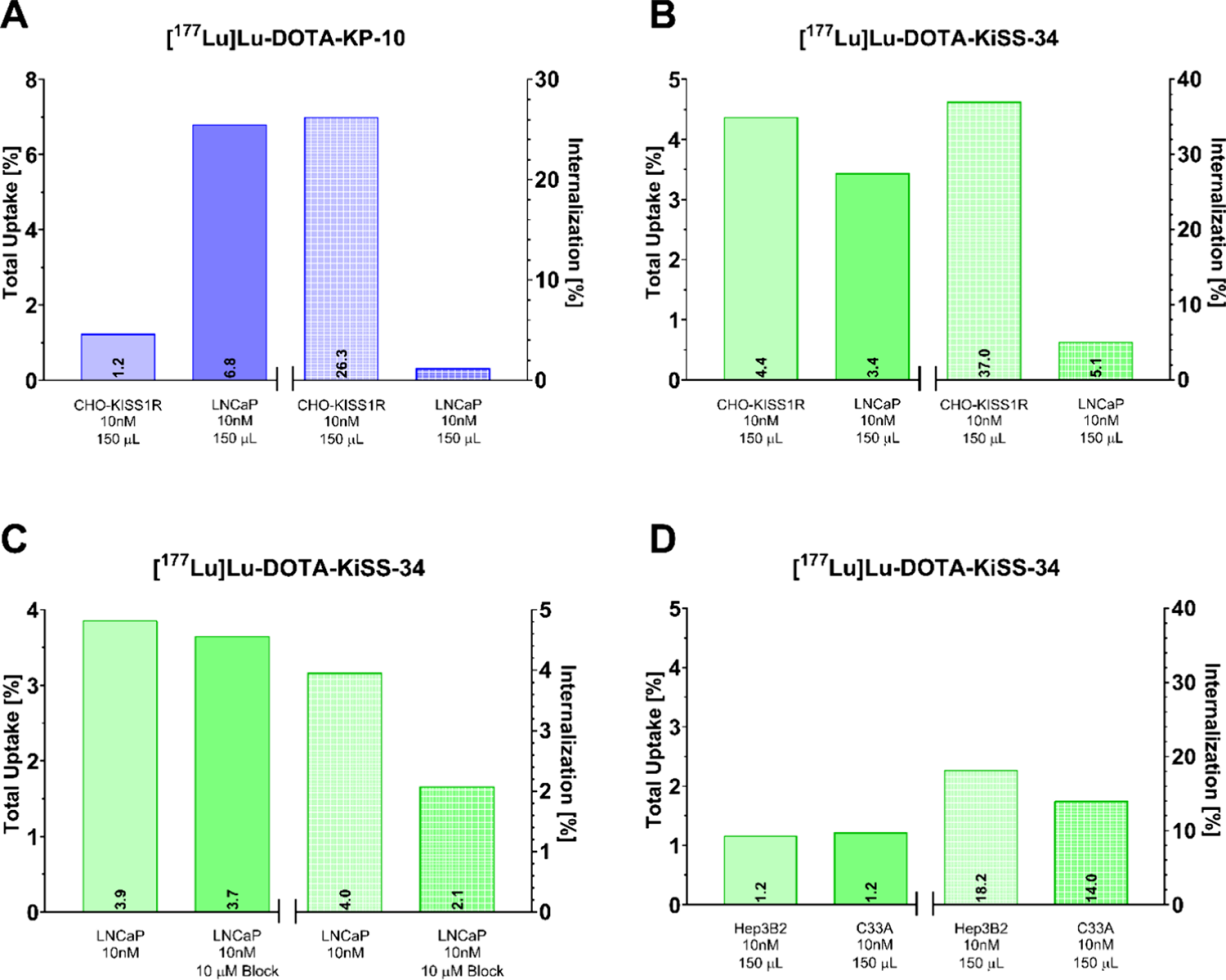
Cellular uptake of (A) [^177^Lu]­Lu-DOTA-KP-10 and (B) [^177^Lu]­Lu-DOTA-KiSS-34 (*c* = 10 nM) in LNCaP cells in comparison to CHO-KISS1R cells. (C) Blocking studies of [^177^Lu]­Lu-DOTA-KiSS-34 using 10 μM KiSS-34 as a blocking agent in LNCaP cells. (D) Cellular uptake of [^177^Lu]­Lu-DOTA-KiSS-34 in Hep3B2 and C33A cells.

### 3.6 PET/CT Imaging

The Ga-68-radiolabeling of DOTA-KP-10, DOTA-KiSS-34, and DOTA-KP-54 was performed in high radiochemical purity ranging from 96.1% to 97.8%. In addition, the *in vivo* stability of [^68^Ga]­Ga-DOTA-KiSS-34 was compared to that of its KP-10 and KP-54 counterparts.

PET/CT imaging of [^68^Ga]­Ga-DOTA-KPs revealed visibly different tissue distribution profiles in healthy BALB/c mice (Figure 9). [^68^Ga]­Ga-DOTA-KP-54 showed strong and prolonged kidney retention. A structural reduction to the first 10 amino acids (KP-10) resulted in a severely decreased retention and faster renal clearance. In the case of [^68^Ga]­Ga-DOTA-KiSS-34, bearing only six amino acids, similar rapid renal clearance was observed. However, partial liver accumulation was present with [^68^Ga]­Ga-DOTA-KiSS-34 in contrast to Ga-68-labeled DOTA-KP-54 and DOTA-KP-10, hypothesized to stem from lipophilic amino acids (AMBA-2-Nal).

**Figure 9:**
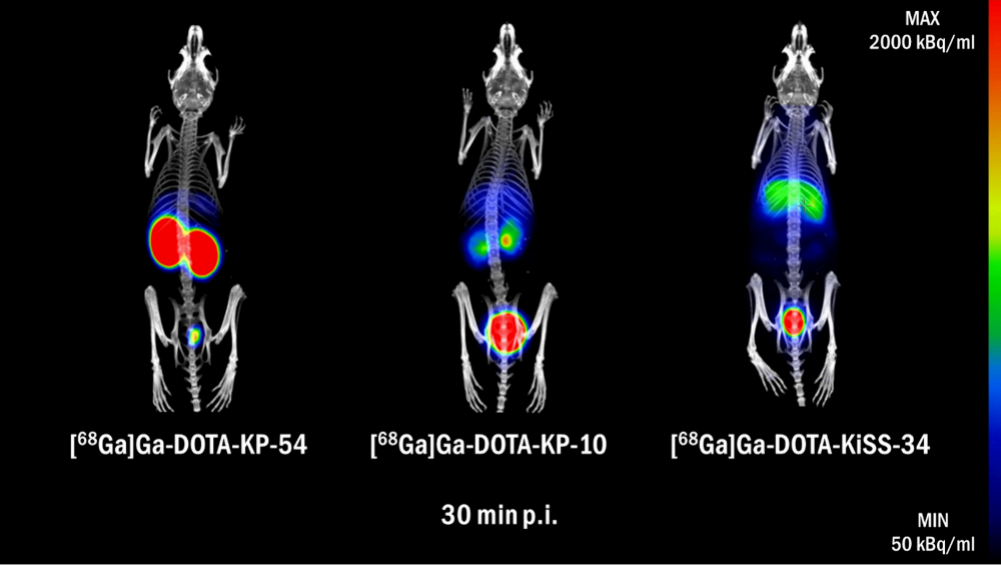
PET/CT images of healthy BALB/c mice injected with [^68^Ga]­Ga-DOTA-KP-54, [^68^Ga]­Ga-DOTA-KP-10, and [^68^Ga]­Ga-DOTA-KiSS-34. Images are presented as maximum intensity projections of fused PET and CT at 30 min p.i.

*In vivo* stability was assessed through p.i. urine analyses *via* HPLC (Figure 10). In their respective urine analyses, [^68^Ga]­Ga-DOTA-KP-54 and [^68^Ga]­Ga-DOTA-KP-10 showed clear evidence of degradation and altered retention times, indicating a significant *in vivo* effect on examined compounds. However, [^68^Ga]­Ga-DOTA-KiSS-34 remained stable with unchanged retention times under these conditions, indicating superior pharmacokinetics compared with native KP-based constructs. Based on reduced renal retention and improved metabolic stability, DOTA-KiSS-34 may serve as the most suitable candidate for further *in vivo* evaluations.

**Figure 10:**
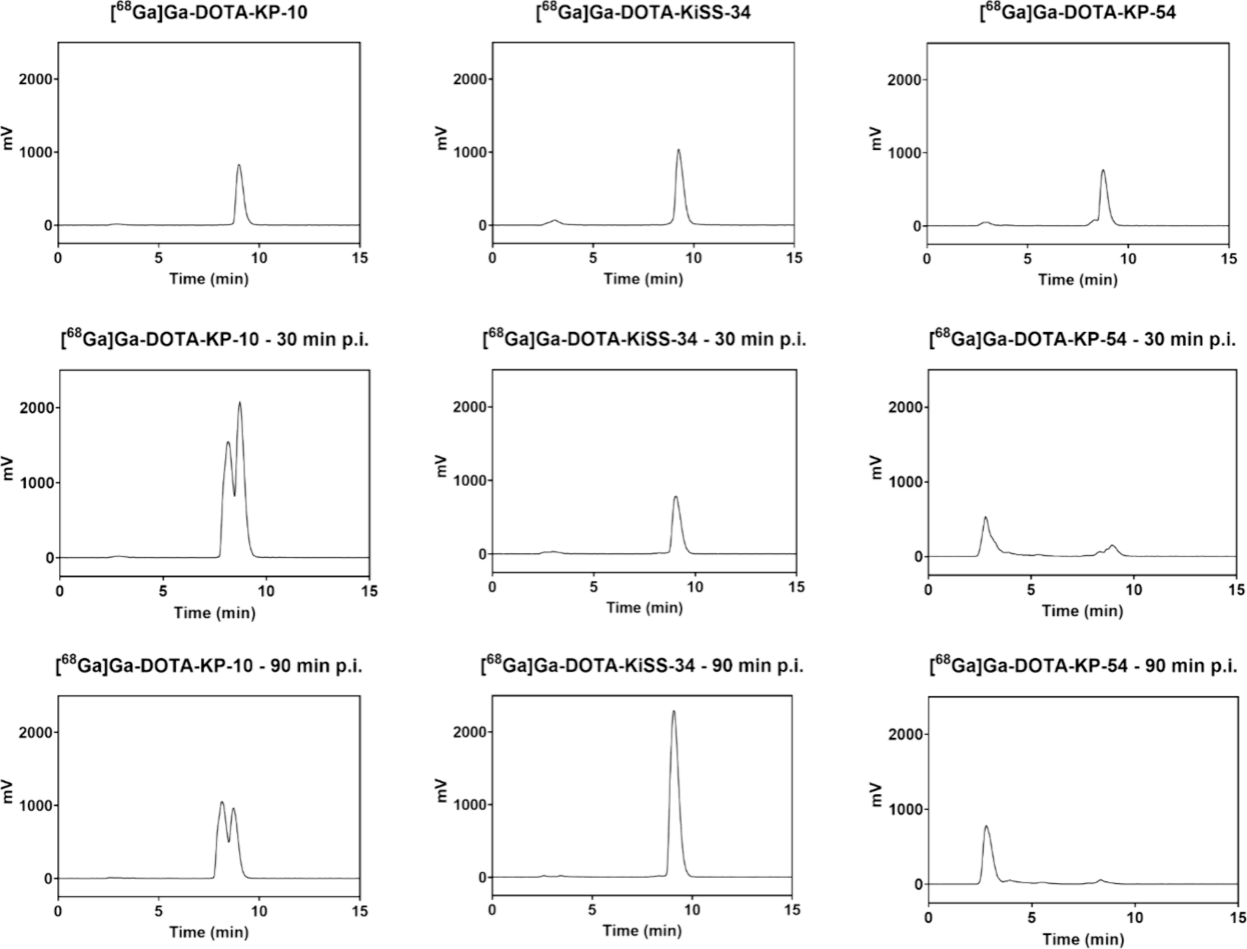
*In vivo* stability analysis of Ga-68-labeled DOTA-KPs (KP-10, KiSS-34, KP-54) in mouse urine using HPLC. Time points are (i) immediately after radiolabeling and (ii) 30 and (iii) 90 min p.i.

## 4 Discussion

The KP/KISS1R interaction has been well-researched in reproductive biology but remains elusive in cancer biology, complicating the full use of its radiotheranostic potential. Abs lack specificity toward KISS1R,^14,24^ as commonly observed with other GPCRs. Targeted epitopes can show different forms, conformations, and expressions, complicating the development of specific Abs. Furthermore, GPCR-specific Abs frequently show cross-reactions with other GPCRs, resulting in false positives.^40,41^ As a result, ligand-based concepts are often favored in receptor studies. Here, the use of KPs has resulted in successful visualization of KISS1R expression, offering an alternative to conventional target detection methods.^24,25,37^ Most recently, Ga-68-labeled DOTA-conjugates of KISS1-305 and -TAK-683 were reported as potential PET agents, showing promising binding affinity, tumor uptake, and tumor-to-normal organ uptake ratios in a transfected HEK293 cell line.^42^

These results support the idea that the interaction of KP/KISS1R holds radiotheranostic promise. However, the nature of this interaction is highly complex, contradictory, and cancer-dependent, which must be considered in further evaluations. In TNBC, KISS1R is reported to be upregulated, but the underlying mechanism remains elusive.^22^ Hence, binding dynamics, receptor behavior, and detection challenges in KISS1R-based theranostics must be systematically investigated. In this study, we examined these aspects using AF-488- and DOTA-KPs in transfected and different native cancer cell models. *N*-terminally derivatized KPs were obtained without loss in overall potency, congruent to previous studies.^43–46^ In our target detection studies, Abs and AF-488-KPs failed to reliably detect KISS1R in both transfected and native models, with TNBC and further cancers included. Abs are known to be highly unspecific;^14^ however, this should not be the case with KP-based constructs.

In Western Ligand Blot studies, Hasegawa et al. identified three different KISS1R forms of 43, 72, and 86 kDa crucial for KP binding, while Abs detected only a single, specific KISS1R epitope (54 kDa).^24^ Abs and small molecules can target different epitopes, leading to substantial differences in the binding and subsequent receptor activation. Recent Cryo-EM studies revealed different KISS1R molecular binding profiles among various KPs (KP-54, TAK-448), highlighting unique receptor conformations and activation mechanisms. *C*-terminal RF-amides were confirmed to be highly relevant for specific KISS1R binding, coupled to different G-protein-subunits G_q_ or G_i_, possibly resulting in activation of different signaling pathways.^47^ As binding to KISS1R and subsequent activation were evident in calcium mobilization assays, we excluded the possibility of weak receptor engagement by KPs in transfected cells.

We hypothesized that our negative results stem from rapid KISS1R internalization upon stimulation with agonists,^37,38^ resulting in methodological and experimental limitations. Pampillo et al.^37^ have reported an 80% loss of cell-surface receptor after 5 min of KP-10-stimulation, which is supported by our live-cell imaging studies using AF-488-KPs. However, a quantitative assessment of bound and internalized AF-488-KPs remains challenging due to possible biological and technical limitations. Lower and heterogeneous KISS1R expression might limit the sensitivity of fluorescence-based imaging in native cancer cells, as observed in NCI-H1048 and Hep3B2 cells, necessitating method optimization for future studies. To our knowledge, internalization dynamics of KISS1R have not been considered in the radiotheranostic context yet and must be elucidated prior to translation to TNBC models.

Radioactivity offers a higher sensitivity compared to fluorescence-based methods. Therefore, we first examined KISS1R dynamics in internalization assays using Lu-177-labeled DOTA-KPs, obtained with high labeling efficiency (≥95%) and purity (≥99%). In CHO-KISS1R cells, [^177^Lu]­Lu-DOTA-KiSS-34 showed higher uptake (4.8%) and internalization rates (45.9%) compared to [^177^Lu]­Lu-DOTA-KP-10 (uptake: 1.3%; internalization: 37.6%).

Improved pharmacokinetics and -dynamics were hypothesized for [^177^Lu]­Lu-DOTA-KiSS-34, which were confirmed by oil-based kinetics assays. After 5 min, [^177^Lu]­Lu-DOTA-KiSS-34 showed higher uptake (10.2%) compared to KP-10 (7.8%) and KP-54 (4.1%) analogues, followed by a delayed peak uptake at 30 min (15.3%). Faster kinetics might result from KiSS-34’s truncated nature, and slower dissociation is hypothesized to be due to improved KISS1R-binding from the 2-Nal moiety. In their SAR study, Tomita et al. observed a 4-fold higher potency of KiSS-34 (AMBA^5^-2-Nal^6^-Gly^7^-Leu^8^-Arg^9^-Trp^10^-NH_2_) compared to a Phe^6^-analogue, indicating a large binding pocket in KISS1R ideal for amino acids with large aromatic groups. Amide protons in the Trp^10^ residue are hypothesized to directly interact with KISS1R through hydrogen bonding, while changes in the remaining residues decrease the agonistic properties. In future studies, more potent agonists or antagonists might be possible through optimization of *N*-terminal 2-Nal^6^ and *C*-terminal Trp^10^ residues through natural or unnatural aromatic amino acids.^35^

[^177^Lu]­Lu-DOTA-KiSS-34 and [^177^Lu]­Lu-DOTA-KP-10 were also examined in selected native cancer cells. The first uptake benchmarks of KP-based radioligands were recently published by Israel et al., who reported *in vitro* uptakes of 0.6–4.4% for their [^68^Ga]­Ga-NODAGA-KP-54 radioligand in different cancers and observed the highest values in LNCaP cells.^25^ In a comparative study, we observed similarly high total uptakes in LNCaP cells for [^177^Lu]­Lu-DOTA-KiSS-34 (3.4%) and [^177^Lu]­Lu-DOTA-KP-10 (6.8%) but notably low internalization rates (<5%) compared to transfected cells. Low uptakes in C33A and Hep3B2 cells indicate the unsuitability of these cell lines, likely resulting from endogenous KISS1R expression levels falling below the functional thresholds congruent to live-cell microscopy results. Blocking studies in LNCaP cells suggest a non-KISS1R specific binding mechanism, as excess KiSS-34 did not decrease total uptakes. This is in line with blocking studies performed by Israel et al.,^25^ who observed only a partial reduction of radioligand uptake in LNCaP models *in vitro* (by 50%) and *in vivo* (by 26 ± 5%). In their examined cell lines, a substantial radioligand uptake (40–50%) remained after blocking, indicating unknown mechanisms mandatory to be elucidated.

PET/CT imaging of [^68^Ga]­Ga-DOTA-KPs (KP-54, KP-10, KiSS-34) in healthy BALB/c mice revealed tissue distribution profiles in accordance to published data.^26^ However, postinjection urine analysis of KP-10 and KP-54 counterparts clearly reveals *in vivo* degradation congruent to studies in human serum, plasma, and whole blood.^26,29^ In contrast, [^68^Ga]­Ga-DOTA-KiSS-34 remains stable *in vivo*. Radioligand stability ensures that radionuclides are not released due to decomplexation or proteolytic degradation. [^68^Ga]­Ga-DOTA-KiSS-34 shows a substantially decreased kidney retention compared to KP-10 ^26^ and KP-54 ^25^ counterparts. Nevertheless, the liver accumulation for this tracer was higher due to lipophilic building blocks (AMBA, 2-Nal) and must be optimized to ensure targeted uptake of KISS1R-expressing cancers. Future optimizations aim to balance hepatic uptake through PEGylation, charged linkers (e.g., lysine, aspartic acid), or hydrophilic chelators (e.g., DOTAGA).^48^

In summary, our results indicate that lead structure optimization alone is insufficient; a comprehensive understanding of receptor trafficking is crucial to advancing KISS1R-targeted radiotheranostics. At this stage, our target detection, internalization, and imaging studies are primarily limited to transfected cells or healthy BALB/c mice. In future studies, our models will be validated in TNBC and further native cancer models, both *in vitro* and *in vivo*, including toxicity assessments at therapeutic doses. In addition, antagonistic KPs and coincubation of substrates known to stimulate KISS1R expression,^49^ e.g., tamoxifen, remain unexplored and could prove beneficial, to specifically target and investigate recycling pathways. Radioligands able to target both surface and intracellularly expressed receptors could offer another option, e.g., through cell-penetrating peptides or nanoparticles.^50^

## 5 Conclusions

In our study, we characterized critical challenges in KISS1R target detection, highlighting complex receptor dynamics for standard radiotheranostic approaches. By using a ligand-based approach, we were able to detect rapid internalization dynamics in both fluorescence-based imaging and *in vitro* internalization assays. Among examined radioligands, Ga-68- and Lu-177-labeled DOTA-KiSS-34 exhibited improved stability and favorable tissue distribution profiles compared with its KP-10 and KP-54 counterparts, positioning it as a promising lead structure for KISS1R-based radiotheranostics. Regardless, the interplay of KP and KISS1R needs to be further investigated before robust TNBC *in vivo* models can be established to take advantage of its full radiotheranostic potential.

## Supplementary Material



## Notes

S1 – Detailed synthesis protocols of all KP-based ligands with HPLC and ESI-MS analysis, S2.1 – Experimental details of FACS studies with lists of all examined cell lines, used Abs, and AF-488-KPs, S2.2 – Experimental details of IHC studies including used cell sections, tissues, Abs, and AF-488-KPs, S2.3 – KISS1R-specific peptide sequences used in KISS1R proteomics/MS, S2.4 – Live-cell imaging of AF-488-KP-10 and scrambled controls in CHO-KISS1R and native cancer cell lines, S3 – Detailed concentration-, time-, and volume-based internalization assays, S4 – Oil-based binding kinetics assay: control experiments in KISS1R-negative SKOV3 (PDF)

## ABBREVIATIONS

AGC: Automatic gain control
BALB: Bagg albino mice
BCA: Bicinchoninic acid assay
CAA: Chloroacetamide
CHO: Chinese hamster ovary
Cryo-EM: Cryogenic electron microscopy
CT: Computerized tomography
DCM: Dichloromethane
DIPEA: *N*,*N*-Diisopropylethylamine
DMEM: Dulbecco’s modified eagle medium
DMF: *N*,*N*-Dimethylformamide
DMSO: Dimethyl sulfoxide
DOTA: 1,4,7,10-Tetraazacyclododecane-*N*,*N*′,*N*,*N*′-tetraacetic acid
DOTA-TATE: DOTA-(Tyr3)-octreotate
ESI: Electrospray ionization
FCS: Fetal calf serum
FITC: Fluorescein isothiocyanate
HBTU: Hexafluorophosphate benzotriazole tetramethyl uronium
HCD: Higher-energy collision energy dissociation
HEPES: 4-(2-Hydroxyethyl)-1-piperazineethanesulfonic acid
HPLC: High-performance liquid chromatography
*m*/*z*: Mass-to-charge ratio
MS: Mass spectrometry
MW: Molecular weight
NCE: Normalized collision energy
NHS: *N*-Hydroxysuccinimide
NODAGA: (1,4,7-Triazacyclononane-1,4,7-triacetic acid) + GA (glutaric acid)
PBS: Phosphate-buffered saline
PTFE: Polytetrafluoroethylene
RP-HPLC: Reversed-phase high-performance liquid chromatography
RT: Room temperature
RT-PCR: Reverse transcription polymerase chain reaction
SDS: Sodium dodecyl sulfate
siRNA: Small interfering RNA
*t*Bu: *tert*-Butyl
TCEP: Tris­(2-carboxyethyl)­phosphine
TEAB: Triethylammonium bicarbonate
TIC: Total ion current
TLC: Thin-layer chromatography
WT: Wild type
