# Supplementary material for: Investigation of Radiolabeled KISS1R Ligands as Promising Tools for Diagnosis and Treatment of Triple-Negative Breast Cancer
Source: Mol Pharm. 2026 Mar 3;23(4):2720–34. doi: 10.1021/acs.molpharmaceut.5c01853 (PMC13058897; doi:10.1021/acs.molpharmaceut.5c01853)
Supplement: Supplementary file 1 [file mp5c01853_si_001.pdf]

Supporting Information

Investigation of Radiolabeled KISS1R Ligands as Promising Tools for Diagnosis and Treatment of Triple-Negative Breast Cancer

Harun Taş[1,*], Martin Schäfer[2], Aneeba Shuja-Uddin[1], Ulrike Bauder-Wüst[1], Luciana Kovacs Dos Santos[1], Lisa Bartnitzky[3], Felix Oden[3], Magdalena Platzk[4], Tim König[4], Patrick Leopold Rüther[4], Elisabeth Pook[4], Kateřina Dvořáková Bendová[5], Zbyněk Nový[5,6], Miloš Petřík[5,6,7], Urs B. Hagemann[3], Martina Benešová-Schäfer[1,*]

1 Research Group Translational Radiotheranostics, German Cancer Research Center (DKFZ), Im Neuenheimer Feld 280, 69120 Heidelberg, Germany

2 Service Unit for Radiopharmaceuticals and Preclinical Studies, German Cancer Research Center (DKFZ), Im Neuenheimer Feld 280, 69120 Heidelberg, Germany

3 Bayer AG, 13342 Berlin, Germany

4 Bayer AG, 42113 Wuppertal, Germany

5 Institute of Molecular and Translational Medicine, Faculty of Medicine and Dentistry, Palacký University, 77900 Olomouc, Czech Republic

6 Czech Advanced Technology and Research Institute, Palacký University, 77900 Olomouc, Czech Republic

7 Institute of Molecular and Translational Medicine, University Hospital Olomouc, 77900 Olomouc, Czech Republic

* Correspondence:

Dr. Harun Taş

German Cancer Research Center (DKFZ)

Foundation under Public Law

Im Neuenheimer Feld 280

69120 Heidelberg Germany

harun.tas@dkfz-heidelberg.de;

Phone: +49-6221-42-5359

Dr. Martina Benešová-Schäfer

German Cancer Research Center (DKFZ)

Foundation under Public Law

Im Neuenheimer Feld 280

69120 Heidelberg Germany

m.benesova@dkfz-heidelberg.de

Phone: +49-6221-42-5355

Fax: +49-6221-42-5356

Contents

SI1 – Peptide Synthesis and Analytical Characterization....4
1. Solid-Phase Peptide Syntheses (SPPS) of KPs, DOTA and AF-488 conjugates....4
1.1 KPs....6
KP-10 – "Metastin 45-54"....7
KP-10-EEE....11
KiSS-34....15
KiSS-34-EEE....19
1.2 DOTA-KPs....23
DOTA-KP-10....24
DOTA-KP-10-EEE....28
DOTA-KiSS-34....32
DOTA-KiSS-34-EEE....36
1.3 AF-488-KPs....40
AF-488-KP-10....41
AF-488-KP-10-EEE....45
AF-488-KiSS-34....49
1.4 Scrambled KPs and DOTA and AF-488 conjugates....52
KP-10s....53
AF-488-KP-10s....57
DOTA-KP-10s....61
KiSS-34s....66
AF-488-KiSS-34s....70
DOTA-KiSS-34s....73
SI2 – Target Receptor Studies....77
1. FACS....77
2. IHC....82
3. Proteomics / MS....85
4. Live-cell imaging....87
SI3 – Internalization Assays....88
SI4 – Oil-based binding kinetics assay....90

SI1 – Peptide Synthesis and Analytical Characterization

1. Solid-Phase Peptide Syntheses (SPPS) of KPs, DOTA and AF-488 conjugates

All peptide syntheses, including scrambled controls, were conducted with an ABI 433A Peptide Synthesizer operated with the SynthAssist 3.1 software. The syntheses were conducted with the FastMoc 0.10 mmol program running on HBTU-mediated automated Fmoc protocol (amino acid (AA) (mmol) = 1.00, cycle time (min) = 24, waste per cycle (mL) = 50, Ratio of AA:Resin = 10:1) on 0.1 mmol of a rink amide resin (155 mg, loading: 0.645 mmol/g, 100–200 mesh). All KP-54-based (Metastin 54) constructs were obtained from BioCat GmbH (Heidelberg, Germany) in sufficient purity.

After automated synthesis, the peptide-functionalized resin was washed with 6 x 5 mL Et_2O and dried *in vacuo*. Subsequent cleavage from the resin was performed with a mixture of 3 mL TFA, 75 µL distilled H_2O and 75 µL Triisopropylsilane (TIPS) for 4 hours. The cleaved off peptide was precipitated in 50 mL Et_2O and centrifuged prior to HPLC purification.

The conjugation of the DOTA chelator was realized using HBTU-activated DOTA-*tris*(*t*Bu)ester on the KP-carrying resin. A syringe with 0.1 mmol of resin was loaded with a solution of 148 mg (3.92 eq., 0.392 mmol) HBTU, 115 mg (2 eq., 0.2 mmol) DOTA-*tris*(*t*Bu)ester and 300 µL (1.7 mmol) DIPEA in 3 mL DMF and kept on a rotary mixer overnight. The cleavage and work-up was conducted in analogy to the aforementioned procedure.

For semi-preparative HPLC a LATEK P-402 pump coupled to Merck Hitachi L-7420 (UV/VIS signal) was employed. Peptides and corresponding DOTA conjugates were purified using an Orbit 100 C18 RP-HPLC column (MZ Analysentechnik; 5µm, 100 Å, 250x30mm, MZ0901-2503000) with either of the following gradients, unless stated otherwise:

Eluents:
i) (A) Water + 0.1% TFA, (B) ACN + 0.1% TFA; Gradient: 0–40 min 5–95% B; Flow: 30 mL/min; Wavelength: 214 nm; Temperature: RT. ii) (A) Water + 0.1% TFA, (B) ACN + 0.1% TFA; Gradient: 0–40 min 5–60% B; Flow: 30 mL/min; Wavelength: 214 nm; Temperature: RT.

AF-488-KPs were synthesized by addition of a solution containing 5 mg (0.06 mmol) AF-488 NHS ester and 10 µL (0.056 mmol) DIPEA in 200 µL DMF or DMSO to 0.01 mmol of free KP in 150 µL DMF or DMSO and subsequent stirring at room temperature overnight. Afterwards, the reaction solution was taken up in 6–10 mL of ACN:H_2O (1:1, *v*/*v*), sonicated in a warm water bath, if necessary to improve the solubility, and purified in analogy.
AF-488-KPs were purified using a NUCLEODUR HILIC HPLC-column (Macherey Nagel; 5 µm, 110 Å, 250x21 mm) with the following gradient, unless stated otherwise:

Eluents:
(A) Water + 0.2% Formic Acid (FA), (B) ACN + 0.2% FA; Gradient: 0–40 min 97–60% B; Flow: 15 mL/min; Wavelength: 214 nm; Temperature: RT.

Analytical HPLC was conducted using by a Thermo Fisher Ultimate 3000 HPLC with a variable wavelength detector using a Phenomenex Aeris Peptide 3.8u XB-C18 LC-column (Phenomenex; 3.6 µm, 100 Å, 150 × 4.60 mm) with the following gradient, unless noted otherwise:

Eluents: i) (A) Water + 0.1% TFA, (B) ACN + 0.1% TFA; Gradient B: 0–17 min 5–95%, 17–22 min, 24 min 5%; Flow: 0.8 mL/min; Injection volume: 0.5 µL, Wavelength: 214 nm, Temperature: RT. ii) (A) Water + 0.1% TFA, (B) ACN + 0.1% TFA; Gradient B: 0–12 min 5–95%, 12–15 min 95%, 15–18 min 95–5%, 18–20 min 5%; Flow: 0.8 mL/min; Injection volume: 0.5 µL, Wavelength: 254 nm, Temperature: RT.

AF-488-KiSS-34 and its scrambled analog were analyzed with an Agilent 1100 Series using a Phenomenex Aeris Peptide 3.8u XB-C18 LC-column (Phenomenex; 3.6µm, 100 Å, 150x4.60mm) with the following gradient: Eluents: (A) Water + 0.1% TFA, (B) ACN + 0.1% TFA; Gradient: 0–20 min 5–70% B, 20–25 min 70–100%; Flow: 0.8 mL/min; Injection volume: 0.5 µL, Wavelength: 214 (A), 254 (B), 220 (C) nm, Ref: 360 nm; Temperature: RT.

MS spectra were acquired by Bruker Esquire 600 equipped with an ion trap and LT2 Plus (Scientific Analysis Instruments, SAI) equipped with a time-of-flight (TOF) detector.

1.1 KPs

Table S1. List of synthesized KPs.

Substance Name	Structure	Mol. Weight [g/mol]	Yield [%]
KP-54 (Metastin 54)	Gly-Thr-Ser-Leu-Ser-Pro-Pro-Pro-Glu-Ser-Ser-Gly-Ser-Arg-Gln-Gln-Pro-Gly-Leu-Ser-Ala-Pro-His-Ser-Arg-Gln-Ile-Pro-Ala-Pro-Gln-Gly-Ala-Val-Leu-Val-Gln-Arg-Glu-Lys-Asp-Leu-Pro-Asn-Tyr-Asn-Trp-Asn-Ser-Phe-Gly-Leu-Arg-Phe-NH_2	5857.5	--*
KP-10 (Metastin 45-54)	Tyr-Asn-Trp-Asn-Ser-Phe-Gly-Leu-Arg-Phe-NH_2	1302.4	53
KP-10-EEE	Glu-Glu-Glu-Tyr-Asn-Trp-Asn-Ser-Phe-Gly-Leu-Arg-Phe-NH_2	1689.8	49
KiSS-34	AMBA-2-Nal-Gly-Leu-Arg-Trp-NH_2	860.0	21
KiSS-34-EEE$^{(\gamma)}$	(γ)Glu-Glu-Glu-AMBA-2-Nal-Gly-Leu-Arg-Trp-NH_2	1247.4	20

*commercially purchased

KP-10 – “Metastin 45-54”

HPLC chromatogram (RP):

(A) Water + 0.1% Trifluoroacetic acid (TFA), (B) ACN + 0.1% TFA; Gradient: 0–40 min 5–95% B; Flow: 30 mL/min; Wavelength: 214 nm; Temperature: RT.

Retention time: 18.35 min

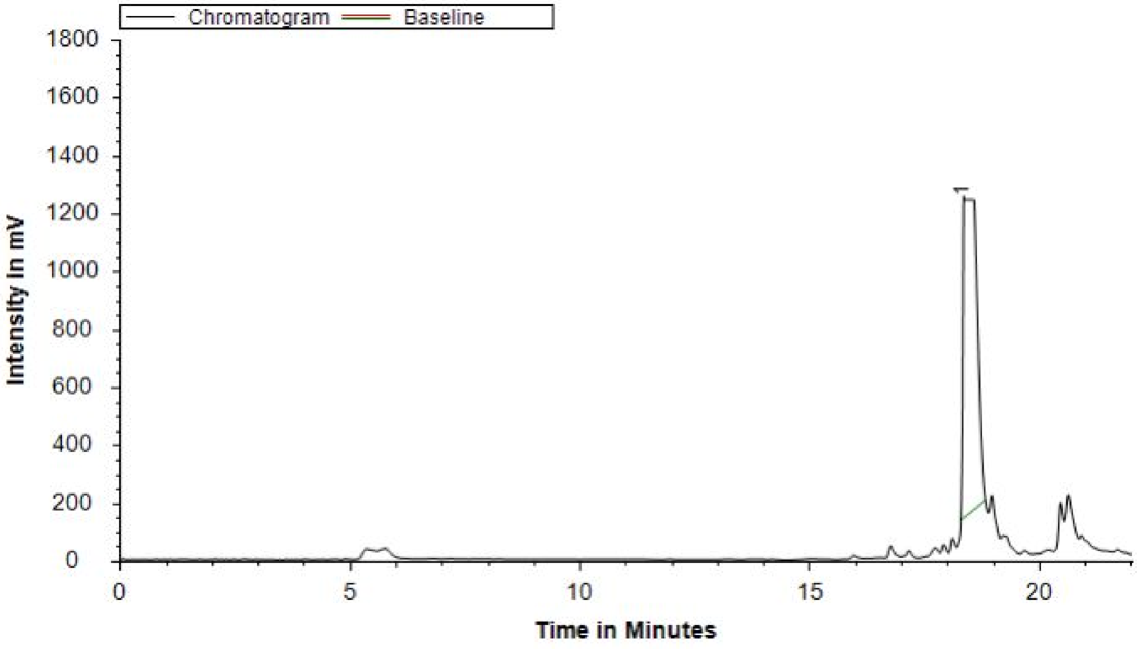

Result Table

No.	Ret. Time	Height	Area	Percent	Name
1	18.35	1096.698	2.260064E+07	100	

ESI-MS:

Target mass: 1302.4 g/mol

Detected mass: 1302.4 $[M]^+$, 651.6 $[M/2]^+$

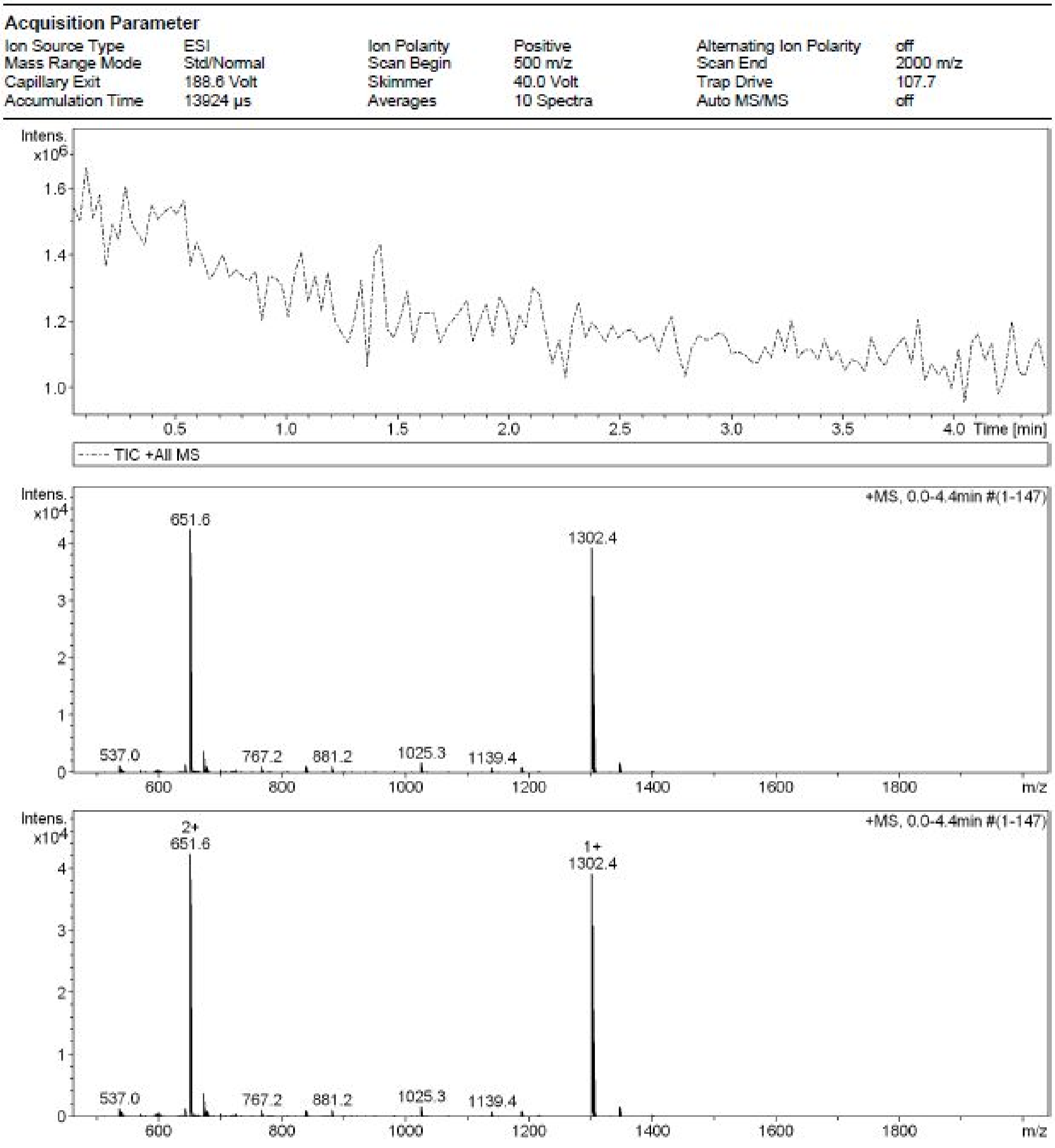

Analytical HPLC:

214 nm

Chromatogram and Results

Injection Details

Injection Name:	**Kp-10 214nm**	*Run Time (min):*	**24,00**
Vial Number:	**RB3**	*Injection Volume:*	**10,00**
Injection Type:	**Unknown**	*Channel:*	**UV_VIS_1**
Calibration Level:		*Wavelength:*	**214**
Instrument Method:	**5_95 in 17min 0,8ml 214nm Auto**	*Bandwidth:*	**n.a.**
Processing Method:	**Basic Quantitative**	*Dilution Factor:*	**1,0000**
Injection Date/Time:	**30.Jun.25 15:39**	*Sample Weight:*	**1,0000**

Chromatogram

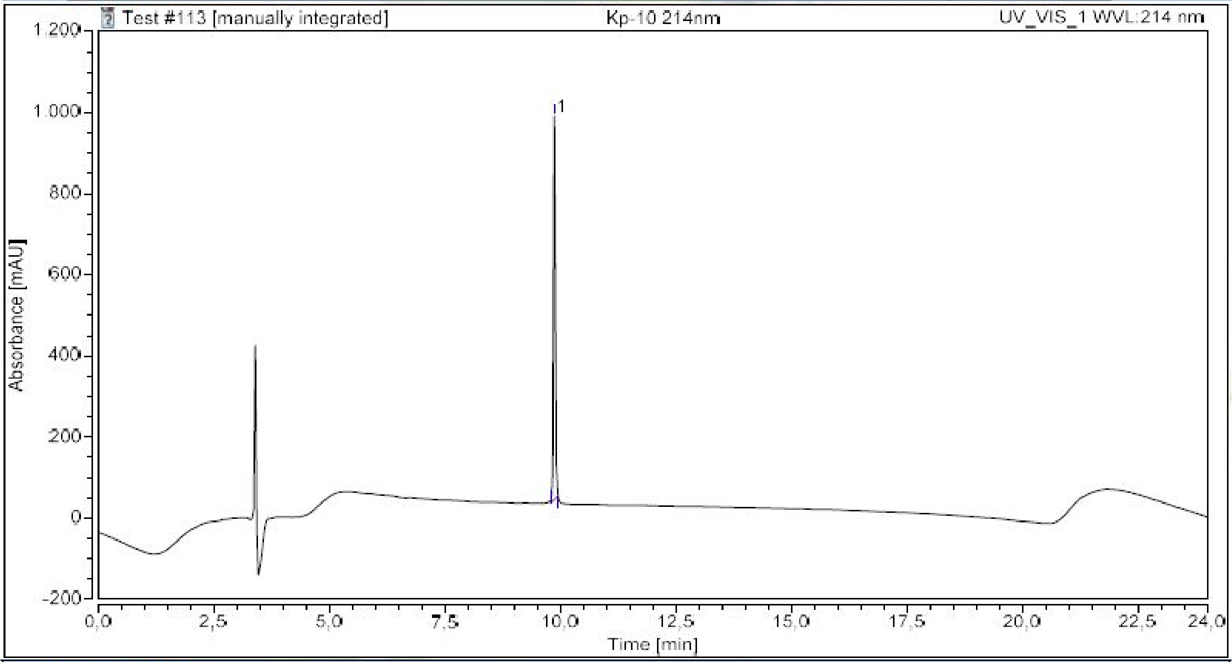

Integration Results

No.	Peak Name	Retention Time min	Area mAU*min	Height mAU	Relative Area %	Relative Height %	Amount n.a.
1	n.a.	9,865	38,210	946,003	100,00	100,00	n.a.
**Total:**			**38,210**	**946,003**	**100,00**	**100,00**	

254 nm

Chromatogram and Results

Injection Details

Injection Name:	**Kp-10 254nm**	*Run Time (min):*	**20,00**
Vial Number:	**RB3**	*Injection Volume:*	**10,00**
Injection Type:	**Unknown**	*Channel:*	**UV_VIS_1**
Calibration Level:		*Wavelength:*	**254**
Instrument Method:	**5_95 in 17min 0,8ml 254nm Auto**	*Bandwidth:*	**n.a.**
Processing Method:	**Basic Quantitative**	*Dilution Factor:*	**1,0000**
Injection Date/Time:	**30.Jun.25 11:30**	*Sample Weight:*	**1,0000**

Chromatogram

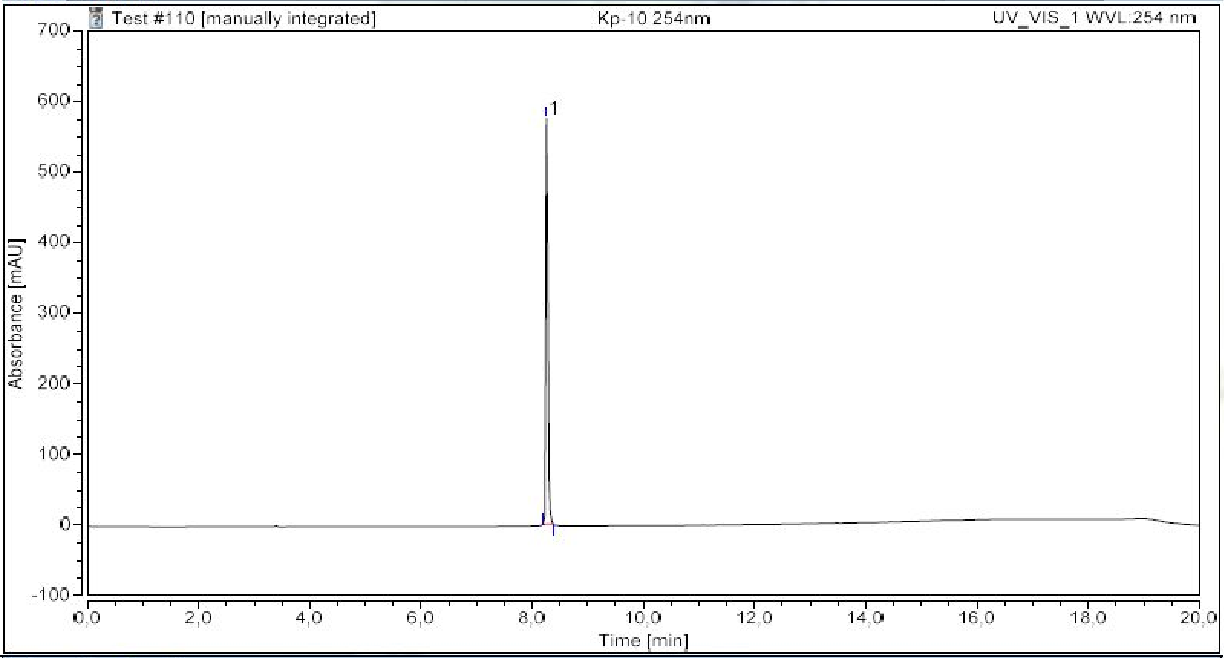

Integration Results

No.	Peak Name	Retention Time min	Area mAU*min	Height mAU	Relative Area %	Relative Height %	Amount n.a.
1	n.a.	8,260	25,685	575,443	100,00	100,00	n.a.
**Total:**			**25,685**	**575,443**	**100,00**	**100,00**	

KP-10-EEE

HPLC chromatogram (RP):

(A) Water + 0.1% Trifluoroacetic acid (TFA), (B) ACN + 0.1% TFA; Gradient: 0–40 min 5–95% B; Flow: 30 mL/min; Wavelength: 214 nm; Temperature: RT.

Retention time: 17.72 min

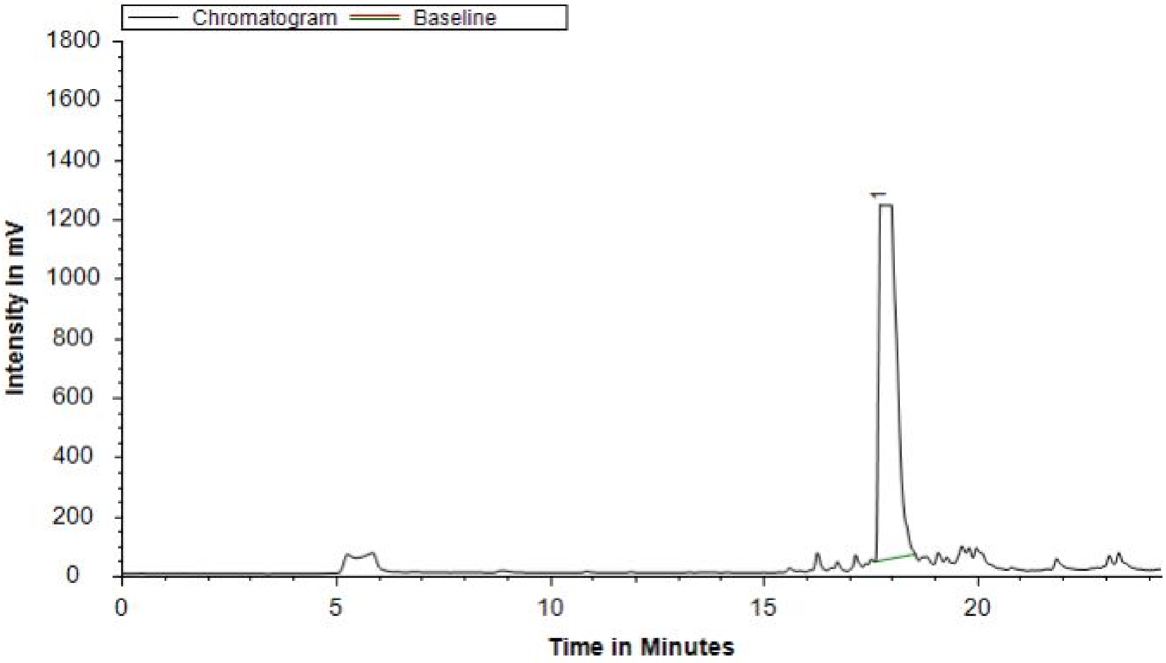

Result Table

No.	Ret. Time	Height	Area	Percent	Name
1	17.71667	1194.645	3.405332E+07	100	

ESI-MS:

Target mass: 1689.8 g/mol

Detected mass: 1689.4 $[M]^+$, 845.1 $[M/2]^+$

Acquisition Parameter

Ion Source Type	ESI	Ion Polarity	Positive	Alternating Ion Polarity	off
Mass Range Mode	Std/Normal	Scan Begin	200 m/z	Scan End	1800 m/z
Capillary Exit	184.5 Volt	Skimmer	40.0 Volt	Trap Drive	104.1
Accumulation Time	94 µs	Averages	20 Spectra	Auto MS/MS	off

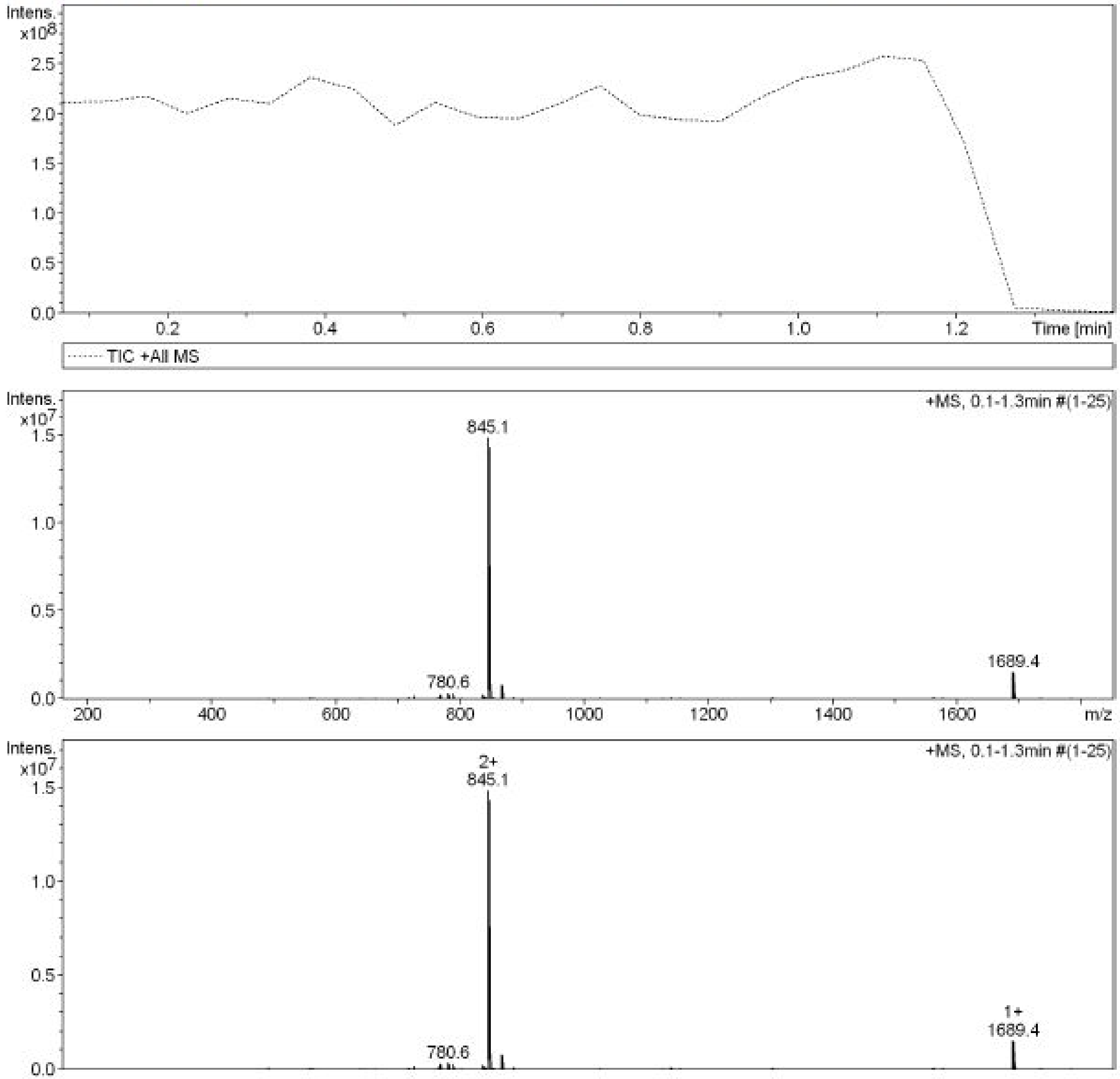

Analytical HPLC:

214 nm

Chromatogram and Results

Injection Details

Injection Name:	**Kp-10-EEE 214nm**	*Run Time (min):*	**24,00**
Vial Number:	**RB2**	*Injection Volume:*	**10,00**
Injection Type:	**Unknown**	*Channel:*	**UV_VIS_1**
Calibration Level:		*Wavelength:*	**214**
Instrument Method:	**5_95 in 17min 0,8ml 214nm Auto**	*Bandwidth:*	**n.a.**
Processing Method:	**Basic Quantitative**	*Dilution Factor:*	**1,0000**
Injection Date/Time:	**30.Jun.25 15:14**	*Sample Weight:*	**1,0000**

Chromatogram

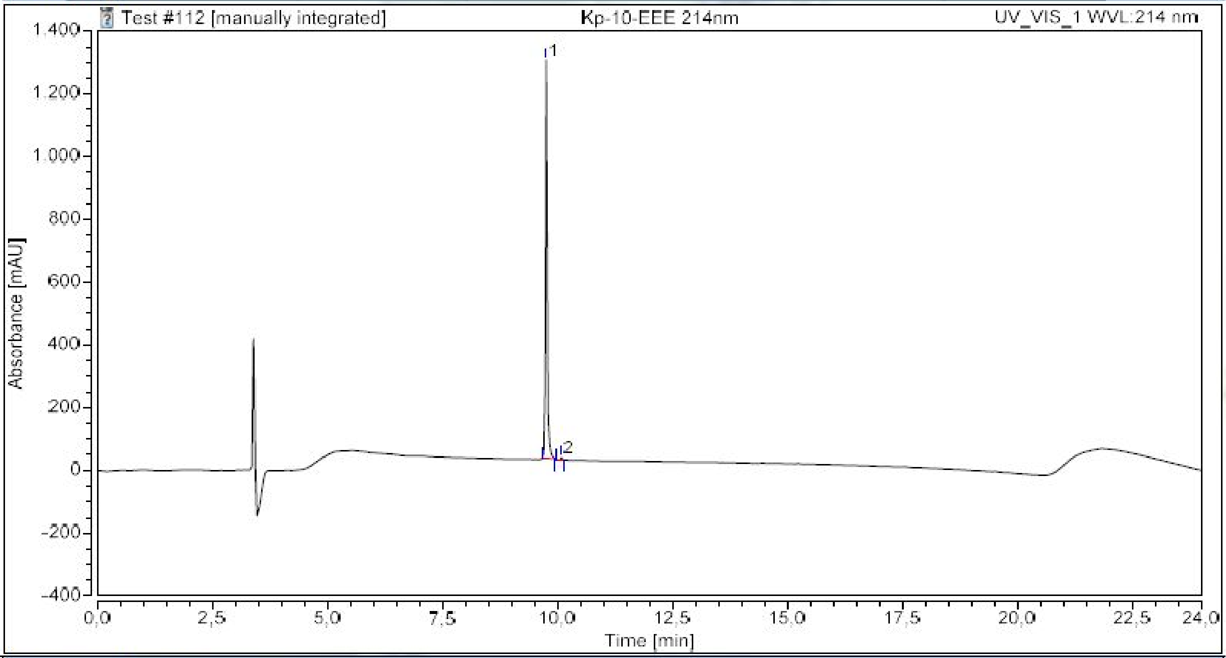

Integration Results

No.	Peak Name	Retention Time min	Area mAU*min	Height mAU	Relative Area %	Relative Height %	Amount n.a.
1	n.a.	9,755	56,951	1270,563	99,50	99,36	n.a.
2	n.a.	10,077	0,287	8,142	0,50	0,64	n.a.
Total:			**57,239**	**1278,705**	**100,00**	**100,00**	

254 nm

Chromatogram and Results

Injection Details

Injection Name:	**Kp-10-EEE 254nm**	*Run Time (min):*	**20,00**
Vial Number:	**RB2**	*Injection Volume:*	**10,00**
Injection Type:	**Unknown**	*Channel:*	**UV_VIS_1**
Calibration Level:		*Wavelength:*	**254**
Instrument Method:	**5_95 in 17min 0,8ml 254nm Auto**	*Bandwidth:*	**n.a.**
Processing Method:	**Basic Quantitative**	*Dilution Factor:*	**1,0000**
Injection Date/Time:	**30.Jun.25 11:09**	*Sample Weight:*	**1,0000**

Chromatogram

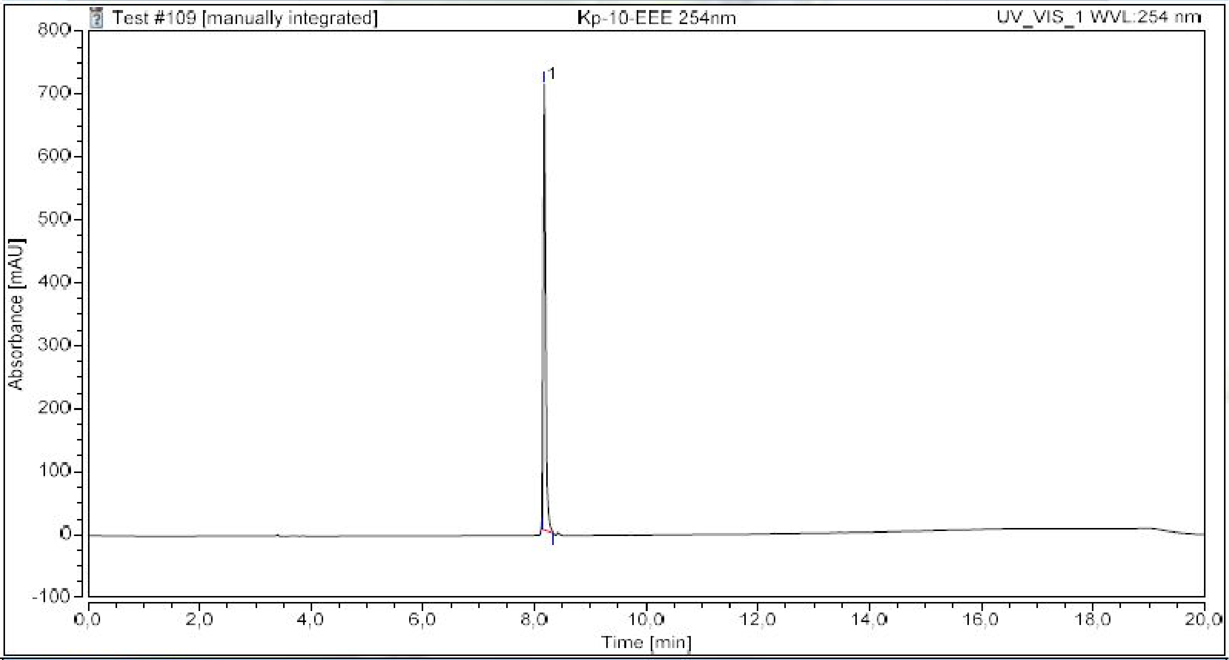

Integration Results

No.	Peak Name	Retention Time min	Area mAU*min	Height mAU	Relative Area %	Relative Height %	Amount n.a.
1	n.a.	8,172	33,022	709,165	100,00	100,00	n.a.
**Total:**			**33,022**	**709,165**	**100,00**	**100,00**	

KiSS-34

HPLC chromatogram (RP):

(A) Water + 0.1% Trifluoroacetic acid (TFA), (B) ACN + 0.1% TFA; Gradient: 0–40 min 5–95% B; Flow: 30 mL/min; Wavelength: 214 nm; Temperature: RT.

Retention time: 19.09 min

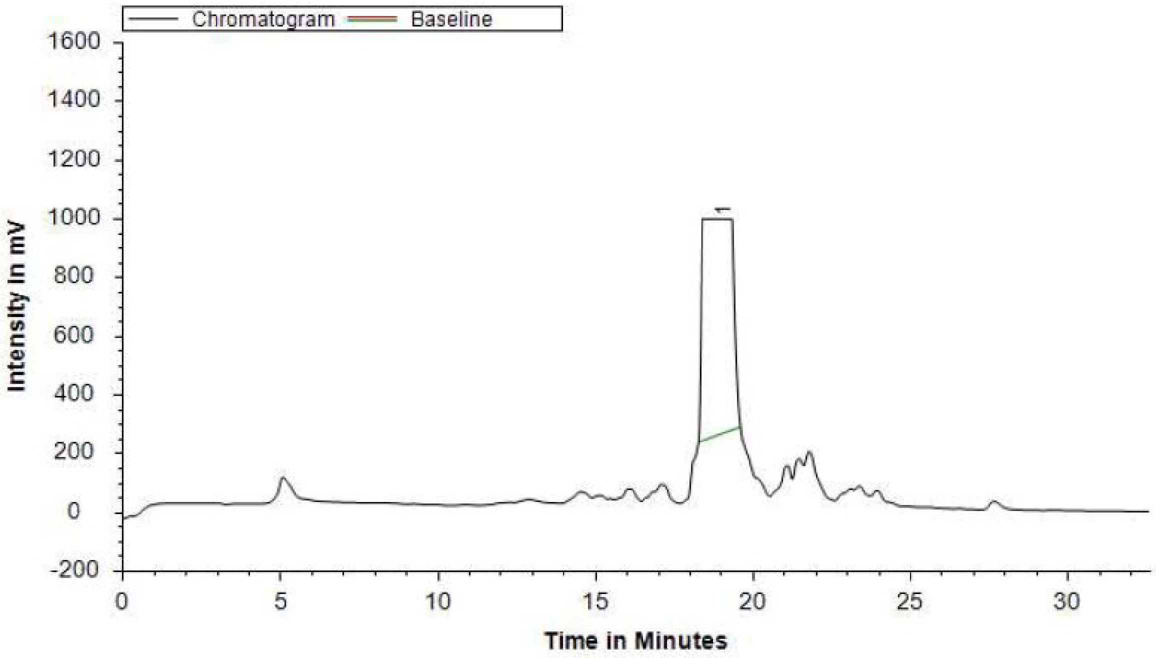

Result Table

No.	Ret. Time	Height	Area	Percent	Name
1	19.09167	727.1816	4.840681E+07	100	

ESI-MS:

Target mass: 860.0 g/mol

Detected mass: 860.2 $[M]^{+}$, 430.6 $[M/2]^{+}$

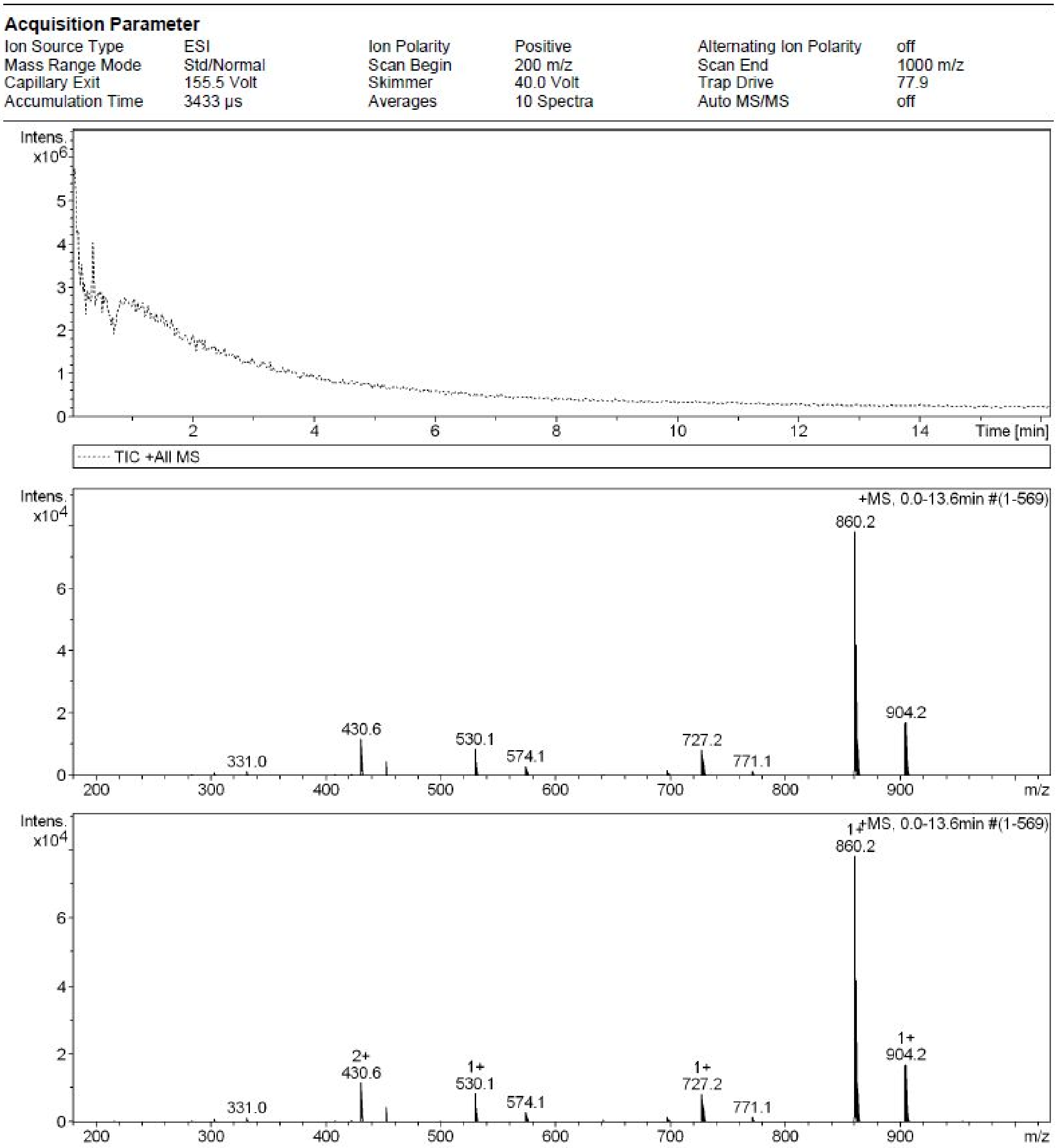

Analytical HPLC:

214 nm

Chromatogram and Results

Injection Details

Injection Name:	**K34 214nm**	*Run Time (min):*	**24,00**
Vial Number:	**RC1**	*Injection Volume:*	**10,00**
Injection Type:	**Unknown**	*Channel:*	**UV_VIS_1**
Calibration Level:		*Wavelength:*	**214**
Instrument Method:	**5_95 in 17min 0,8ml 214nm Auto**	*Bandwidth:*	**n.a.**
Processing Method:	**Basic Quantitative**	*Dilution Factor:*	**1,0000**
Injection Date/Time:	**25.Jul.25 15:50**	*Sample Weight:*	**1,0000**

Chromatogram

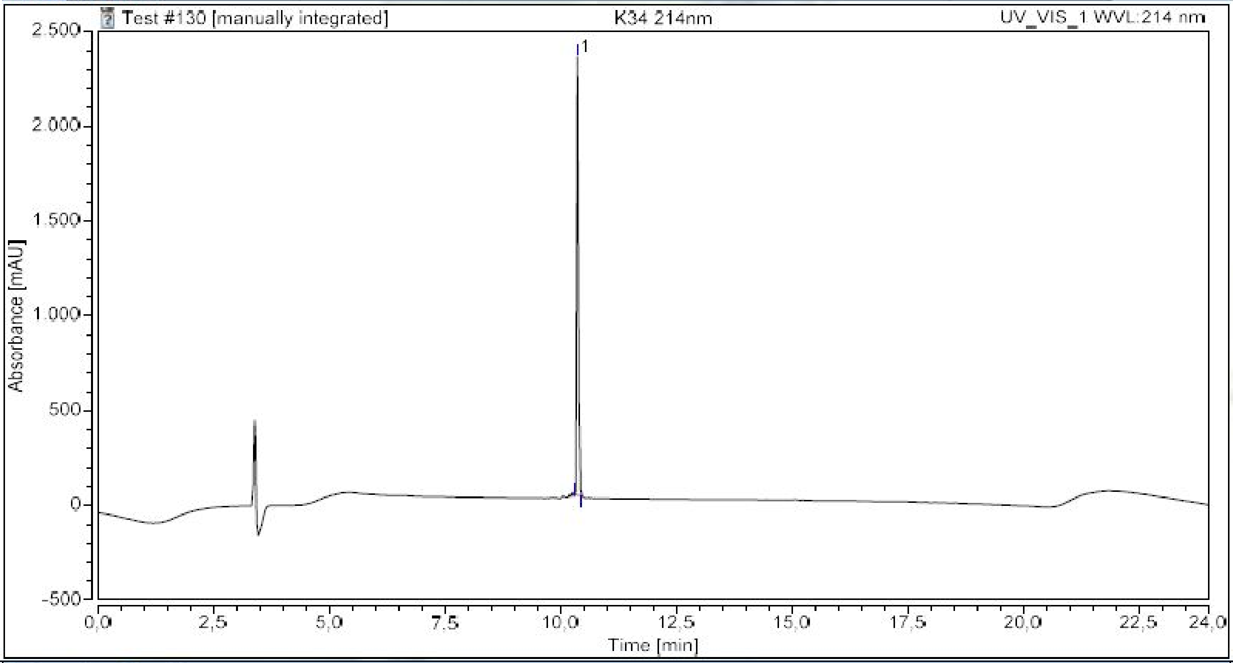

Integration Results

No.	Peak Name	Retention Time min	Area mAU*min	Height mAU	Relative Area %	Relative Height %	Amount n.a.
1	n.a.	10,367	95,378	2314,182	100,00	100,00	n.a.
Total:			**95,378**	**2314,182**	**100,00**	**100,00**	

254 nm

Chromatogram and Results

Injection Details

Injection Name:	**K34 254nm**	*Run Time (min):*	**20,00**
Vial Number:	**RC1**	*Injection Volume:*	**10,00**
Injection Type:	**Unknown**	*Channel:*	**UV_VIS_1**
Calibration Level:		*Wavelength:*	**254**
Instrument Method:	**5_95 in 17min 0,8ml 254nm Auto**	*Bandwidth:*	**n.a.**
Processing Method:	**Basic Quantitative**	*Dilution Factor:*	**1,0000**
Injection Date/Time:	**25.Jul.25 17:05**	*Sample Weight:*	**1,0000**

Chromatogram

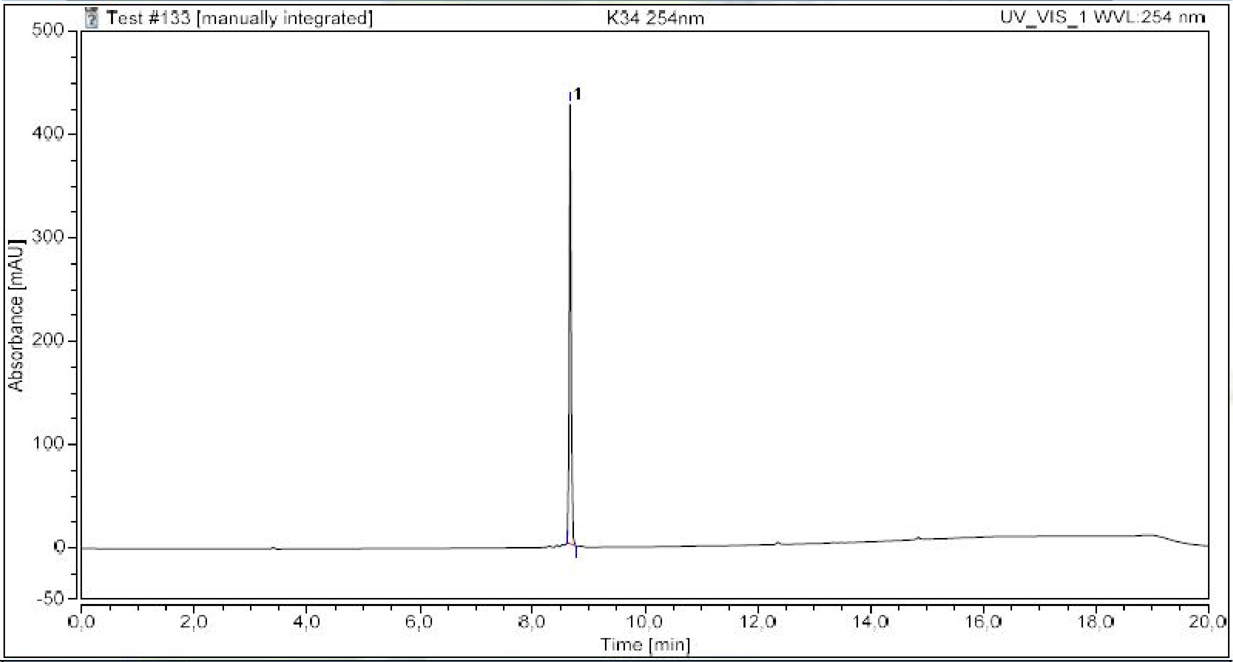

Integration Results

No.	Peak Name	Retention Time min	Area mAU*min	Height mAU	Relative Area %	Relative Height %	Amount n.a.
1	n.a.	8,667	17,207	425,738	100,00	100,00	n.a.
**Total:**			**17,207**	**425,738**	**100,00**	**100,00**	

KiSS-34-EEE

HPLC chromatogram (RP):

(A) Water + 0.1% Trifluoroacetic acid (TFA), (B) ACN + 0.1% TFA; Gradient: 0–40 min 10–95% B; Flow: 30 mL/min; Wavelength: 214 nm; Temperature: RT.

Retention time: 16.89 min

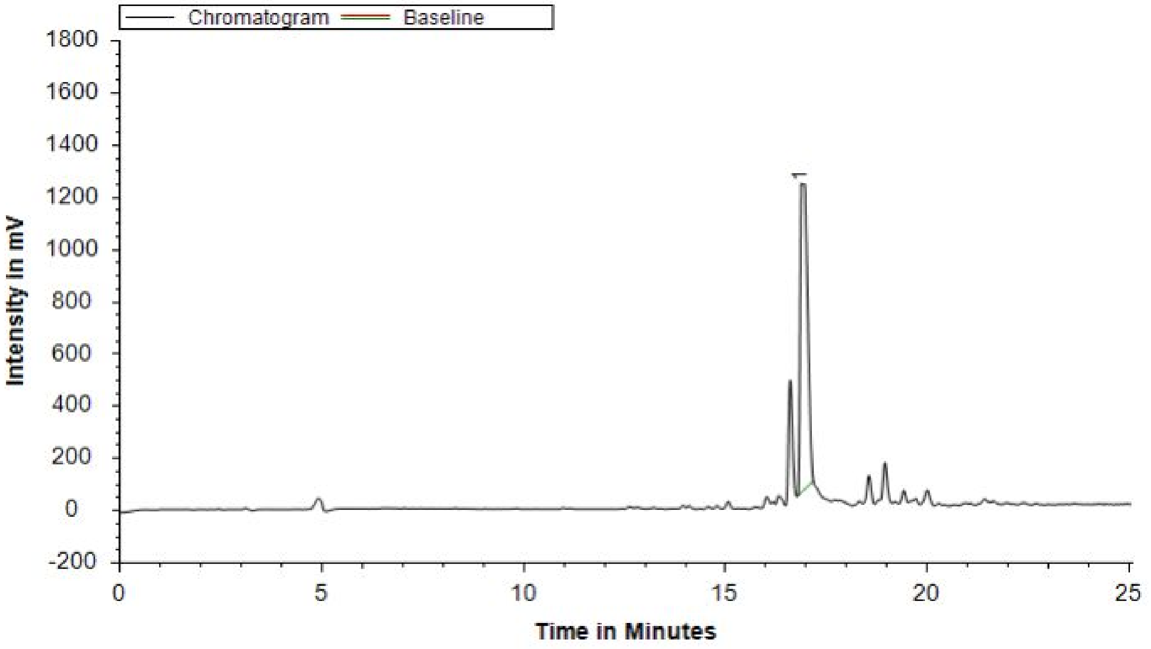

Result Table

No.	Ret. Time	Height	Area	Percent	Name
1	16.88333	1178.495	1.477041E+07	100	

ESI-MS:

Target mass: 1247.4 g/mol

Detected mass: 1247.3 $[M]^{+}$, 624.1 $[M/2]^{+}$

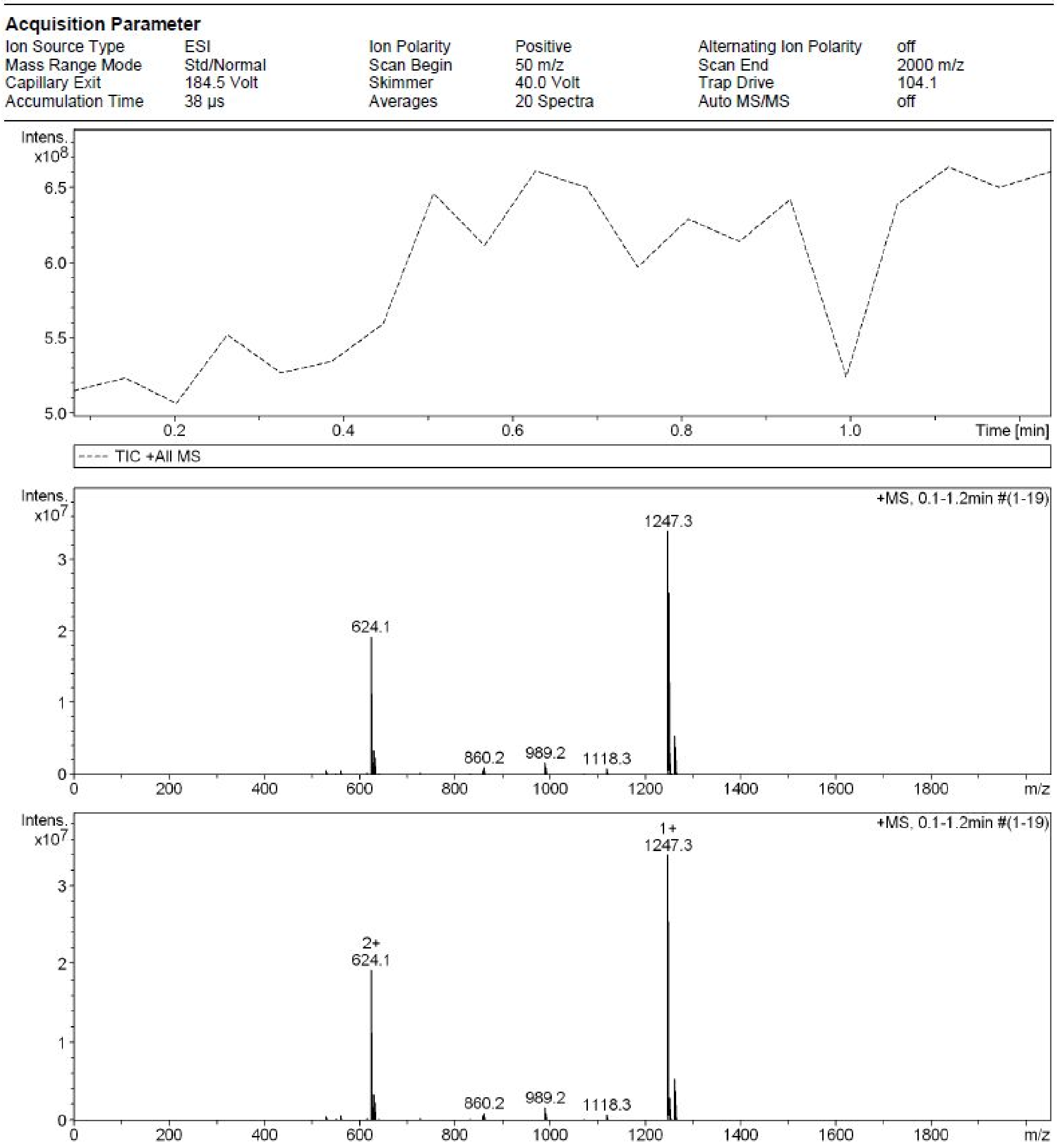

Analytical HPLC:

214 nm

Chromatogram and Results

Injection Details

Injection Name:	**K34EEE 214nm**	*Run Time (min):*	**24,00**
Vial Number:	**RC2**	*Injection Volume:*	**10,00**
Injection Type:	**Unknown**	*Channel:*	**UV_VIS_1**
Calibration Level:		*Wavelength:*	**214**
Instrument Method:	**5_95 in 17min 0,8ml 214nm Auto**	*Bandwidth:*	**n.a.**
Processing Method:	**Basic Quantitative**	*Dilution Factor:*	**1,0000**
Injection Date/Time:	**14.Jul.25 11:48**	*Sample Weight:*	**1,0000**

Chromatogram

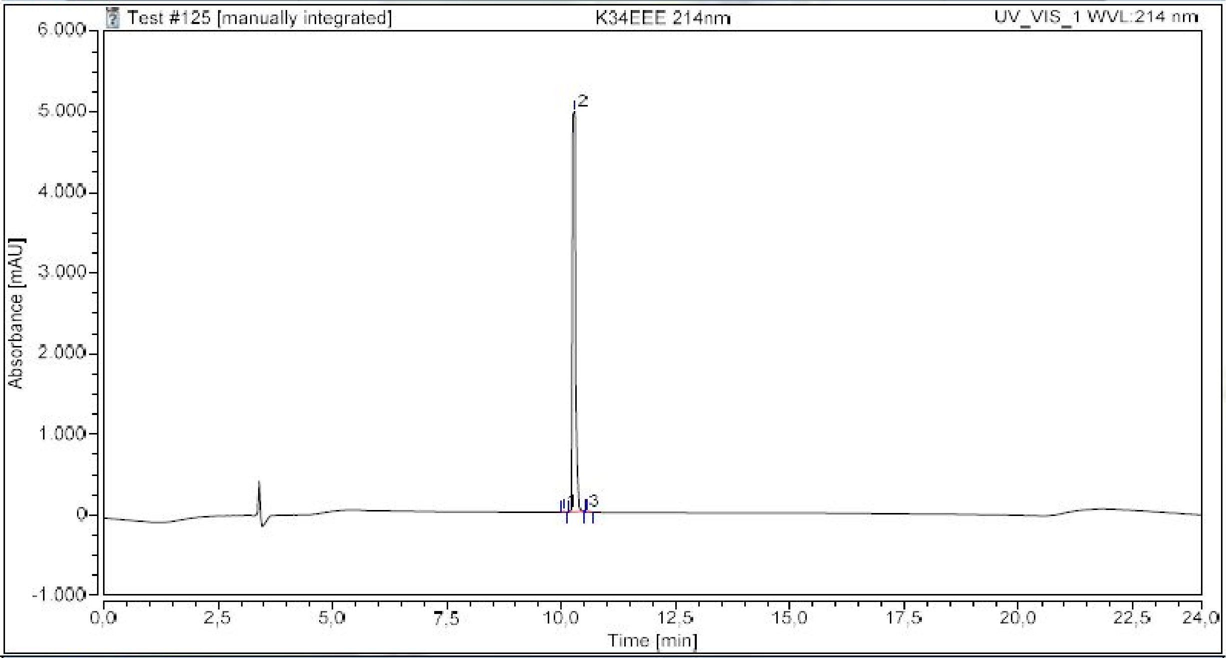

Integration Results

No.	Peak Name	Retention Time min	Area mAU*min	Height mAU	Relative Area %	Relative Height %	Amount n.a.
1	n.a.	10,055	0,506	13,161	0,13	0,26	n.a.
2	n.a.	10,305	379,848	4958,207	99,79	99,67	n.a.
3	n.a.	10,565	0,293	3,502	0,08	0,07	n.a.
Total:			380,647	4974,870	100,00	100,00	

254 nm

Chromatogram and Results

Injection Details

Injection Name:	**K34EEE 254nm**	*Run Time (min):*	**20,00**
Vial Number:	**RC2**	*Injection Volume:*	**10,00**
Injection Type:	**Unknown**	*Channel:*	**UV_VIS_1**
Calibration Level:		*Wavelength:*	**254**
Instrument Method:	**5_95 in 17min 0,8ml 254nm Auto**	*Bandwidth:*	**n.a.**
Processing Method:	**Basic Quantitative**	*Dilution Factor:*	**1,0000**
Injection Date/Time:	**14.Jul.25 10:49**	*Sample Weight:*	**1,0000**

Chromatogram

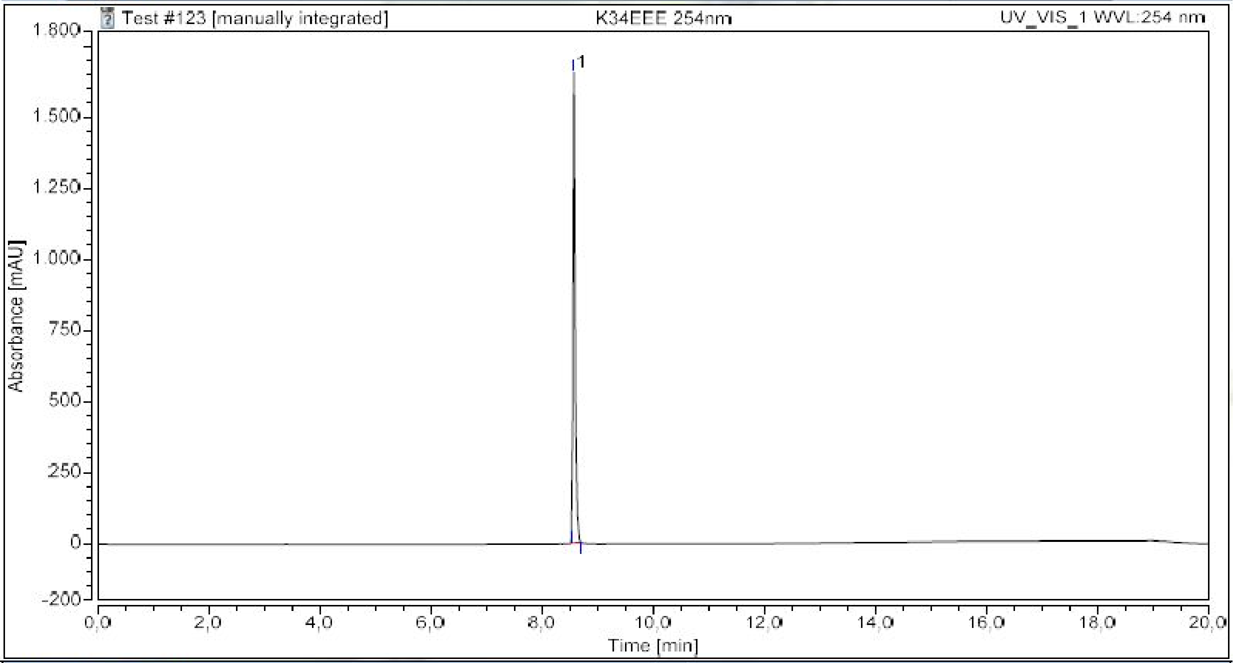

Integration Results

No.	Peak Name	Retention Time min	Area mAU*min	Height mAU	Relative Area %	Relative Height %	Amount n.a.
1	n.a.	8,572	75,272	1653,540	100,00	100,00	n.a.
**Total:**			**75,272**	**1653,540**	**100,00**	**100,00**	

1.2 DOTA-KPs

Table S2. List of synthesized DOTA-KPs.

Substance Name	Structure	Mol. Weight [g/mol]	Yield [%]
DOTA-KP-54	DOTA-Gly-Thr-Ser-Leu-Ser-Pro-Pro-Pro-Glu-Ser-Ser-Gly-Ser-Arg-Gln-Gln-Pro-Gly-Leu-Ser-Ala-Pro-His-Ser-Arg-Gln-Ile-Pro-Ala-Pro-Gln-Gly-Ala-Val-Leu-Val-Gln-Arg-Glu-Lys-Asp-Leu-Pro-Asn-Tyr-Asn-Trp-Asn-Ser-Phe-Gly-Leu-Arg-Phe-NH_2	6243.8	--*
DOTA-KP-10	DOTA-Tyr-Asn-Trp-Asn-Ser-Phe-Gly-Leu-Arg-Phe-NH_2	1688.8	41
DOTA-KP-10-EEE	DOTA-Glu-Glu-Glu-Tyr-Asn-Trp-Asn-Ser-Phe-Gly-Leu-Arg-Phe-NH_2	2076.2	32
DOTA-KiSS-34	DOTA-AMBA-2-Nal-Gly-Leu-Arg-Trp-NH_2	1246.4	10
DOTA-KiSS-34-EEE$^{(\gamma)}$	DOTA-(γ)Glu-Glu-Glu-AMBA-2-Nal-Gly-Leu-Arg-Trp-NH_2	1633.8	18

*commercially purchased

DOTA-KP-10

HPLC chromatogram (RP):

(A) Water + 0.1% Trifluoroacetic acid (TFA), (B) ACN + 0.1% TFA; Gradient: 0–40 min 5–60% B; Flow: 30 mL/min; Wavelength: 214 nm; Temperature: RT.

Retention time: 24.87 min

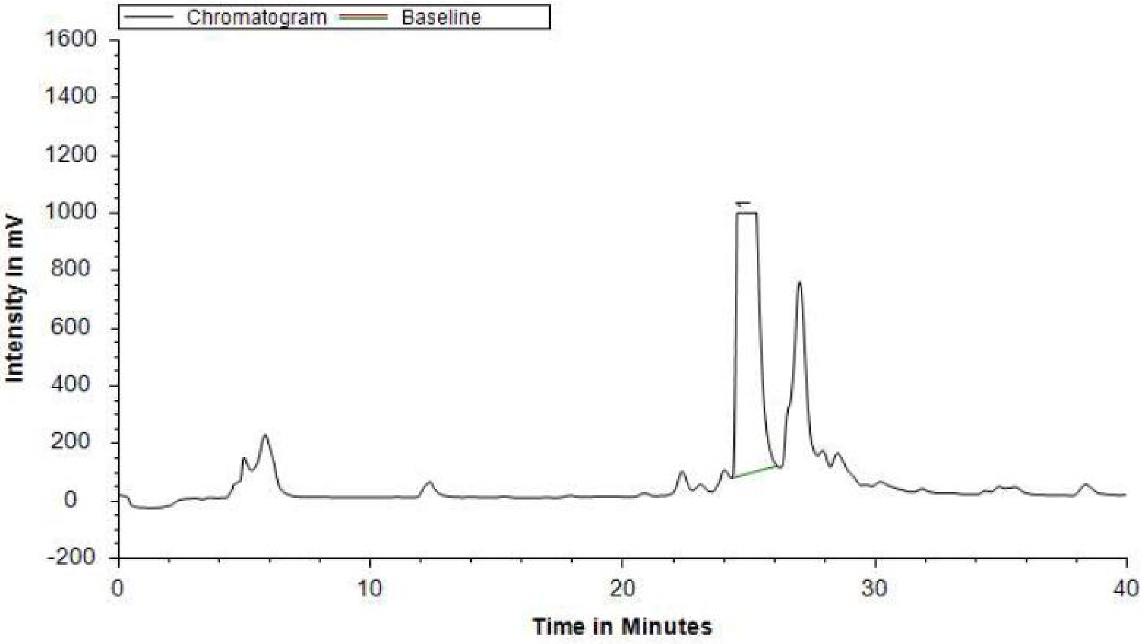

Result Table

No.	Ret. Time	Height	Area	Percent	Name
1	24.86667	906.7553	5.696295E+07	100	

ESI-MS:

Target mass: 1688.9 g/mol

Detected mass: 1689.5 $[M]^{+}$, 844.6 $[M/2]^{+}$

Acquisition Parameter

Ion Source Type	ESI	Ion Polarity	Positive	Alternating Ion Polarity	off
Mass Range Mode	Std/Normal	Scan Begin	100 m/z	Scan End	2000 m/z
Capillary Exit	217.5 Volt	Skimmer	40.0 Volt	Trap Drive	133.8
Accumulation Time	43 µs	Averages	20 Spectra	Auto MS/MS	off

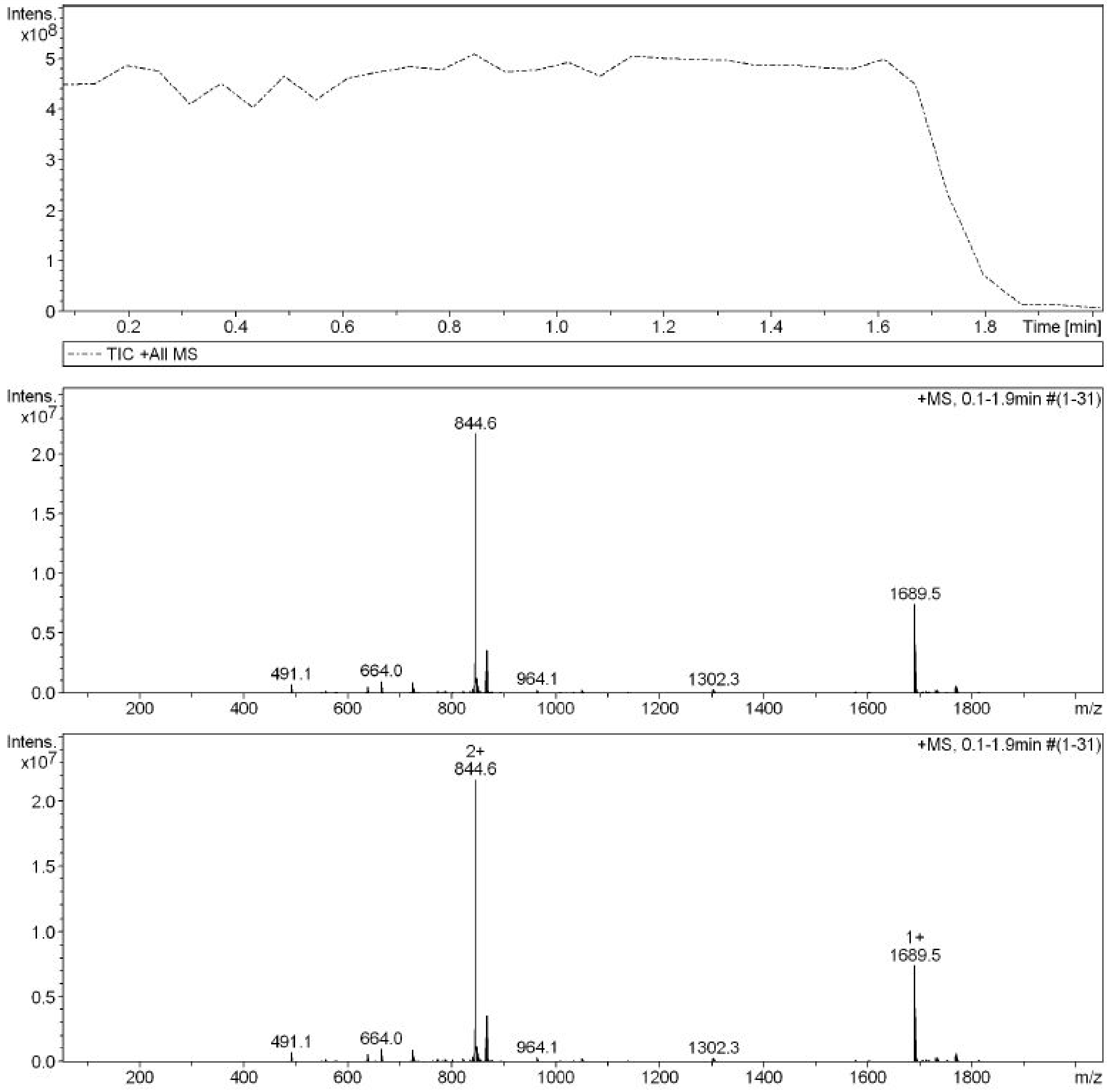

Analytical HPLC:

214 nm

Chromatogram and Results

Injection Details

Injection Name:	**DOTA-KP10 214nm**	*Run Time (min):*	**20,00**
Vial Number:	**RD1**	*Injection Volume:*	**10,00**
Injection Type:	**Unknown**	*Channel:*	**UV_VIS_1**
Calibration Level:		*Wavelength:*	**254**
Instrument Method:	**5_95 in 17min 0,8ml 254nm Auto**	*Bandwidth:*	**n.a.**
Processing Method:	**Basic Quantitative**	*Dilution Factor:*	**1,0000**
Injection Date/Time:	**16.Jul.25 18:15**	*Sample Weight:*	**1,0000**

Chromatogram

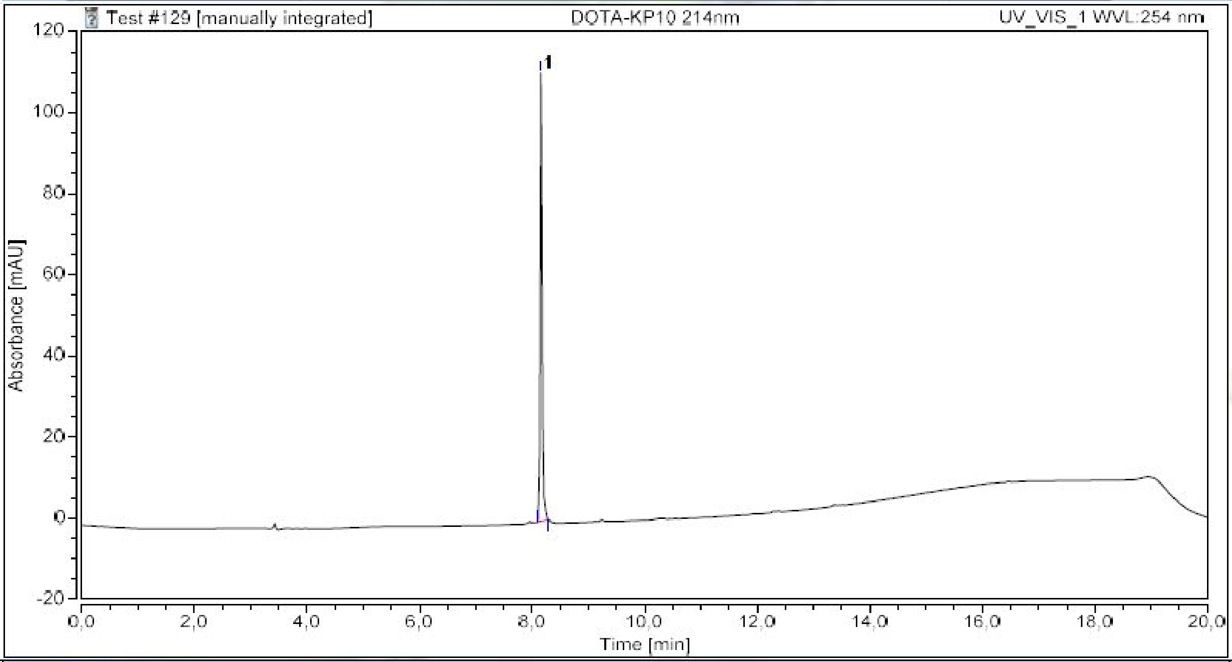

Integration Results

No.	Peak Name	Retention Time min	Area mAU*min	Height mAU	Relative Area %	Relative Height %	Amount n.a.
1	n.a.	8,158	4,660	110,549	100,00	100,00	n.a.
Total:			**4,660**	**110,549**	**100,00**	**100,00**	

254 nm

Chromatogram and Results

Injection Details

Injection Name:	**DOTA-KP10 254nm**	*Run Time (min):*	**20,00**
Vial Number:	**RC6**	*Injection Volume:*	**10,00**
Injection Type:	**Unknown**	*Channel:*	**UV_VIS_1**
Calibration Level:		*Wavelength:*	**254**
Instrument Method:	**5_95 in 17min 0,8ml 254nm Auto**	*Bandwidth:*	**n.a.**
Processing Method:	**Basic Quantitative**	*Dilution Factor:*	**1,0000**
Injection Date/Time:	**16.Jul.25 17:54**	*Sample Weight:*	**1,0000**

Chromatogram

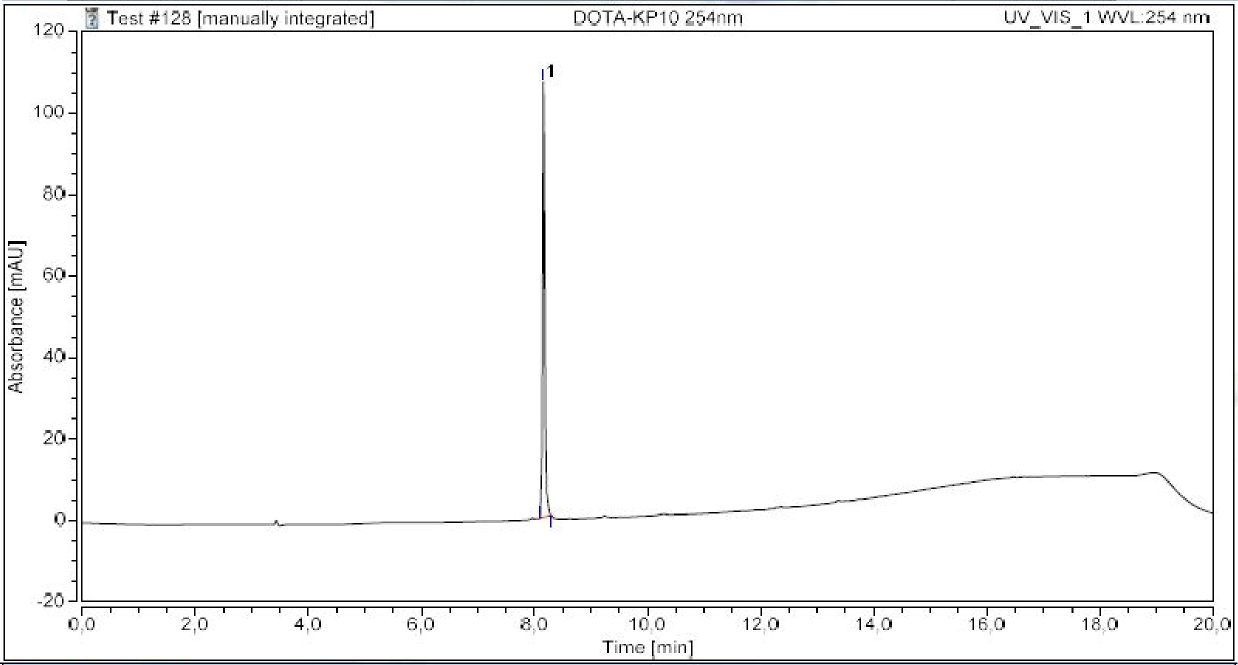

Integration Results

No.	Peak Name	Retention Time min	Area mAU*min	Height mAU	Relative Area %	Relative Height %	Amount n.a.
1	n.a.	8,158	4,495	106,913	100,00	100,00	n.a.
Total:			**4,495**	**106,913**	**100,00**	**100,00**	

DOTA-KP-10-EEE

HPLC chromatogram (RP):

(A) Water + 0.1% Trifluoroacetic acid (TFA), (B) ACN + 0.1% TFA; Gradient: 0–40 min 5–95% B; Flow: 30 mL/min; Wavelength: 214 nm; Temperature: RT.

Retention time: 18.35 min

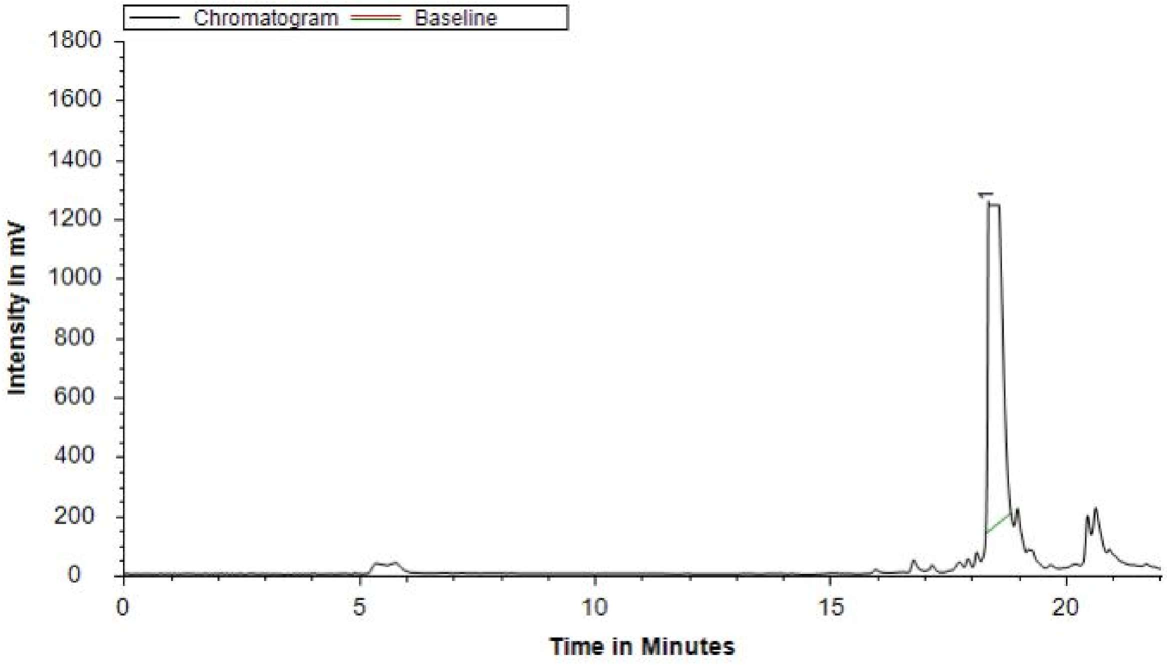

Result Table

No.	Ret. Time	Height	Area	Percent	Name
1	18.35	1096.698	2.260064E+07	100	

ESI-MS:

Target mass: 2076.2 g/mol

Detected mass: 2076.6 $[M]^{+}$, 1038.7 $[M/2]^{+}$

Acquisition Parameter

Ion Source Type	ESI	Ion Polarity	Positive	Alternating Ion Polarity	off
Mass Range Mode	Std/Normal	Scan Begin	400 m/z	Scan End	2200 m/z
Capillary Exit	246.7 Volt	Skimmer	40.0 Volt	Trap Drive	160.1
Accumulation Time	50 µs	Averages	20 Spectra	Auto MS/MS	off

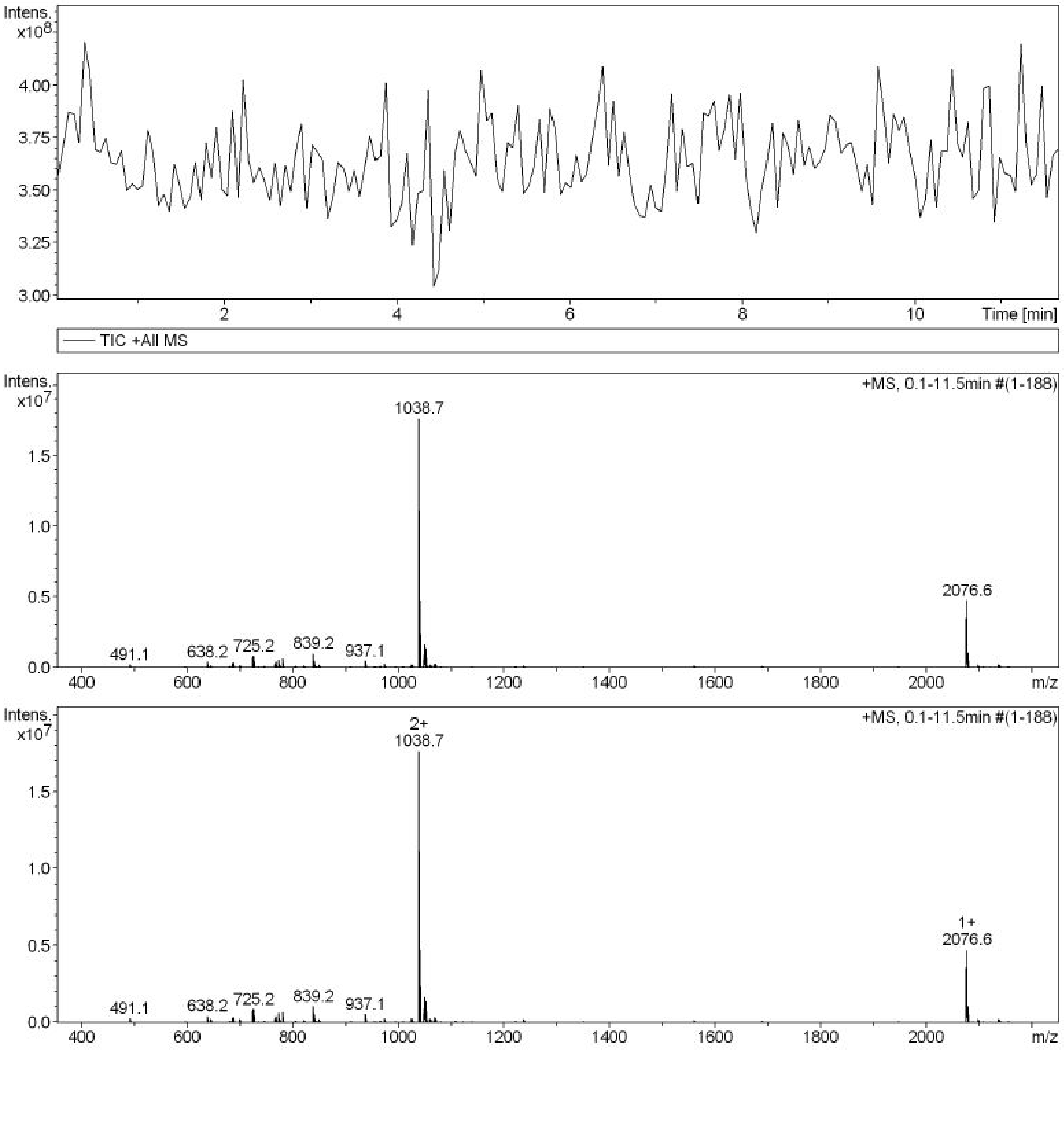

Analytical HPLC:

214 nm

Chromatogram and Results

Injection Details

Injection Name:	DOTA-KP10-EEE 214	*Run Time (min):*	24,00
Vial Number:	RD2	*Injection Volume:*	10,00
Injection Type:	Unknown	*Channel:*	UV_VIS_1
Calibration Level:		*Wavelength:*	214
Instrument Method:	5_95 in 17min 0,8ml 214nm Auto	*Bandwidth:*	n.a.
Processing Method:	Basic Quantitative	*Dilution Factor:*	1,0000
Injection Date/Time:	21.Jul.25 11:00	*Sample Weight:*	1,0000

Chromatogram

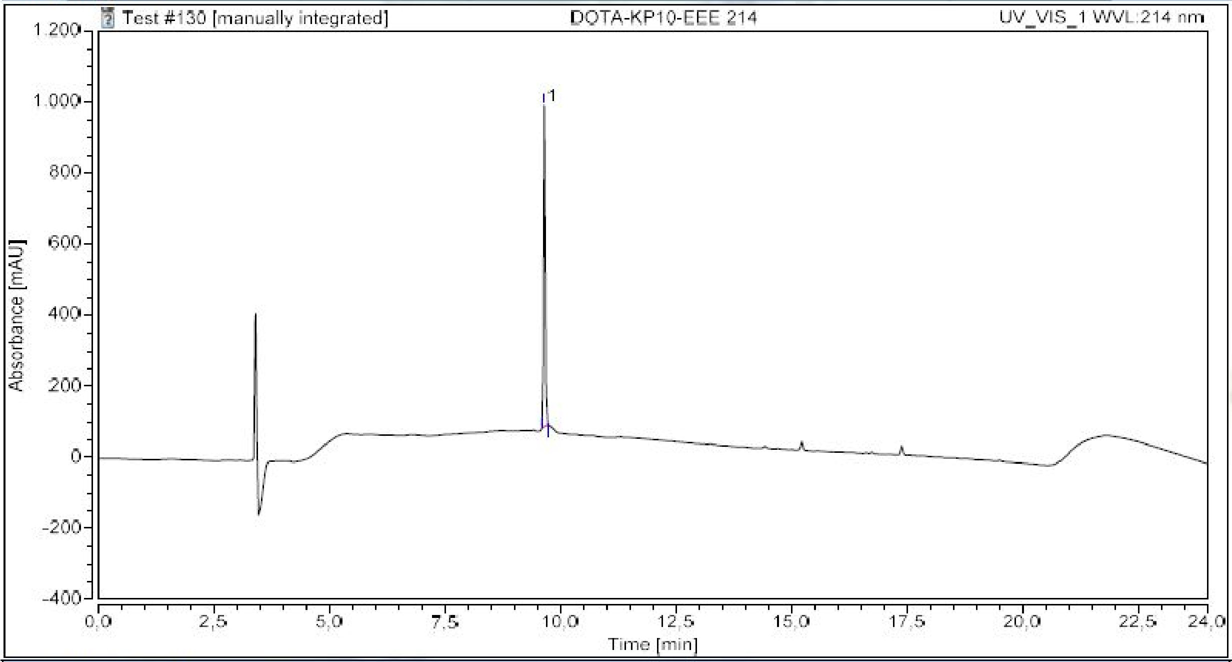

Integration Results

No.	Peak Name	Retention Time min	Area mAU*min	Height mAU	Relative Area %	Relative Height %	Amount n.a.
1	n.a.	9,650	38,364	904,734	100,00	100,00	n.a.
**Total:**			**38,364**	**904,734**	**100,00**	**100,00**	

254 nm

Chromatogram and Results

Injection Details

Injection Name:	**DOTA-KP10-EEE 254**	*Run Time (min):*	**20,00**
Vial Number:	**RD3**	*Injection Volume:*	**10,00**
Injection Type:	**Unknown**	*Channel:*	**UV_VIS_1**
Calibration Level:		*Wavelength:*	**254**
Instrument Method:	**5_95 in 17min 0,8ml 254nm Auto**	*Bandwidth:*	**n.a.**
Processing Method:	**Basic Quantitative**	*Dilution Factor:*	**1,0000**
Injection Date/Time:	**21.Jul.25 11:25**	*Sample Weight:*	**1,0000**

Chromatogram

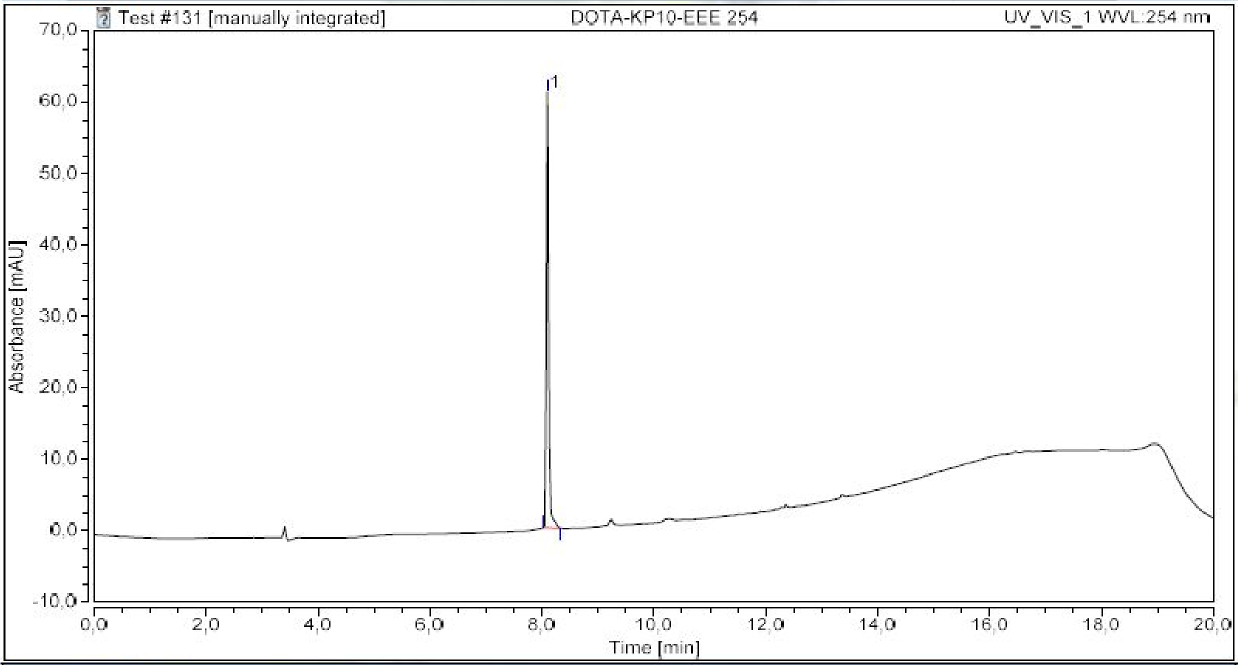

Integration Results

No.	Peak Name	Retention Time min	Area mAU*min	Height mAU	Relative Area %	Relative Height %	Amount n.a.
1	n.a.	8,095	2,640	61,087	100,00	100,00	n.a.
**Total:**			**2,640**	**61,087**	**100,00**	**100,00**	

DOTA-KiSS-34

HPLC Chromatogram (RP):

(A) Water + 0.1% Trifluoroacetic acid (TFA), (B) ACN + 0.1% TFA; Gradient: 0–40 min 5–60% B; Flow: 30 mL/min; Wavelength: 214 nm; Temperature: RT.

Retention time: 25.53 min

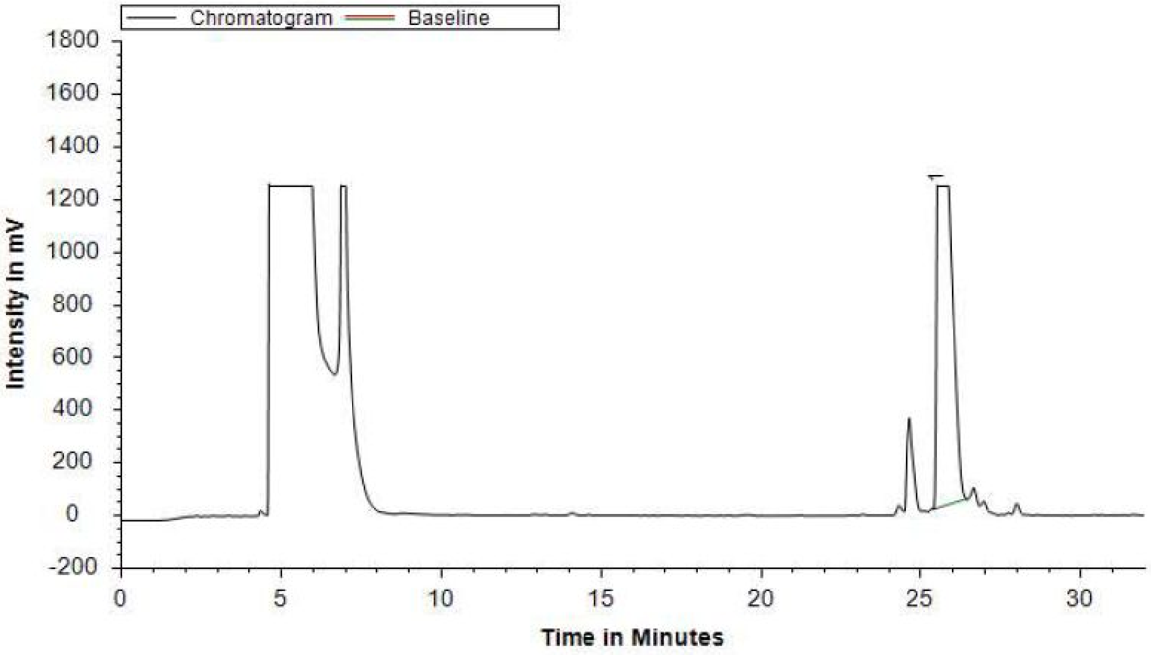

Result Table

No.	Ret. Time	Height	Area	Percent	Name
1	25.525	1219.76	4.311187E+07	100	

ESI-MS:

Target mass: 1246.4 g/mol

Detected mass: 1246.4 $[M]^+$, 623.6 $[M/2]^+$

Acquisition Parameter

Ion Source Type	ESI	Ion Polarity	Positive	Alternating Ion Polarity	off		
Mass Range Mode	Std/Normal	Scan Begin	100 m/z	Scan End	1500 m/z		
Capillary Exit	184.4 Volt	Skimmer	40.0 Volt	Trap Drive	104.0		
Accumulation Time	50 µs	Averages	20 Spectra	Auto MS/MS	off		

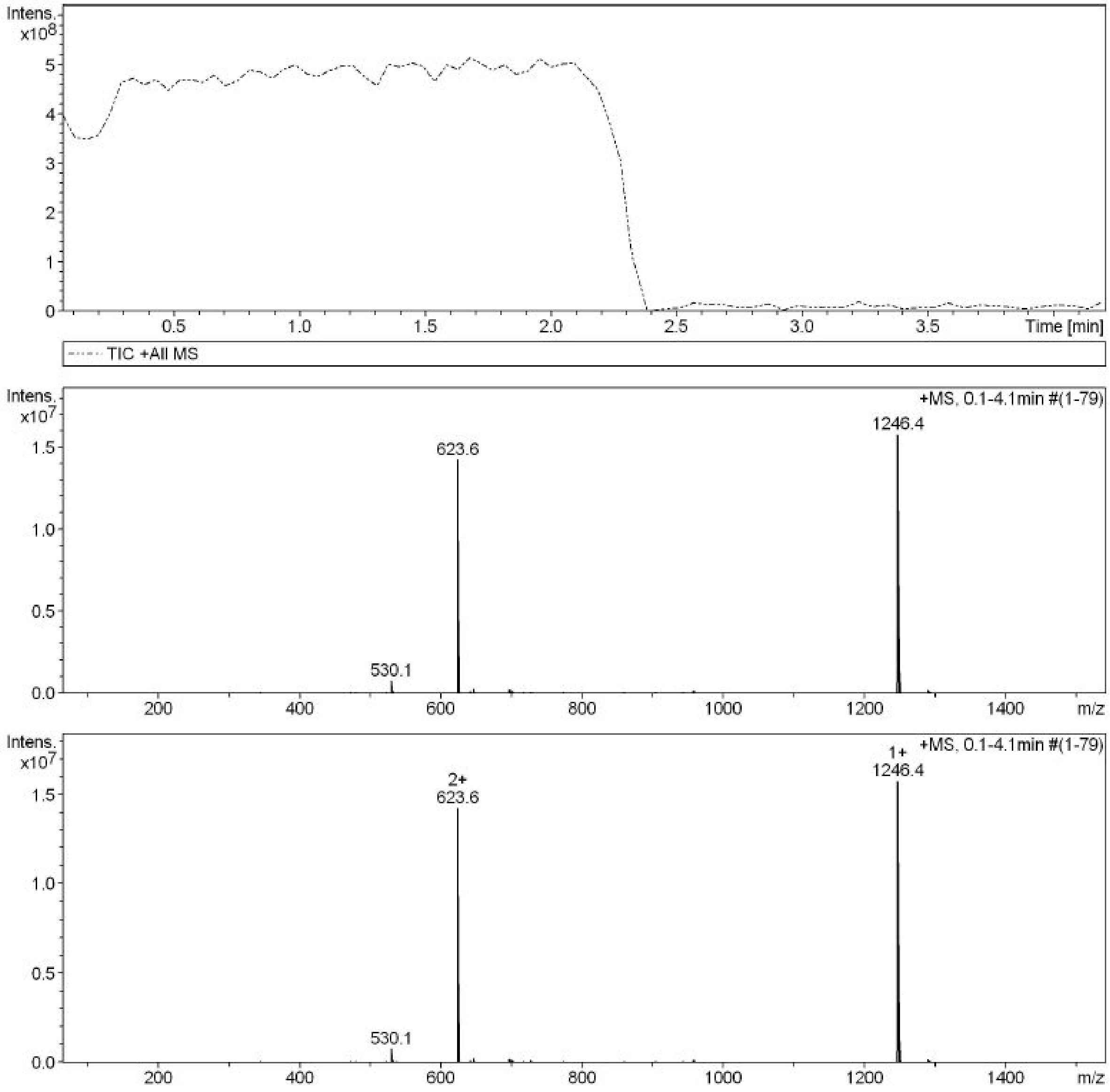

Analytical HPLC:

214 nm

Chromatogram and Results

Injection Details

Injection Name:	**DOTA-K34 214nm**	*Run Time (min):*	**24,00**
Vial Number:	**RC1**	*Injection Volume:*	**10,00**
Injection Type:	**Unknown**	*Channel:*	**UV_VIS_1**
Calibration Level:		*Wavelength:*	**214**
Instrument Method:	**5_95 in 17min 0,8ml 214nm Auto**	*Bandwidth:*	**n.a.**
Processing Method:	**Basic Quantitative**	*Dilution Factor:*	**1,0000**
Injection Date/Time:	**14.Jul.25 11:23**	*Sample Weight:*	**1,0000**

Chromatogram

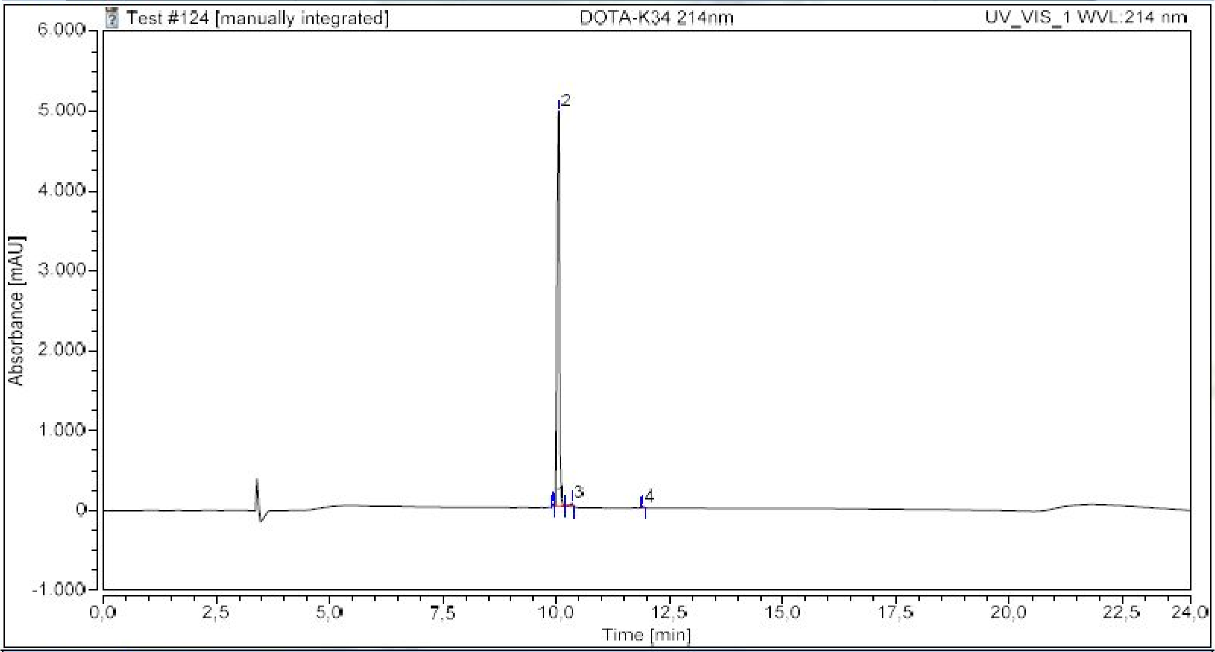

Integration Results

No.	Peak Name	Retention Time min	Area mAU*min	Height mAU	Relative Area %	Relative Height %	Amount n.a.
1	n.a.	9,935	1,496	42,190	0,48	0,84	n.a.
2	n.a.	10,060	303,494	4940,233	98,28	97,90	n.a.
3	n.a.	10,350	3,146	46,125	1,02	0,91	n.a.
4	n.a.	11,900	0,678	17,769	0,22	0,35	n.a.
**Total:**			**308,813**	**5046,317**	**100,00**	**100,00**	

254 nm

Chromatogram and Results

Injection Details

Injection Name:	**DOTA-K34 254nm**	*Run Time (min):*	**20,00**
Vial Number:	**RC1**	*Injection Volume:*	**10,00**
Injection Type:	**Unknown**	*Channel:*	**UV_VIS_1**
Calibration Level:		*Wavelength:*	**254**
Instrument Method:	**5_95 in 17min 0,8ml 254nm Auto**	*Bandwidth:*	**n.a.**
Processing Method:	**Basic Quantitative**	*Dilution Factor:*	**1,0000**
Injection Date/Time:	**14.Jul.25 10:28**	*Sample Weight:*	**1,0000**

Chromatogram

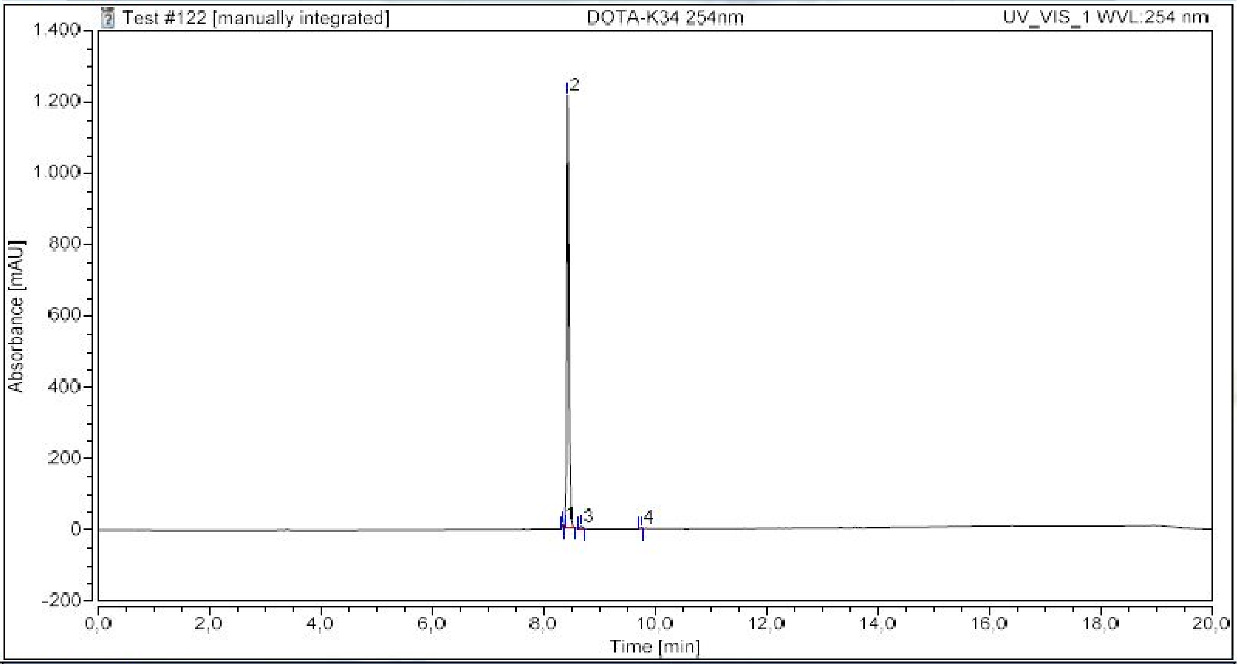

Integration Results

No.	Peak Name	Retention Time min	Area mAU*min	Height mAU	Relative Area %	Relative Height %	Amount n.a.
1	n.a.	8,347	0,349	10,643	0,69	0,86	n.a.
2	n.a.	8,428	49,835	1212,647	98,54	98,34	n.a.
3	n.a.	8,675	0,271	6,573	0,54	0,53	n.a.
4	n.a.	9,745	0,119	3,288	0,23	0,27	n.a.
**Total:**			**50,574**	**1233,151**	**100,00**	**100,00**	

DOTA-KiSS-34-EEE

HPLC Chromatogram (RP):

(A) Water + 0.1% Trifluoroacetic acid (TFA), (B) ACN + 0.1% TFA; Gradient: 0–40 min 5–60% B; Flow: 30 mL/min; Wavelength: 214 nm; Temperature: RT.

Retention time: 25.82 min

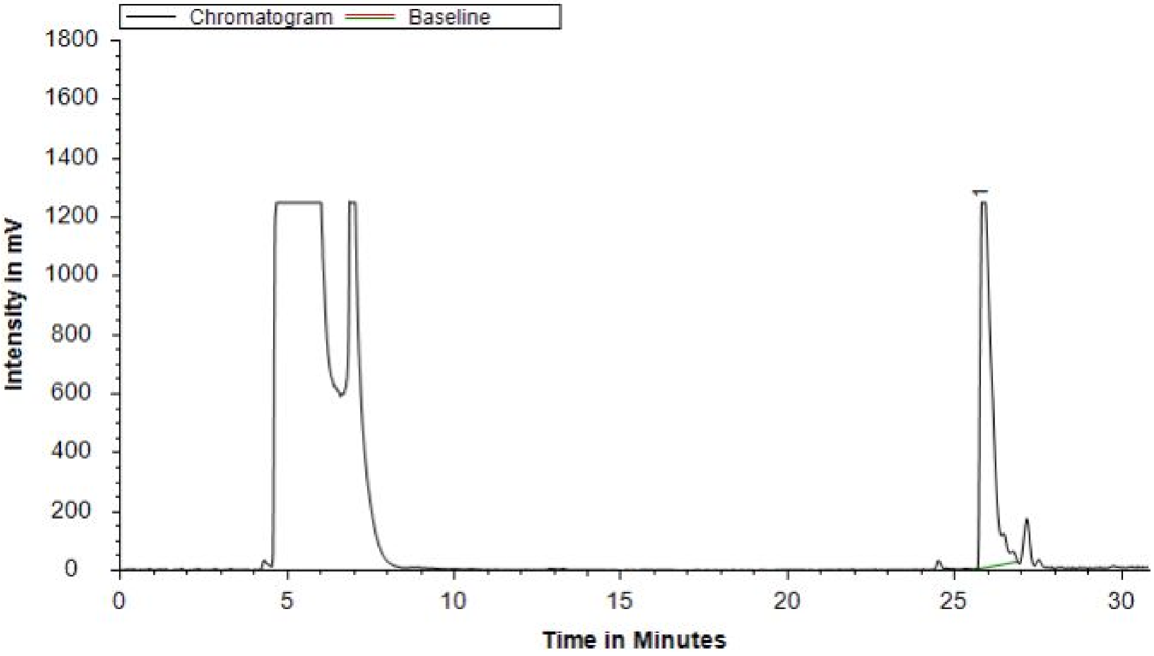

Result Table

No.	Ret. Time	Height	Area	Percent	Name
1	25.81667	1241.566	2.981809E+07	100	

ESI-MS:

Target mass: 1633.8 g/mol

Detected mass: 1634.5 $[M]^{+}$, 817.6 $[M/2]^{+}$

Acquisition Parameter

Ion Source Type	ESI	Ion Polarity	Positive	Alternating Ion Polarity	off
Mass Range Mode	Std/Normal	Scan Begin	400 m/z	Scan End	2000 m/z
Capillary Exit	213.5 Volt	Skimmer	40.0 Volt	Trap Drive	130.2
Accumulation Time	87 µs	Averages	20 Spectra	Auto MS/MS	off

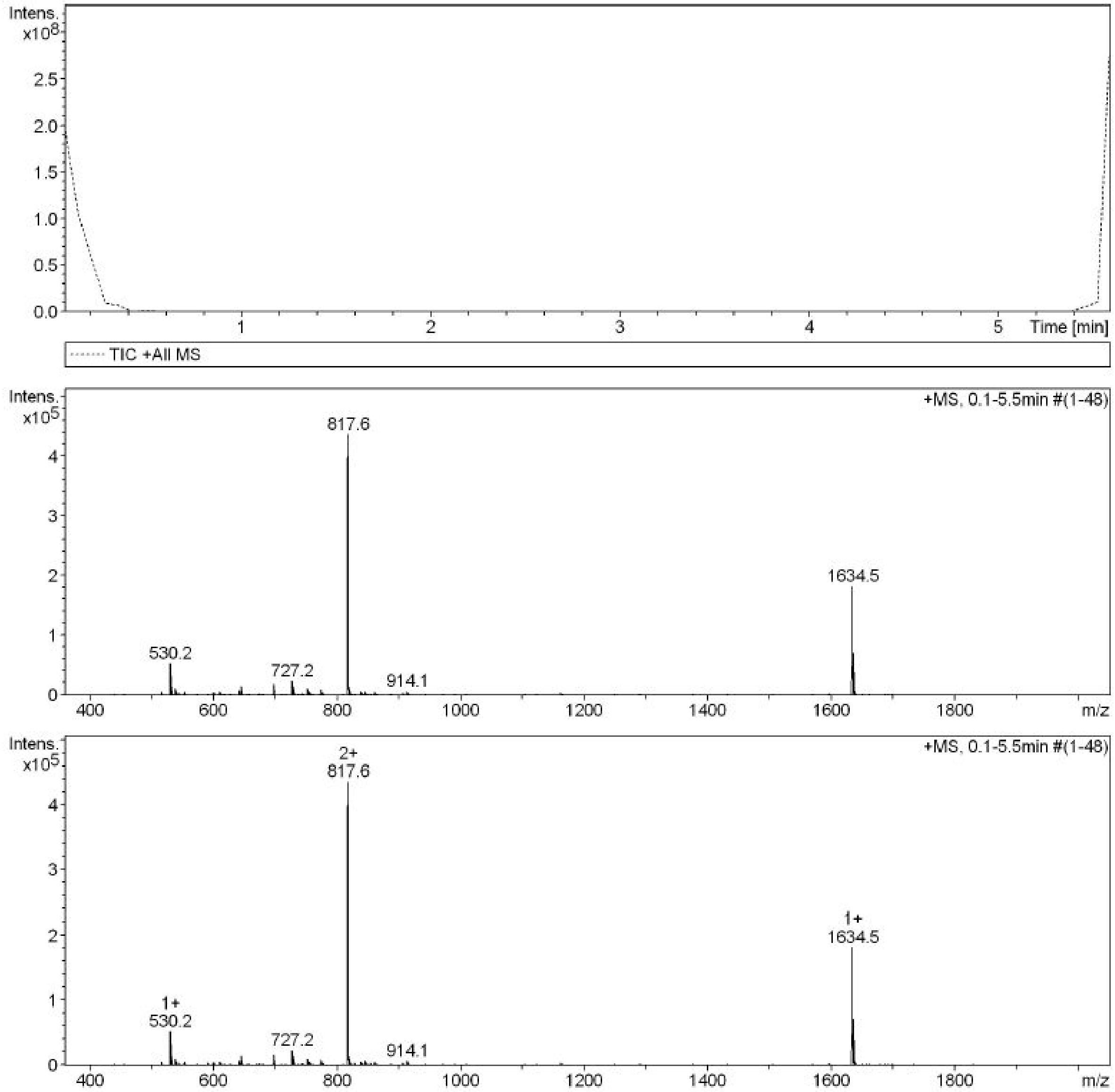

Analytical HPLC:

214 nm

Chromatogram and Results

Injection Details

Injection Name:	**DOTA-KP34-EEE 214nm**	*Run Time (min):*	**24,00**
Vial Number:	**RB2**	*Injection Volume:*	**10,00**
Injection Type:	**Unknown**	*Channel:*	**UV_VIS_1**
Calibration Level:		*Wavelength:*	**214**
Instrument Method:	**5_95 in 17min 0,8ml 214nm Auto**	*Bandwidth:*	**n.a.**
Processing Method:	**Basic Quantitative**	*Dilution Factor:*	**1,0000**
Injection Date/Time:	**25.Jul.25 13:28**	*Sample Weight:*	**1,0000**

Chromatogram

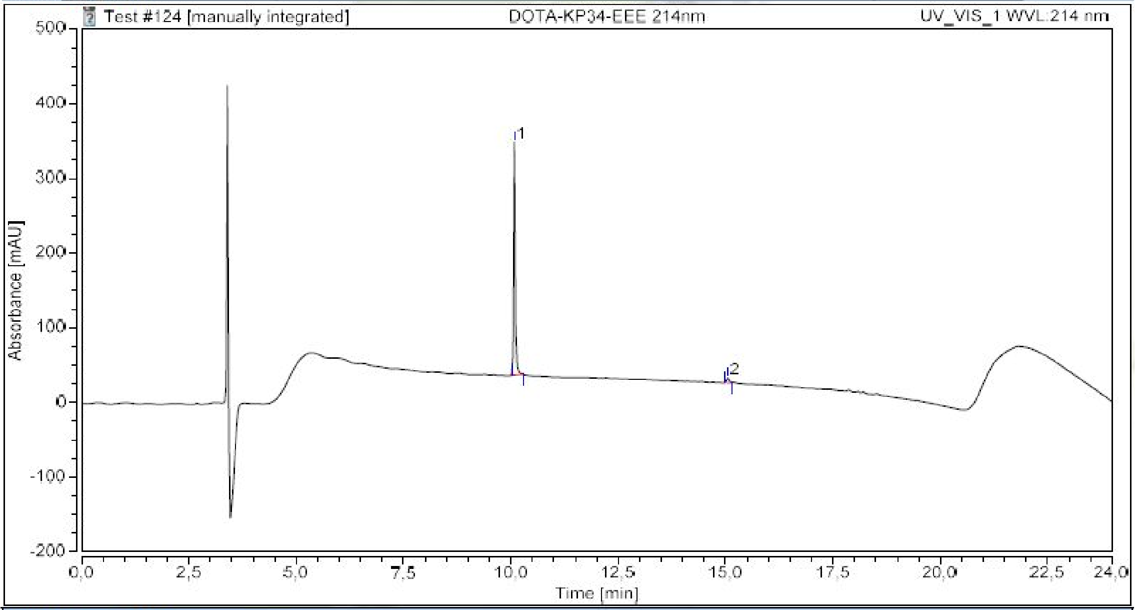

Integration Results

No.	Peak Name	Retention Time min	Area mAU*min	Height mAU	Relative Area %	Relative Height %	Amount n.a.
1	n.a.	10,077	14,348	311,496	97,05	98,01	n.a.
2	n.a.	15,052	0,436	6,324	2,95	1,99	n.a.
**Total:**			**14,785**	**317,819**	**100,00**	**100,00**	

254 nm

Chromatogram and Results

Injection Details

Injection Name:	**DOTA-K34-EEE 254nm**	*Run Time (min):*	**20,00**
Vial Number:	**RC1**	*Injection Volume:*	**10,00**
Injection Type:	**Unknown**	*Channel:*	**UV_VIS_1**
Calibration Level:		*Wavelength:*	**254**
Instrument Method:	**5_95 in 17min 0,8ml 254nm Auto**	*Bandwidth:*	**n.a.**
Processing Method:	**Basic Quantitative**	*Dilution Factor:*	**1,0000**
Injection Date/Time:	**07.Jul.25 14:17**	*Sample Weight:*	**1,0000**

Chromatogram

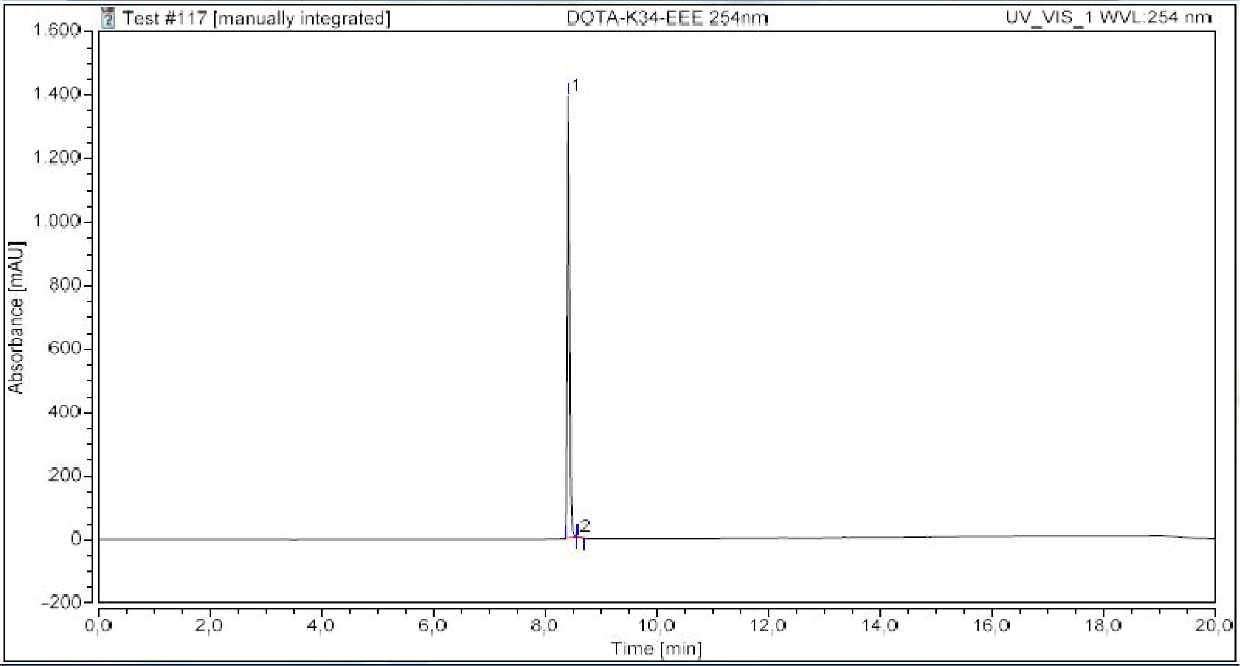

Integration Results

No.	Peak Name	Retention Time min	Area mAU*min	Height mAU	Relative Area %	Relative Height %	Amount n.a.
1	n.a.	8,417	60,272	1392,136	99,86	99,87	n.a.
2	n.a.	8,593	0,086	1,798	0,14	0,13	n.a.
**Total:**			**60,358**	**1393,934**	**100,00**	**100,00**	

1.3 AF-488-KPs

Table S3. List of synthesized AF-488-KPs.

Substance name	Structure	Mol. Weight [g/mol]	Yield [%]
AF-488-KP-54	AF-488-Gly-Thr-Ser-Leu-Ser-Pro-Pro-Pro-Glu-Ser-Ser-Gly-Ser-Arg-Gln-Gln-Pro-Gly-Leu-Ser-Ala-Pro-His-Ser-Arg-Gln-Ile-Pro-Ala-Pro-Gln-Gly-Ala-Val-Leu-Val-Gln-Arg-Glu-Lys-Asp-Leu-Pro-Asn-Tyr-Asn-Trp-Asn-Ser-Phe-Gly-Leu-Arg-Phe-NH_2	6375.0	--*
AF-488-KP-10	AF-488-Tyr-Asn-Trp-Asn-Ser-Phe-Gly-Leu-Arg-Phe-NH_2	1819.9	6
AF-488-KP-10-E3	AF-488-Glu-Glu-Glu-Tyr-Asn-Trp-Asn-Ser-Phe-Gly-Leu-Arg-Phe-NH_2	2207.3	2
AF-488-KiSS-34	AF-488-AMBA-2-Nal-Gly-Leu-Arg-Trp-NH_2	1376.5	7

*commercially purchased

AF-488-KP-10

HPLC chromatogram (HILIC):

(A) Water + 0.2% Formic acid (FA), (B) ACN + 0.2% FA; Gradient: 0–40 min 97–50% B; Flow: 15 mL/min; Wavelength: 214 nm; Temperature: RT.

Retention time: 31.03 min

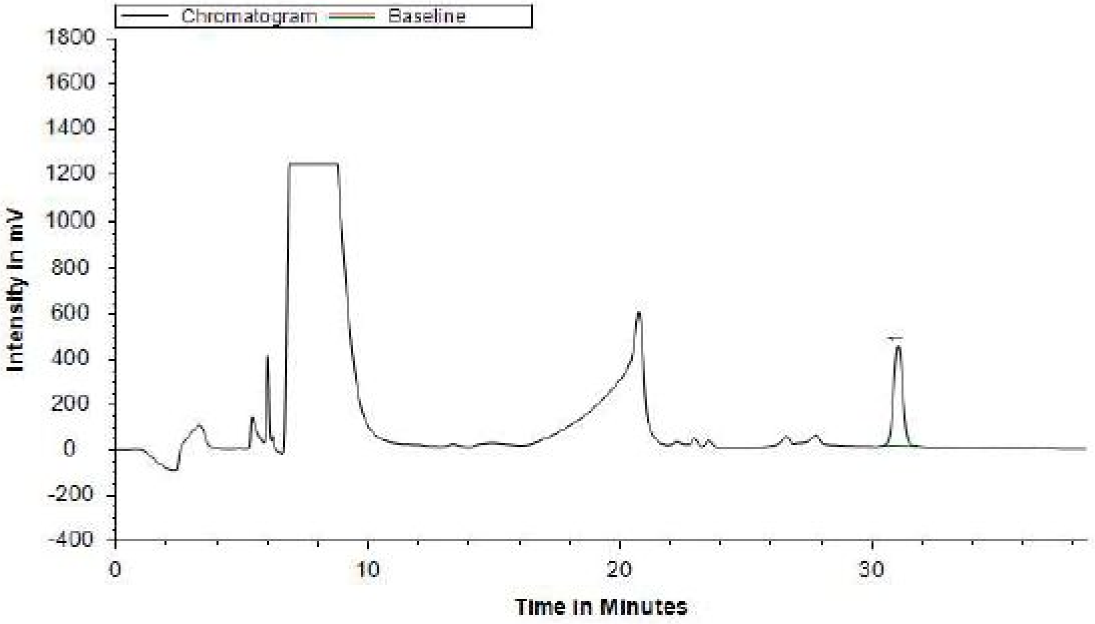

Result Table

No.	Ret. Time	Height	Area	Percent	Name
1	31,03333	438,3823	1,064133E+07	100	

ESI-MS:

Target mass: 1819.9 g/mol

Detected mass: 1819.4 $[M]^+$, 909.5 $[M/2]^+$

Acquisition Parameter

Ion Source Type	ESI	Ion Polarity	Positive	Alternating Ion Polarity	off		
Mass Range Mode	Std/Normal	Scan Begin	500 m/z	Scan End	2500 m/z		
Capillary Exit	227.4 Volt	Skimmer	40.0 Volt	Trap Drive	142.8		
Accumulation Time	190413 µs	Averages	10 Spectra	Auto MS/MS	off		

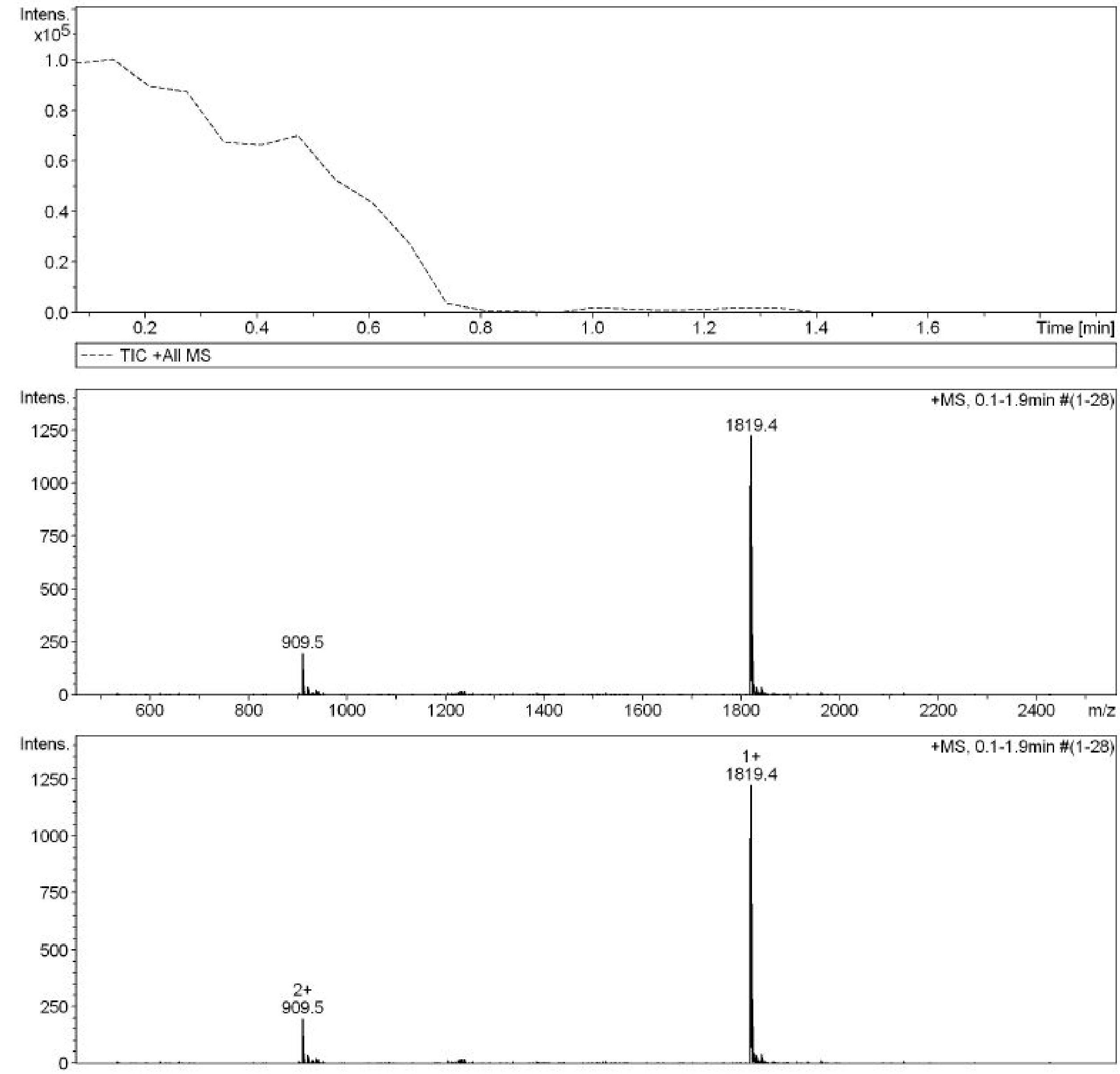

Analytical HPLC:

214 nm

Chromatogram and Results

Injection Details

Injection Name:	**AF488-KP10 214nm**	*Run Time (min):*	**24,00**
Vial Number:	**RC1**	*Injection Volume:*	**10,00**
Injection Type:	**Unknown**	*Channel:*	**UV_VIS_1**
Calibration Level:		*Wavelength:*	**214**
Instrument Method:	**5_95 in 17min 0,8ml 214nm Auto**	*Bandwidth:*	**n.a.**
Processing Method:	**Basic Quantitative**	*Dilution Factor:*	**1,0000**
Injection Date/Time:	**25.Jul.25 18:32**	*Sample Weight:*	**1,0000**

Chromatogram

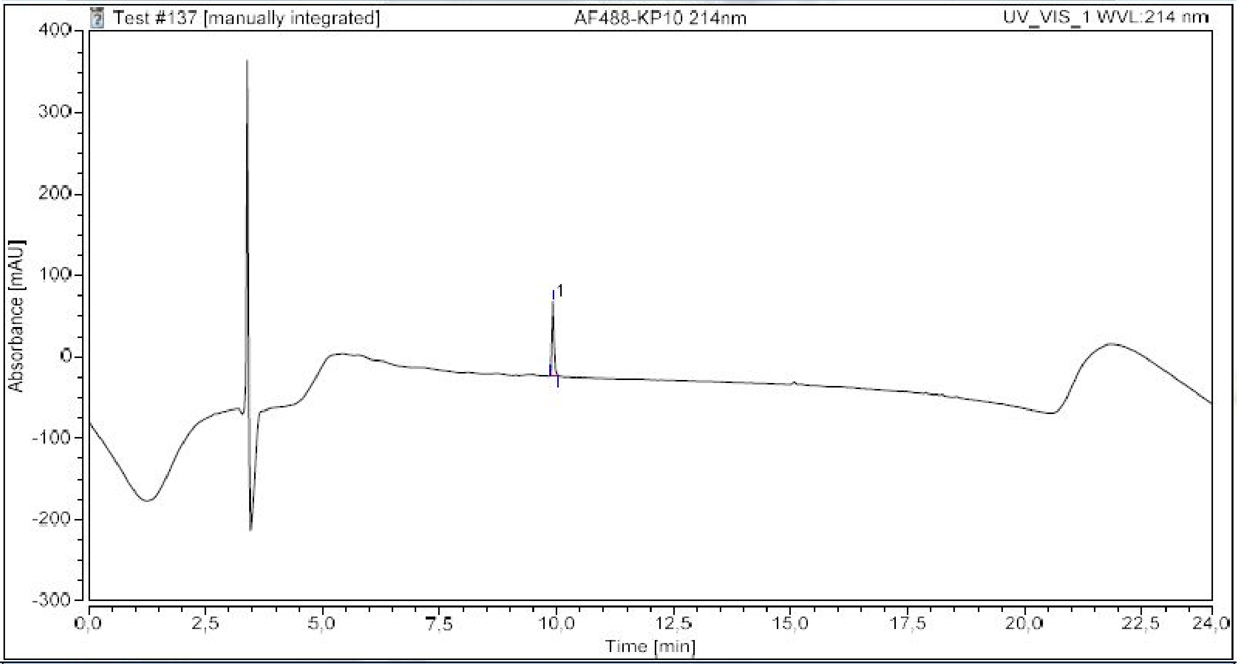

Integration Results

No.	Peak Name	Retention Time min	Area mAU*min	Height mAU	Relative Area %	Relative Height %	Amount n.a.
1	n.a.	9,917	4,491	91,846	100,00	100,00	n.a.
Total:			**4,491**	**91,846**	**100,00**	**100,00**	

254 nm

Chromatogram and Results

Injection Details

Injection Name:	**AF488-KP10 254nm**	*Run Time (min):*	**20,00**
Vial Number:	**RA2**	*Injection Volume:*	**10,00**
Injection Type:	**Unknown**	*Channel:*	**UV_VIS_1**
Calibration Level:		*Wavelength:*	**254**
Instrument Method:	**5_95 in 17min 0,8ml 254nm Auto**	*Bandwidth:*	**n.a.**
Processing Method:	**Basic Quantitative**	*Dilution Factor:*	**1,0000**
Injection Date/Time:	**25.Jul.25 18:57**	*Sample Weight:*	**1,0000**

Chromatogram

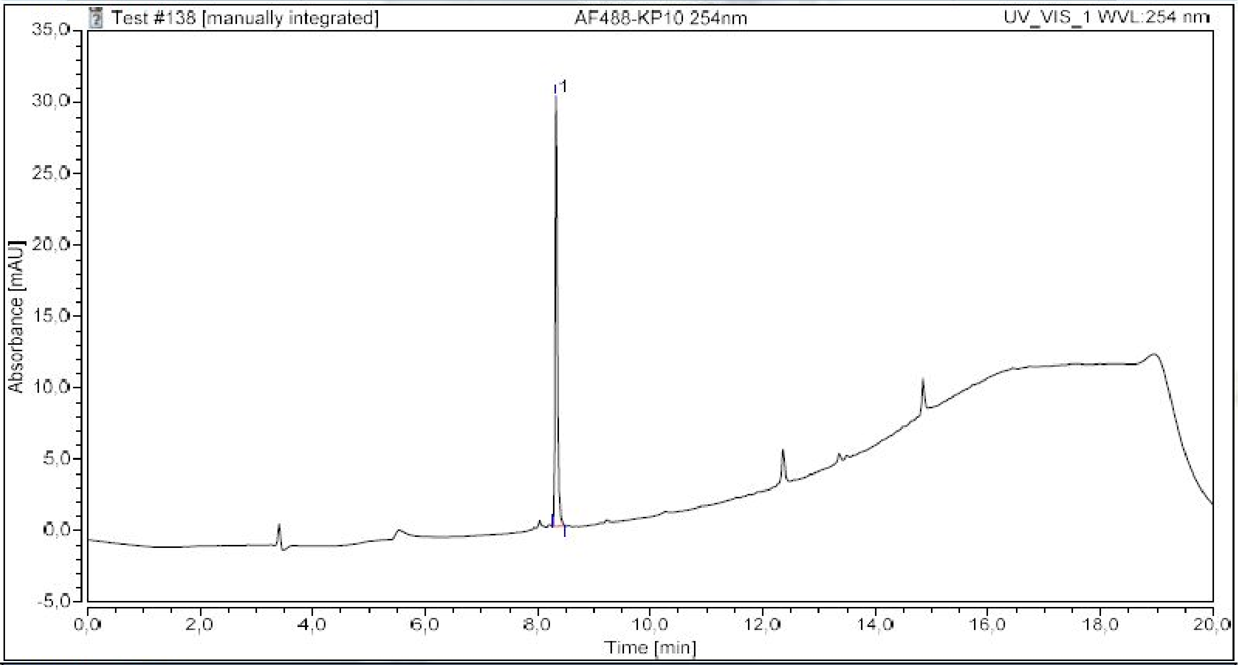

Integration Results

No.	Peak Name	Retention Time min	Area mAU*min	Height mAU	Relative Area %	Relative Height %	Amount n.a.
1	n.a.	8,327	1,405	30,123	100,00	100,00	n.a.
**Total:**			**1,405**	**30,123**	**100,00**	**100,00**	

AF-488-KP-10-EEE

HPLC chromatogram (HILIC):

(A) Water + 0.2% Formic acid (FA), (B) ACN + 0.2% FA; Gradient: 0–40 min 97–60% B; Flow: 20 mL/min; Wavelength: 214 nm; Temperature: RT.

Retention time: 27.23 min

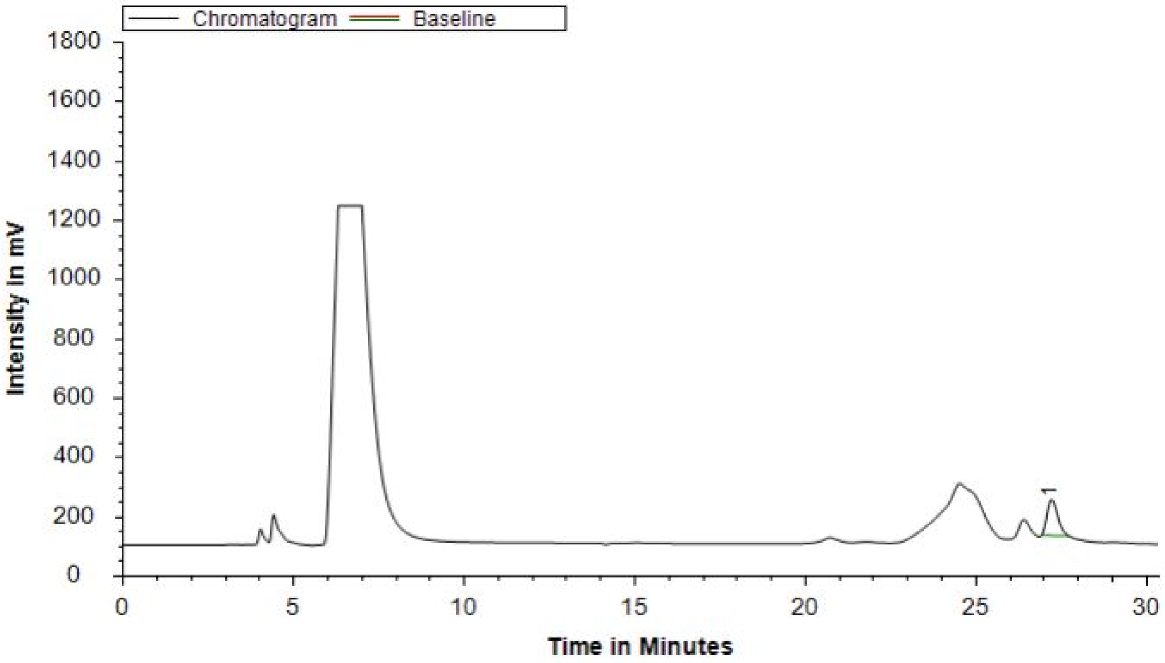

Result Table

No.	Ret. Time	Height	Area	Percent	Name
1	27.225	120.3485	2597545	100	

ESI-MS:

Target mass: 2207.3 g/mol

Detected mass: 2206.5 $[M]^+$, 1103.7 $[M/2]^+$

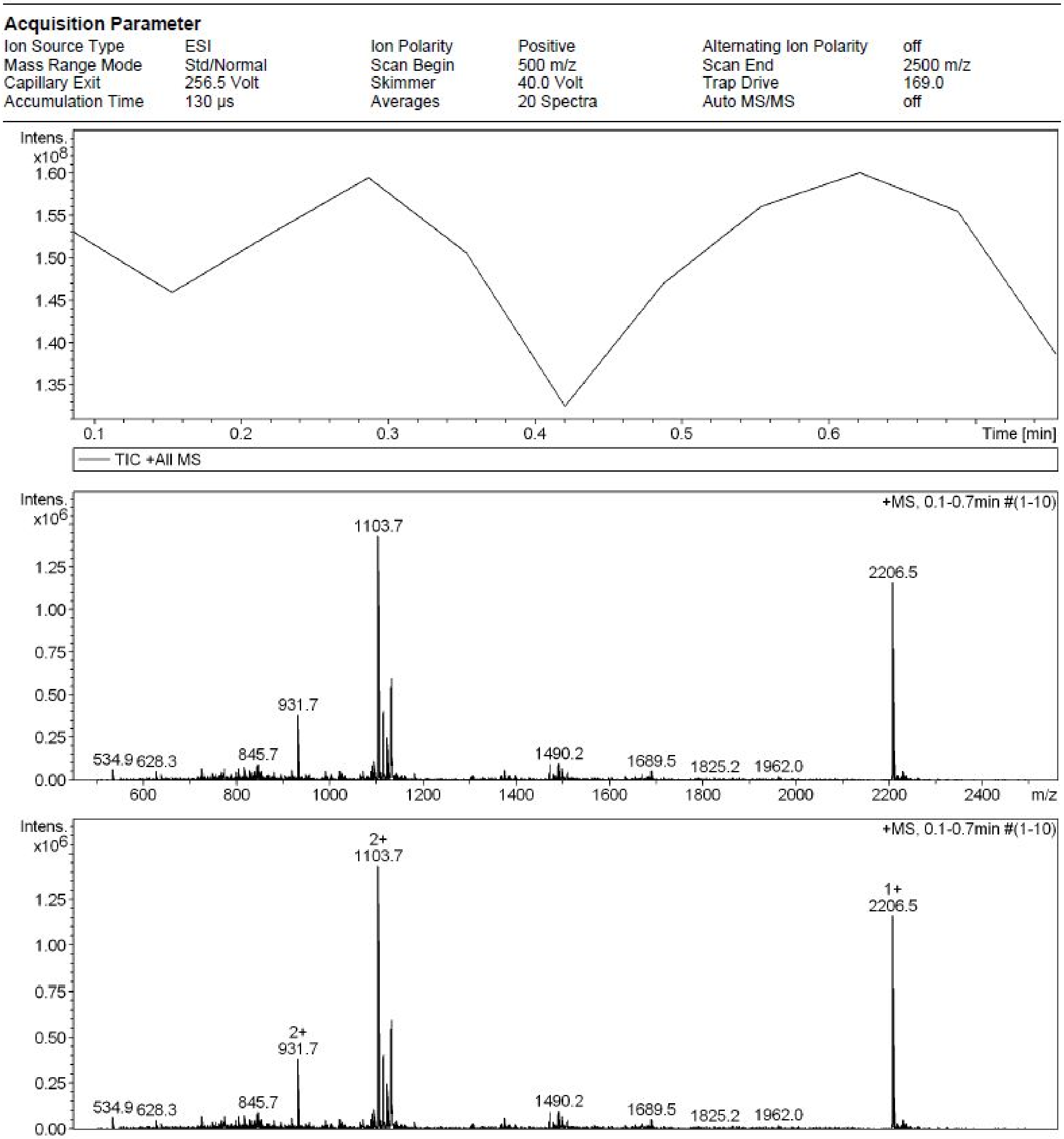

Analytical HPLC:

214 nm

Chromatogram and Results

Injection Details

Injection Name:	**AF488-KP10-EEE 214nm**	*Run Time (min):*	**24,00**
Vial Number:	**RC3**	*Injection Volume:*	**10,00**
Injection Type:	**Unknown**	*Channel:*	**UV_VIS_1**
Calibration Level:		*Wavelength:*	**214**
Instrument Method:	**5_95 in 17min 0,8ml 214nm Auto**	*Bandwidth:*	**n.a.**
Processing Method:	**Basic Quantitative**	*Dilution Factor:*	**1,0000**
Injection Date/Time:	**16.Jul.25 15:41**	*Sample Weight:*	**1,0000**

Chromatogram

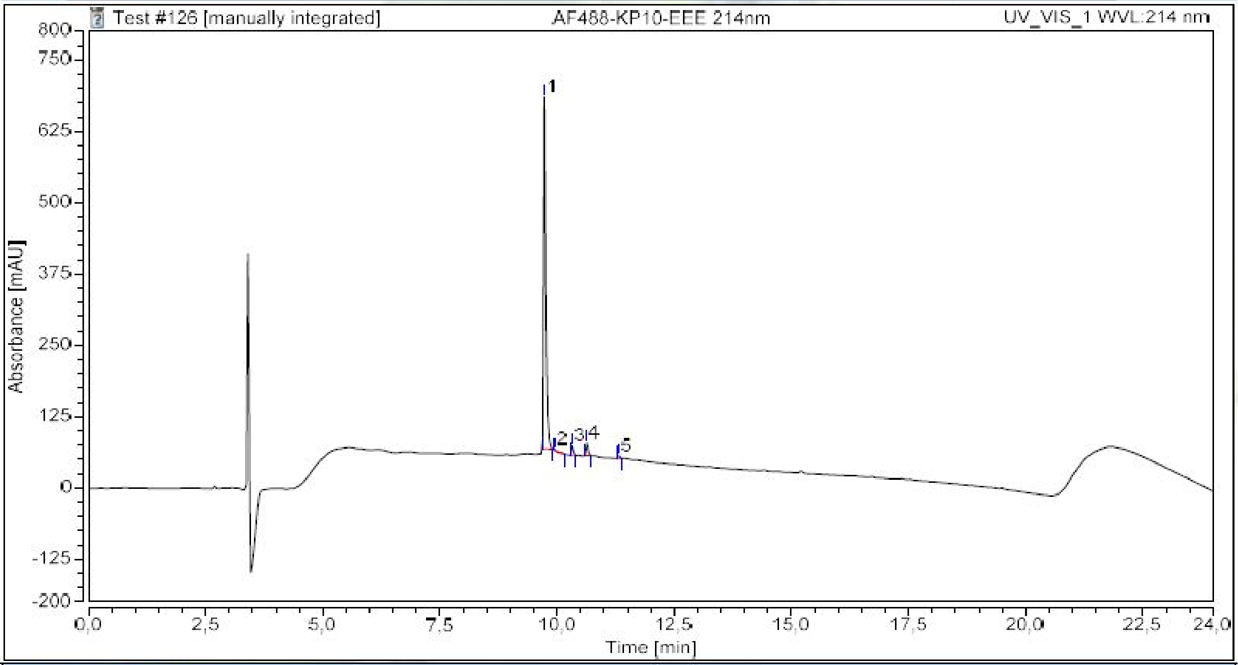

Integration Results

No.	Peak Name	Retention Time min	Area mAU*min	Height mAU	Relative Area %	Relative Height %	Amount n.a.
1	n.a.	9,732	31,807	616,499	94,15	93,17	n.a.
2	n.a.	9,948	0,028	1,868	0,08	0,28	n.a.
3	n.a.	10,330	0,689	16,662	2,04	2,52	n.a.
4	n.a.	10,638	1,042	21,802	3,09	3,29	n.a.
5	n.a.	11,318	0,217	4,827	0,64	0,73	n.a.
Total:			**33,783**	**661,658**	**100,00**	**100,00**	

254 nm

Chromatogram and Results

Injection Details

Injection Name:	AF488-KP10-EEE 254nm	*Run Time (min):*	20,00
Vial Number:	RC4	*Injection Volume:*	10,00
Injection Type:	Unknown	*Channel:*	UV_VIS_1
Calibration Level:		*Wavelength:*	254
Instrument Method:	5_95 in 17min 0,8ml 254nm Auto	*Bandwidth:*	n.a.
Processing Method:	Basic Quantitative	*Dilution Factor:*	1,0000
Injection Date/Time:	16.Jul.25 16:06	*Sample Weight:*	1,0000

Chromatogram

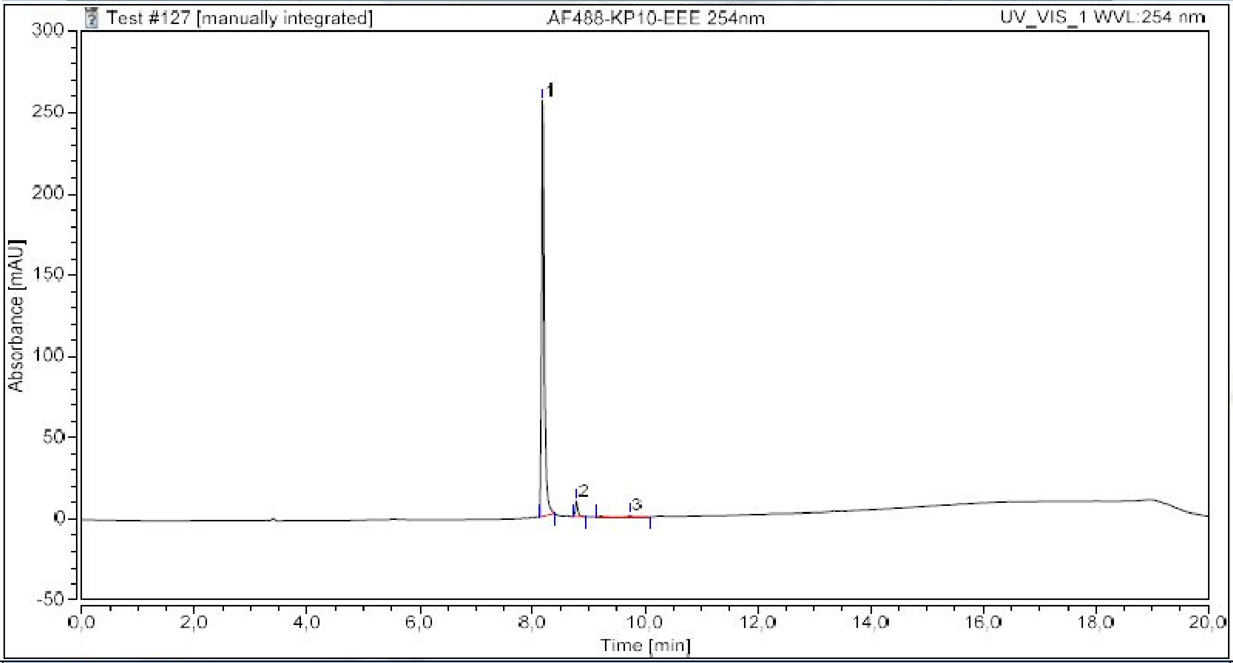

Integration Results

No.	Peak Name	Retention Time min	Area mAU*min	Height mAU	Relative Area %	Relative Height %	Amount n.a.
1	n.a.	8,182	12,746	255,884	96,29	96,00	n.a.
2	n.a.	8,782	0,468	9,473	3,54	3,55	n.a.
3	n.a.	9,732	0,023	1,182	0,17	0,44	n.a.
Total:			13,237	266,540	100,00	100,00	

AF-488-KiSS-34

HPLC chromatogram (HILIC):

(A) Water + 0.2% Formic acid (FA), (B) ACN + 0.2% FA; Gradient: 0–40 min 97–50% B; Flow: 15 mL/min; Wavelength: 214 nm; Temperature: RT.

Retention time: 29.33 min

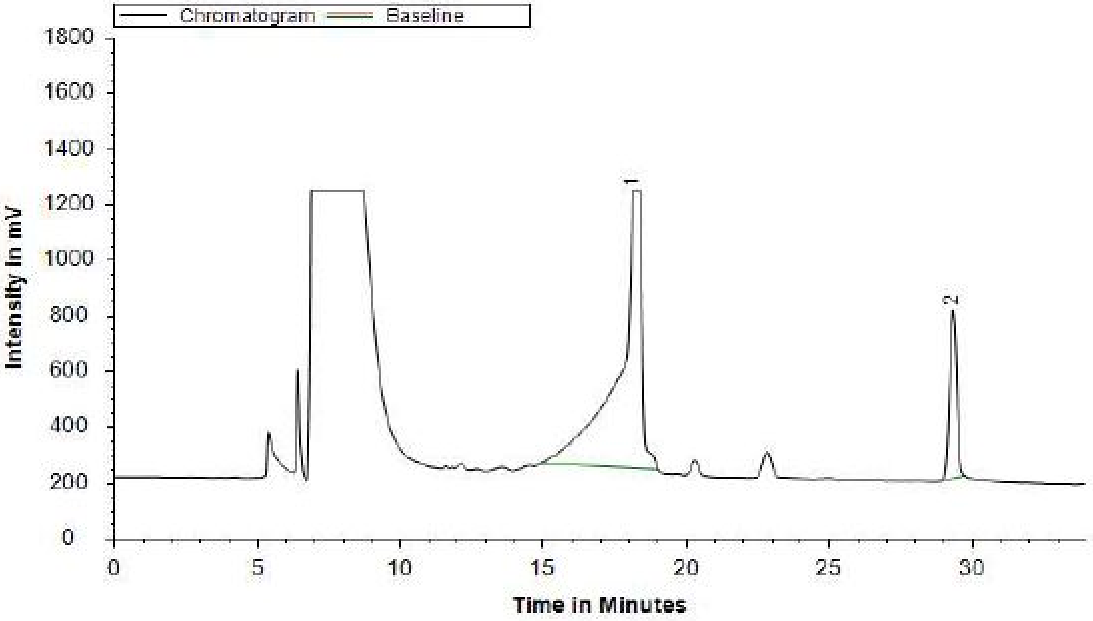

Result Table

No.	Ret. Time	Height	Area	Percent	Name
1	18,1	992,0529	5,603489E+07	84,1566	
2	29,325	599,897	1,054918E+07	15,8434	

ESI-MS:

Target mass: 1376.5 g/mol

Detected mass: 1376.2 $[M]^+$

Acquisition Parameter

Ion Source Type	ESI	Ion Polarity	Positive	Alternating Ion Polarity	off
Mass Range Mode	Std/Normal	Scan Begin	100 m/z	Scan End	1500 m/z
Capillary Exit	194.2 Volt	Skimmer	40.0 Volt	Trap Drive	112.8
Accumulation Time	10542 µs	Averages	20 Spectra	Auto MS/MS	off

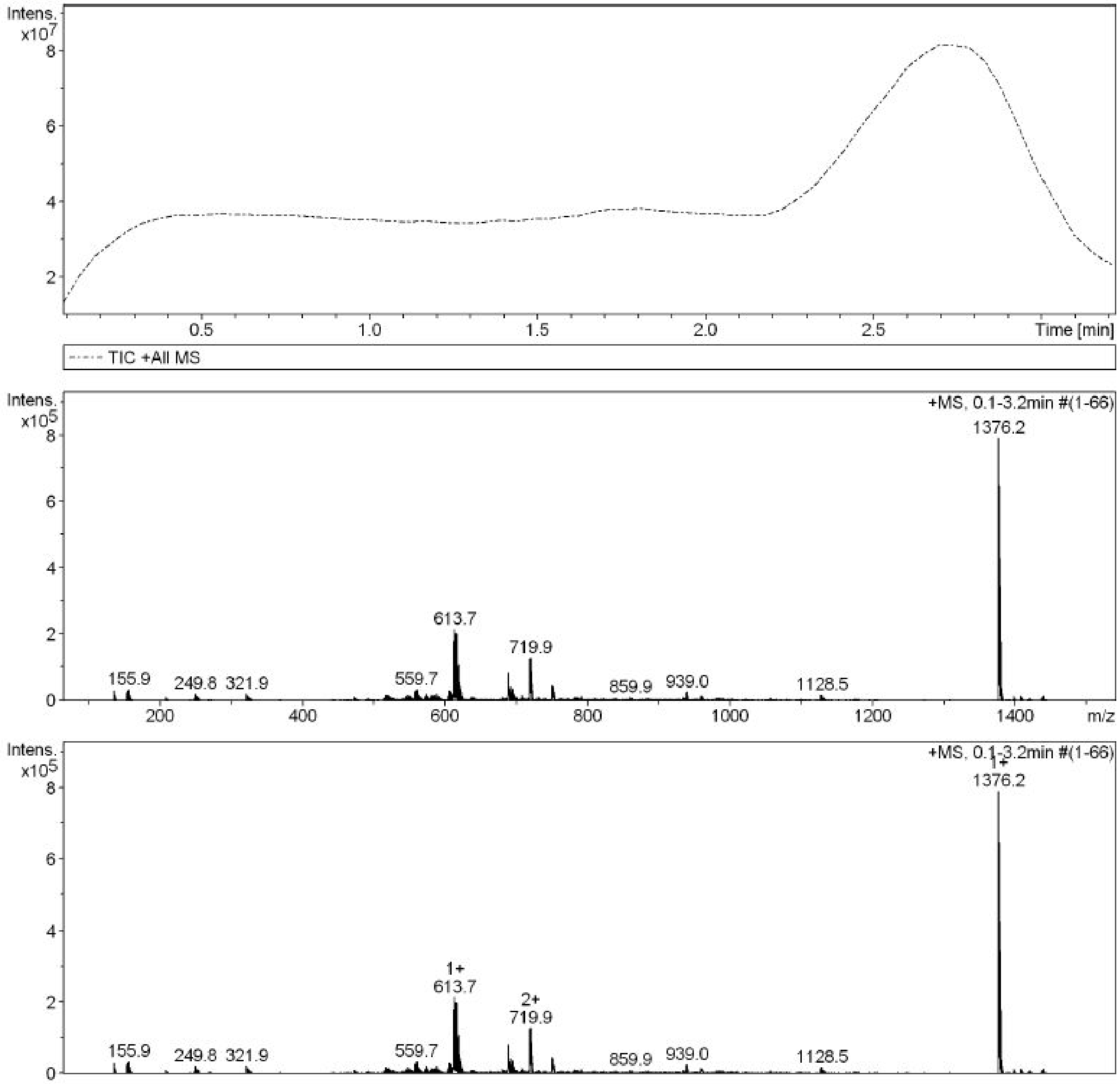

Analytical HPLC:

214, 254, 220 nm

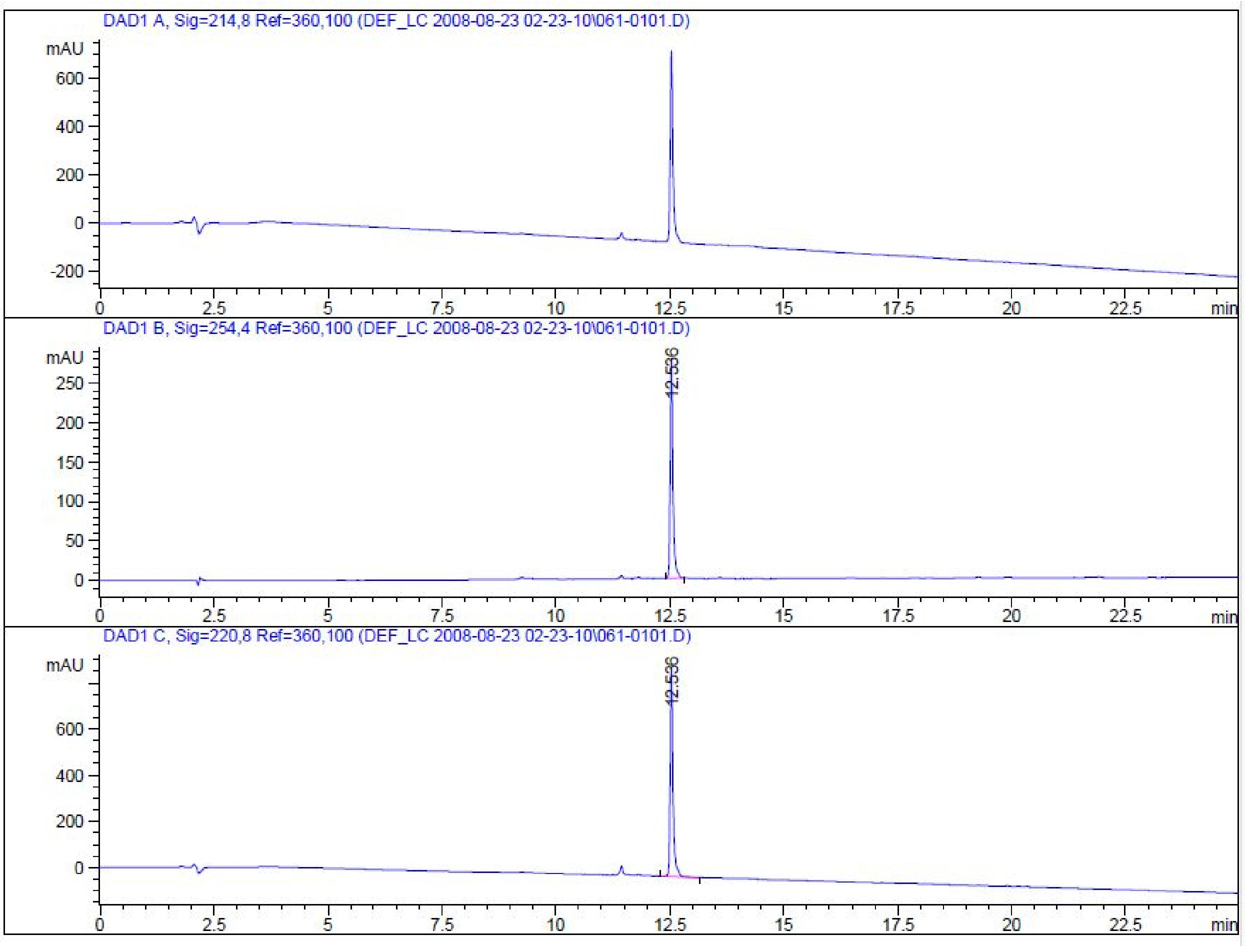

1.4 Scrambled KPs and DOTA and AF-488 conjugates

Table S4. List of synthesized scrambled KPs and corresponding DOTA and AF-488 conjugates.

Substance Name	Structure	Mol. Weight [g/mol]	Yield [%]
**KP-10s**	Ser-Tyr-Phe-Asn-Trp-Asn-Phe-Arg-Leu-Gly-NH_2	1302.4	34
**AF-488-KP-10s**	AF-488-Ser-Tyr-Phe-Asn-Trp-Asn-Phe-Arg-Leu-Gly-NH_2	1819.9	7
**DOTA-KP-10s**	DOTA-Ser-Tyr-Phe-Asn-Trp-Asn-Phe-Arg-Leu-Gly-NH_2	1688.8	14
**KiSS-34s**	Leu-Trp-2-Nal-Arg-AMBA-Gly-NH_2	860.0	36
**AF-488-KiSS-34s**	AF-488-Leu-Trp-2-Nal-Arg-AMBA-Gly-NH_2	1376.5	11
**DOTA-KiSS-34s**	DOTA-Leu-Trp-2-Nal-Arg-AMBA-Gly-NH_2	1246.4	14

KP-10s

HPLC chromatogram:

(A) Water + 0.1% Trifluoroacetic acid (TFA), (B) ACN + 0.1% TFA; Gradient: 0–40 min 5–95% B; Flow: 30 mL/min; Wavelength: 214 nm; Temperature: RT.

Retention time: 17.7 min

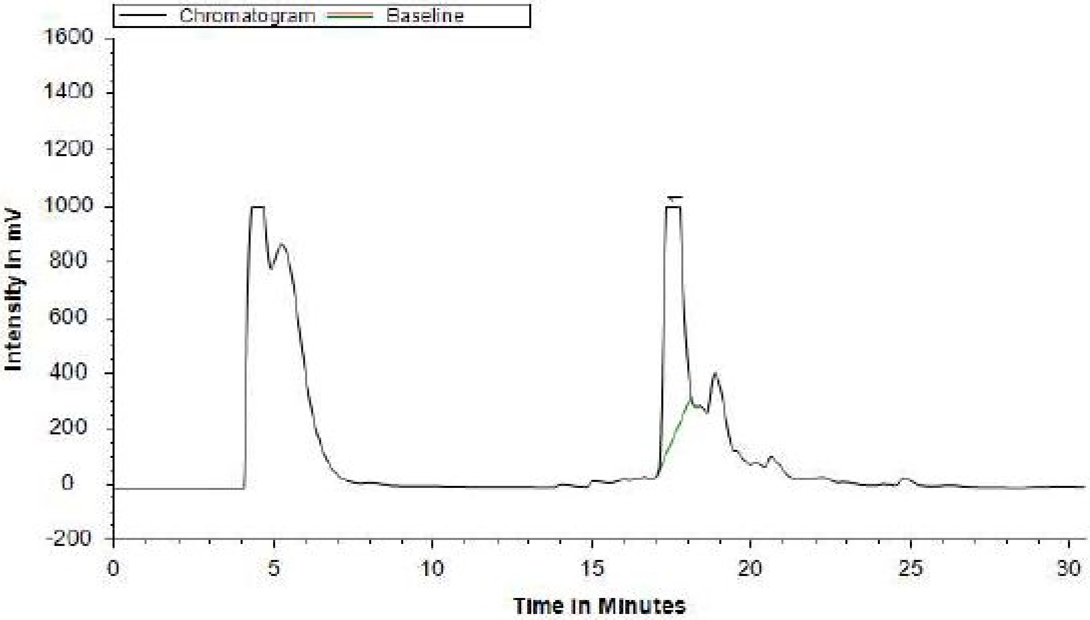

Result Table

No.	Ret. Time	Height	Area	Percent	Name
1	17,7	789,7073	3,308386E+07	100	

ESI-MS:

Target mass: 1302.4 g/mol

Detected mass: 1302.4 $[M]^{+}$, 651.6 $[M/2]^{+}$

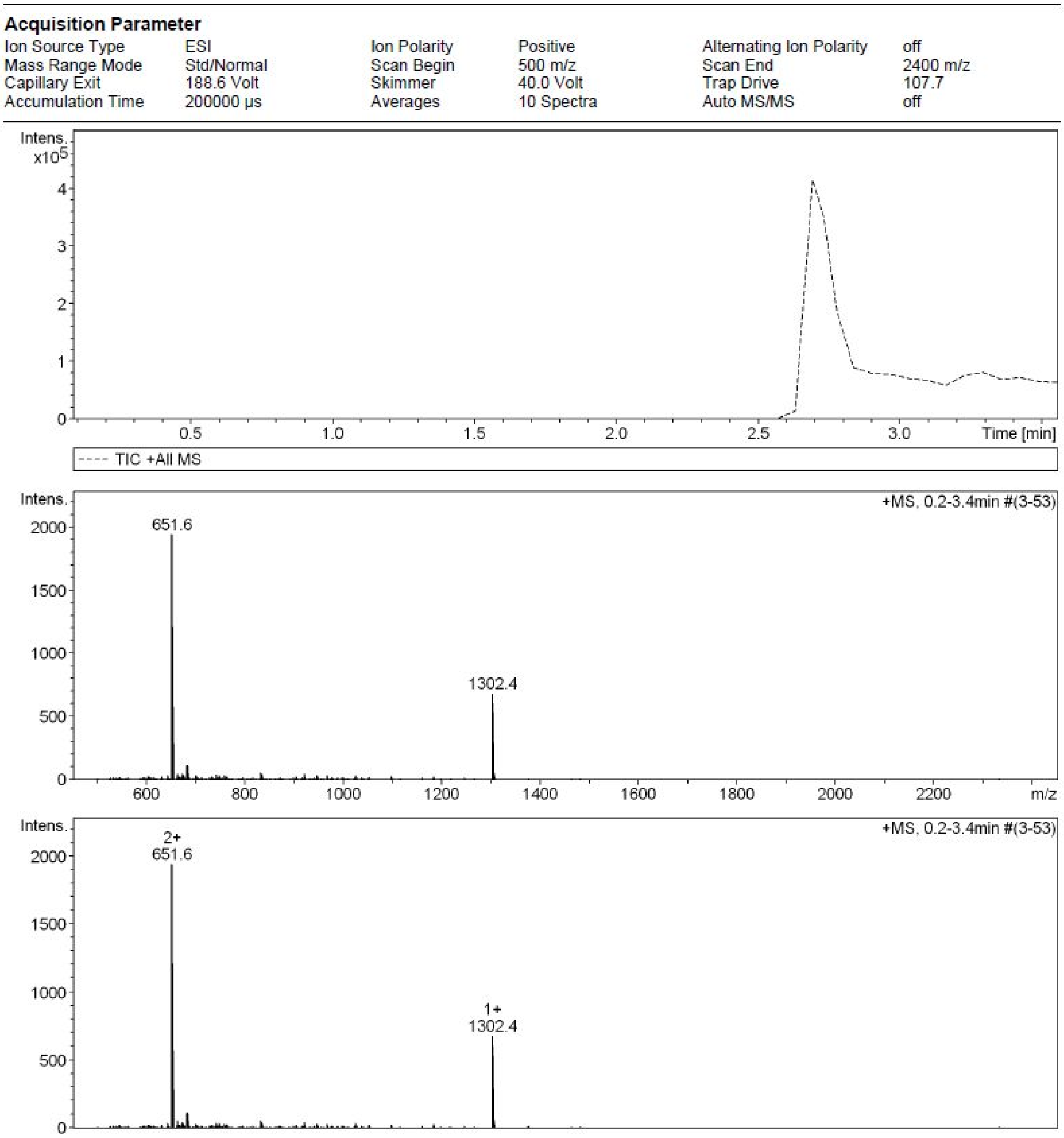

Analytical HPLC:

214 nm

Chromatogram and Results

Injection Details

Injection Name:	**KP10s 214nm**	*Run Time (min):*	**24,00**
Vial Number:	**RE1**	*Injection Volume:*	**10,00**
Injection Type:	**Unknown**	*Channel:*	**UV_VIS_1**
Calibration Level:		*Wavelength:*	**214**
Instrument Method:	**5_95 in 17min 0,8ml 214nm Auto**	*Bandwidth:*	**n.a.**
Processing Method:	**Basic Quantitative**	*Dilution Factor:*	**1,0000**
Injection Date/Time:	**28.Jul.25 12:40**	*Sample Weight:*	**1,0000**

Chromatogram

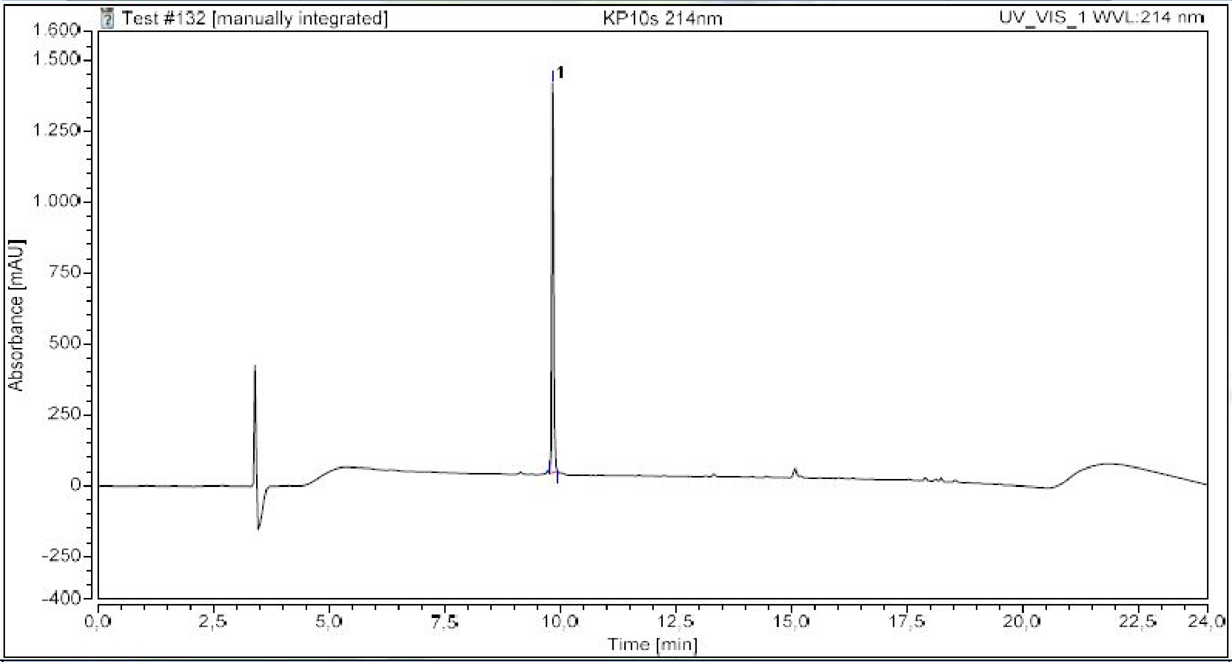

Integration Results

No.	Peak Name	Retention Time min	Area mAU*min	Height mAU	Relative Area %	Relative Height %	Amount n.a.
1	n.a.	9,833	56,607	1373,104	100,00	100,00	n.a.
**Total:**			**56,607**	**1373,104**	**100,00**	**100,00**	

254 nm

Chromatogram and Results

Injection Details

Injection Name:	**KP-10s 254nm**	*Run Time (min):*	**20,00**
Vial Number:	**RB2**	*Injection Volume:*	**10,00**
Injection Type:	**Unknown**	*Channel:*	**UV_VIS_1**
Calibration Level:		*Wavelength:*	**254**
Instrument Method:	**5_95 in 17min 0,8ml 254nm Auto**	*Bandwidth:*	**n.a.**
Processing Method:	**Basic Quantitative**	*Dilution Factor:*	**1,0000**
Injection Date/Time:	**02.Mai.25 11:40**	*Sample Weight:*	**1,0000**

Chromatogram

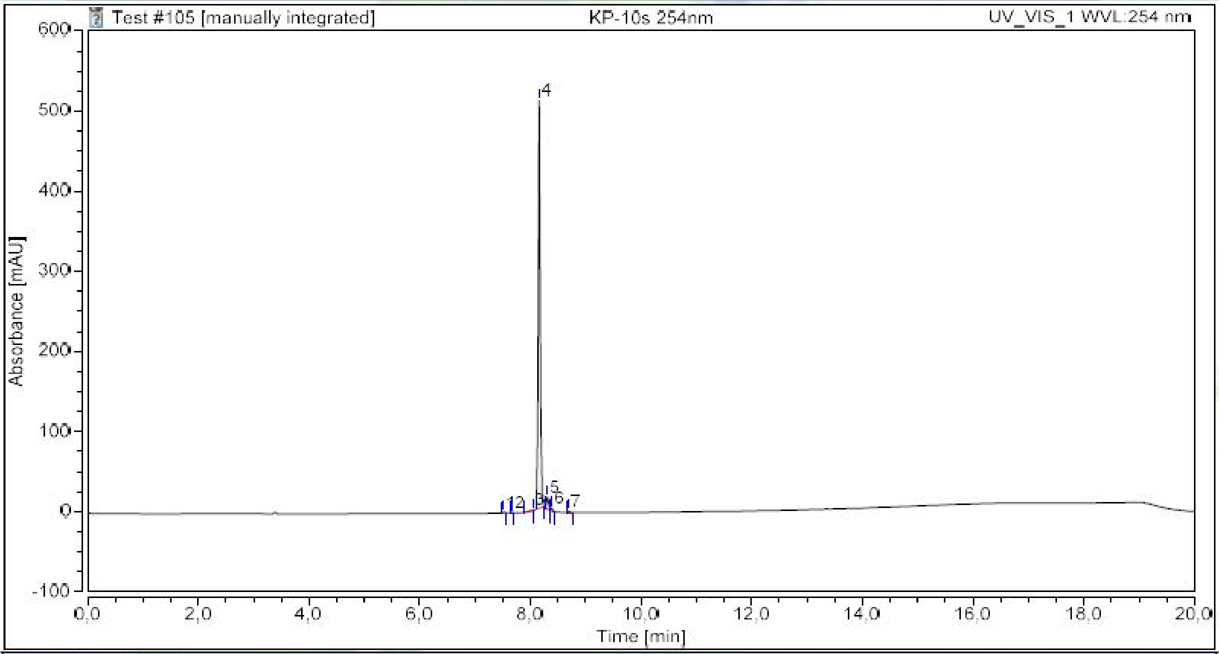

Integration Results

No.	Peak Name	Retention Time min	Area mAU*min	Height mAU	Relative Area %	Relative Height %	Amount n.a.
1	n.a.	7,517	0,052	1,391	0,23	0,26	n.a.
2	n.a.	7,677	0,021	0,554	0,10	0,10	n.a.
3	n.a.	8,042	0,102	1,121	0,46	0,21	n.a.
4	n.a.	8,157	21,031	508,756	95,46	95,27	n.a.
5	n.a.	8,312	0,598	15,562	2,71	2,91	n.a.
6	n.a.	8,393	0,137	4,215	0,62	0,79	n.a.
7	n.a.	8,693	0,091	2,428	0,41	0,45	n.a.
**Total:**			**22,031**	**534,027**	**100,00**	**100,00**	

AF-488-KP-10s

HPLC chromatogram (HILIC):

(A) Water + 0.2% Formic acid (FA), (B) ACN + 0.2% FA; Gradient: 0–40 min 97–50% B; Flow: 15 mL/min; Wavelength: 214 nm; Temperature: RT.

Retention time: 31.79 min

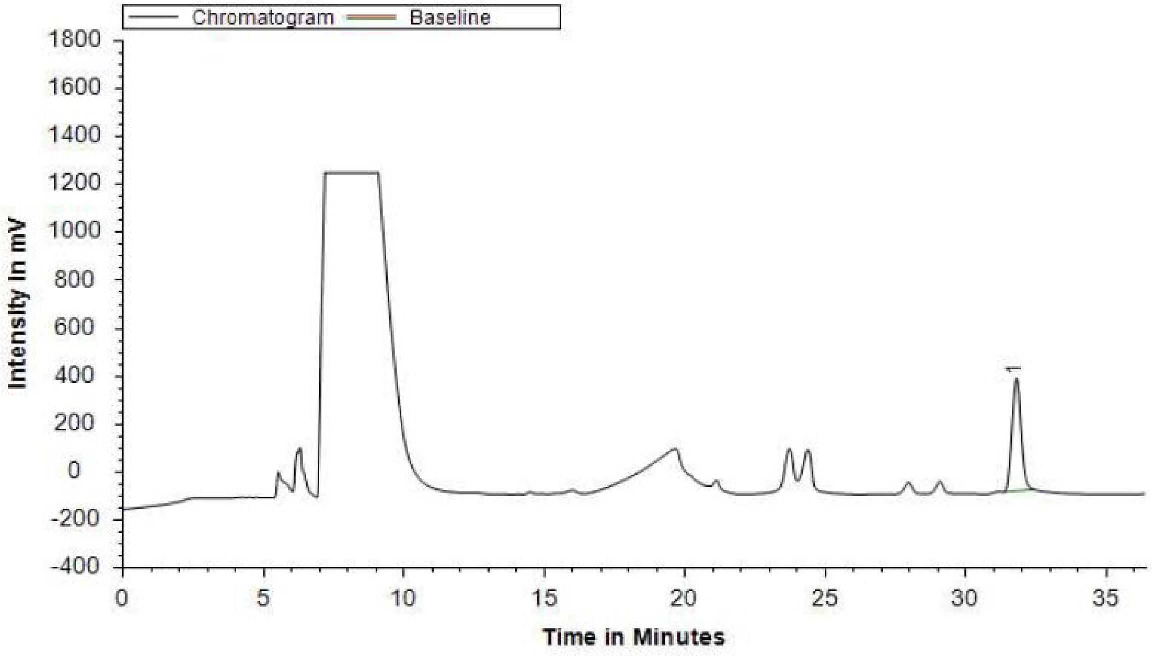

Result Table

No.	Ret. Time	Height	Area	Percent	Name
1	31.79167	469.0567	1.041988E+07	100	

ESI-MS:

Target mass: 1819.9 g/mol

Detected mass: 1818.4 $[M]^{+}$, 909.5 $[M/2]^{+}$

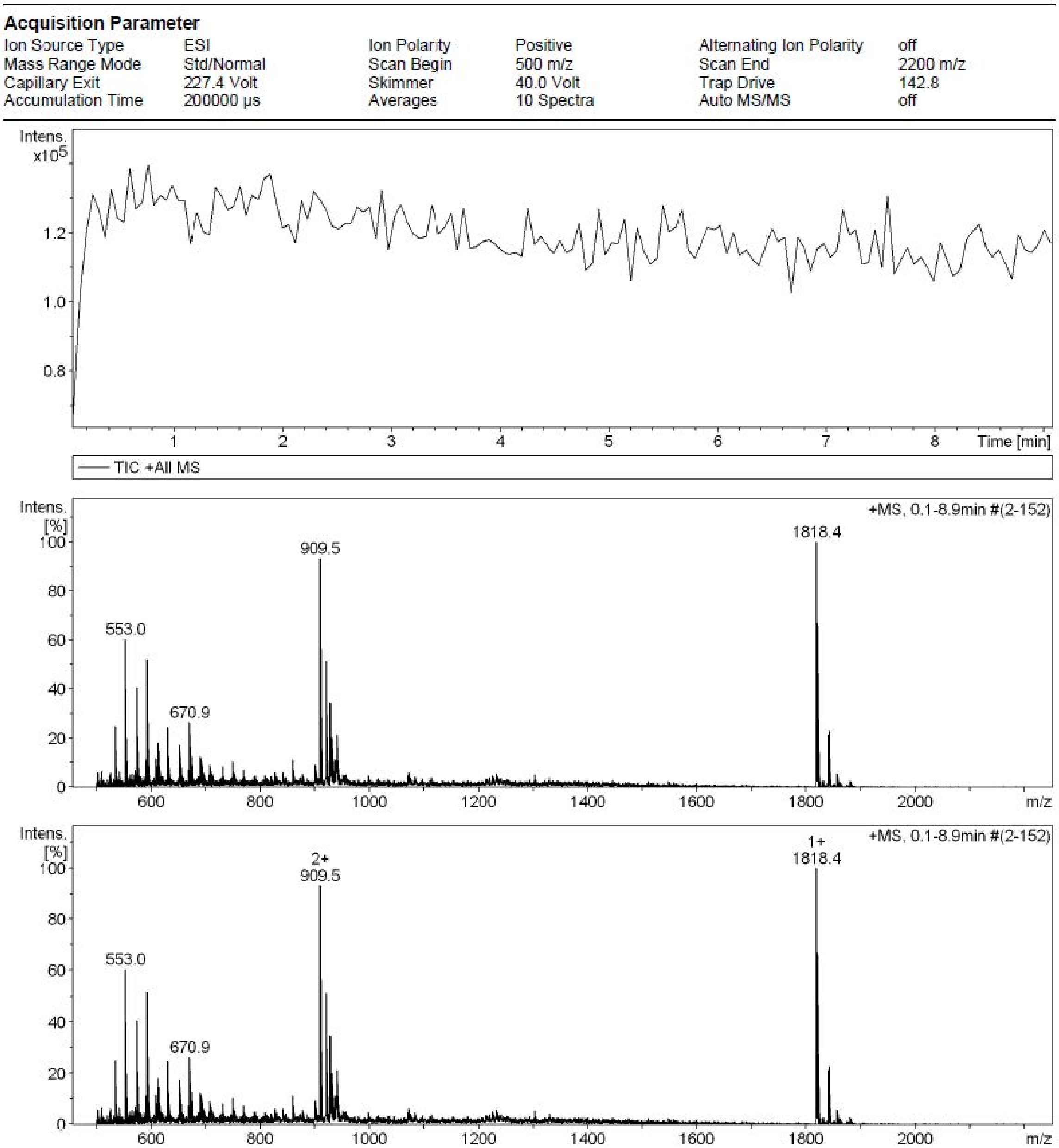

Analytical HPLC:

214 nm

Chromatogram and Results

Injection Details

Injection Name:	**AF488-KP10s 214nm**	*Run Time (min):*	**24,00**
Vial Number:	**RB1**	*Injection Volume:*	**10,00**
Injection Type:	**Unknown**	*Channel:*	**UV_VIS_1**
Calibration Level:		*Wavelength:*	**214**
Instrument Method:	**5_95 in 17min 0,8ml 214nm Auto**	*Bandwidth:*	**n.a.**
Processing Method:	**Basic Quantitative**	*Dilution Factor:*	**1,0000**
Injection Date/Time:	**25.Jul.25 17:25**	*Sample Weight:*	**1,0000**

Chromatogram

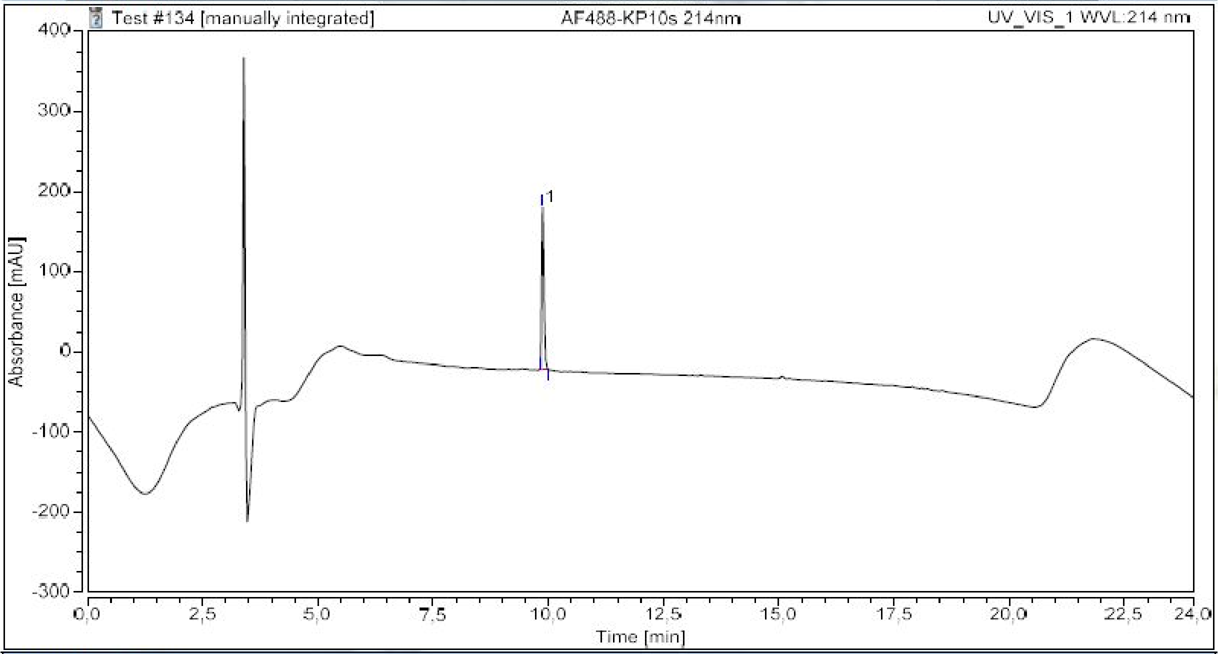

Integration Results

No.	Peak Name	Retention Time min	Area mAU*min	Height mAU	Relative Area %	Relative Height %	Amount n.a.
1	n.a.	9,878	9,534	202,410	100,00	100,00	n.a.
**Total:**			**9,534**	**202,410**	**100,00**	**100,00**	

254 nm

Chromatogram and Results

Injection Details

Injection Name:	**AF488-KP10s 254nm**	*Run Time (min):*	**20,00**
Vial Number:	**RB2**	*Injection Volume:*	**10,00**
Injection Type:	**Unknown**	*Channel:*	**UV_VIS_1**
Calibration Level:		*Wavelength:*	**254**
Instrument Method:	**5_95 in 17min 0,8ml 254nm Auto**	*Bandwidth:*	**n.a.**
Processing Method:	**Basic Quantitative**	*Dilution Factor:*	**1,0000**
Injection Date/Time:	**25.Jul.25 17:50**	*Sample Weight:*	**1,0000**

Chromatogram

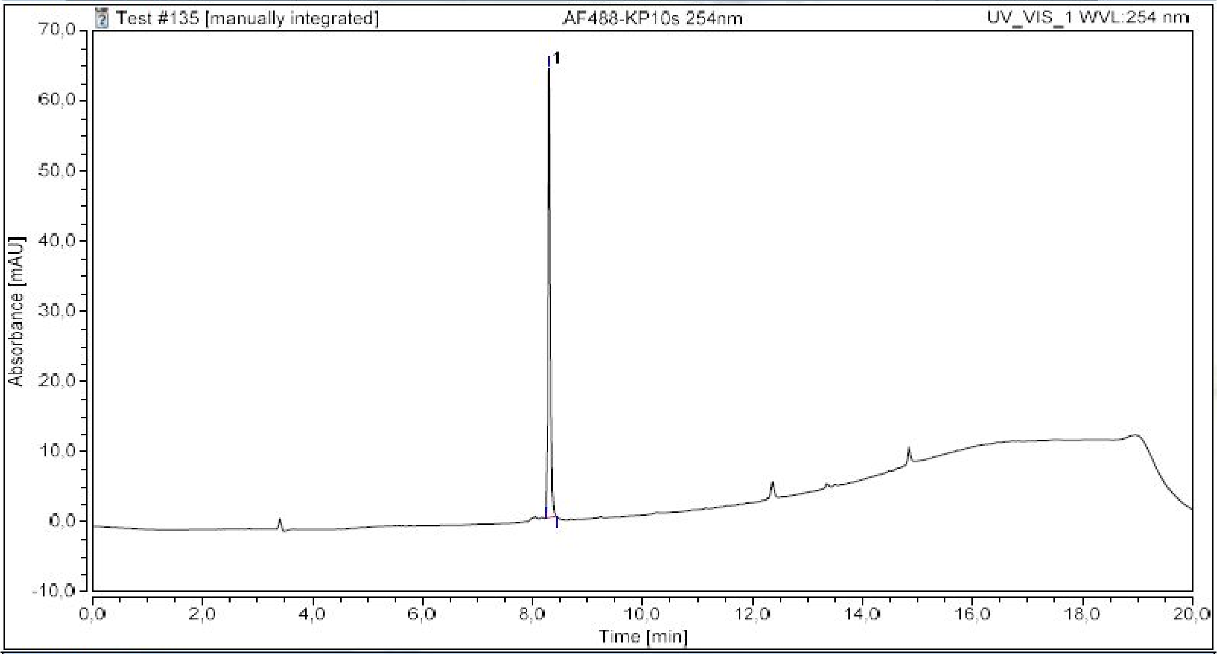

Integration Results

No.	Peak Name	Retention Time min	Area mAU*min	Height mAU	Relative Area %	Relative Height %	Amount n.a.
1	n.a.	8,298	2,854	64,129	100,00	100,00	n.a.
**Total:**			**2,854**	**64,129**	**100,00**	**100,00**	

DOTA-KP-10s

HPLC chromatogram (RP):

(A) Water + 0.1% Trifluoroacetic acid (TFA), (B) ACN + 0.1% TFA; Gradient: 0–40 min 5–60% B; Flow: 30 mL/min; Wavelength: 214 nm; Temperature: RT.

Retention time: 24.56 min

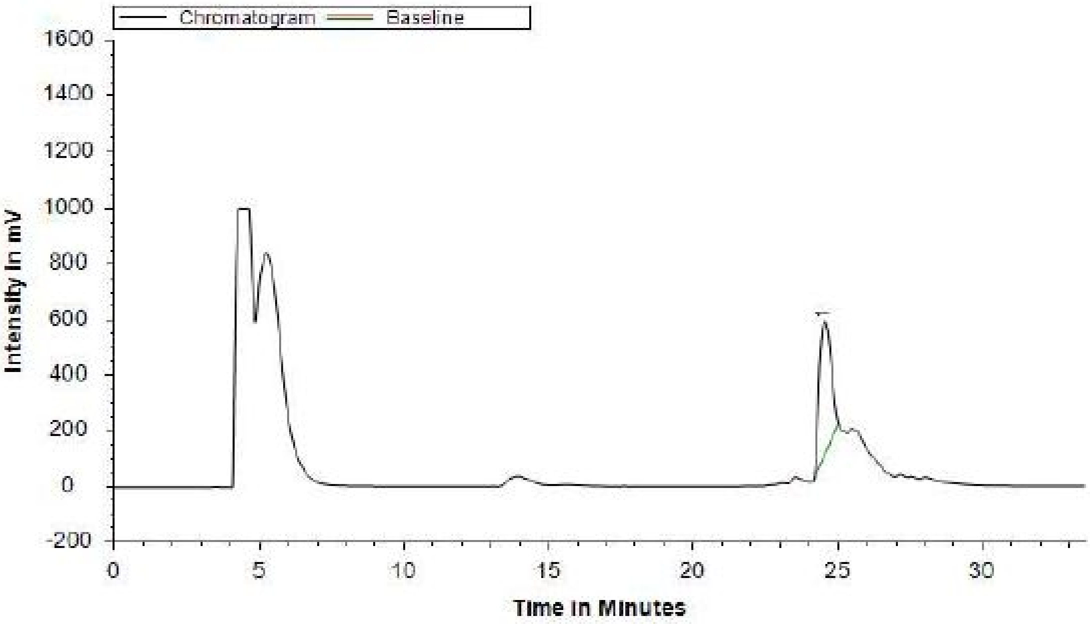

Result Table

No.	Ret. Time	Height	Area	Percent	Name
1	24,55833	476,5956	1,373333E+07	100	

HPLC chromatogram (HILIC):

(A) Water + 0.2% Formic acid (FA), (B) ACN + 0.2% FA; Gradient: 0–40 min 97–50% B; Flow: 15 mL/min; Wavelength: 214 nm; Temperature: RT.

Retention time: 21.48 min

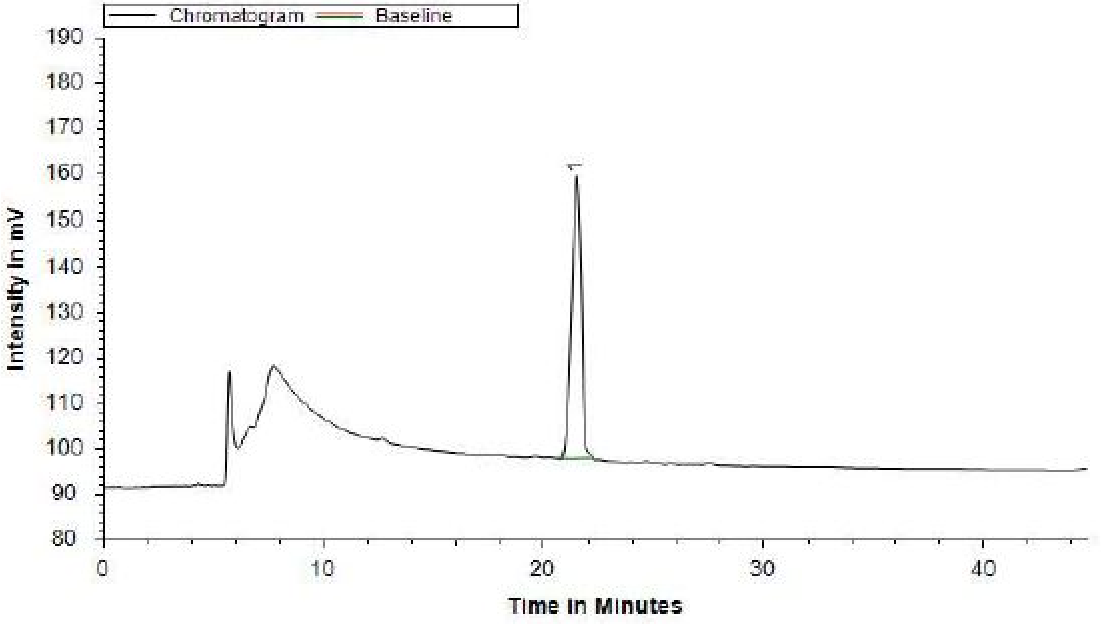

Result Table

No.	Ret. Time	Height	Area	Percent	Name
1	21,475	61,61665	1849691	100	

ESI-MS:

Target mass: 1688.8 g/mol

Detected mass: 1689.5 $[M]^+$, 845.1 $[M/2]^+$

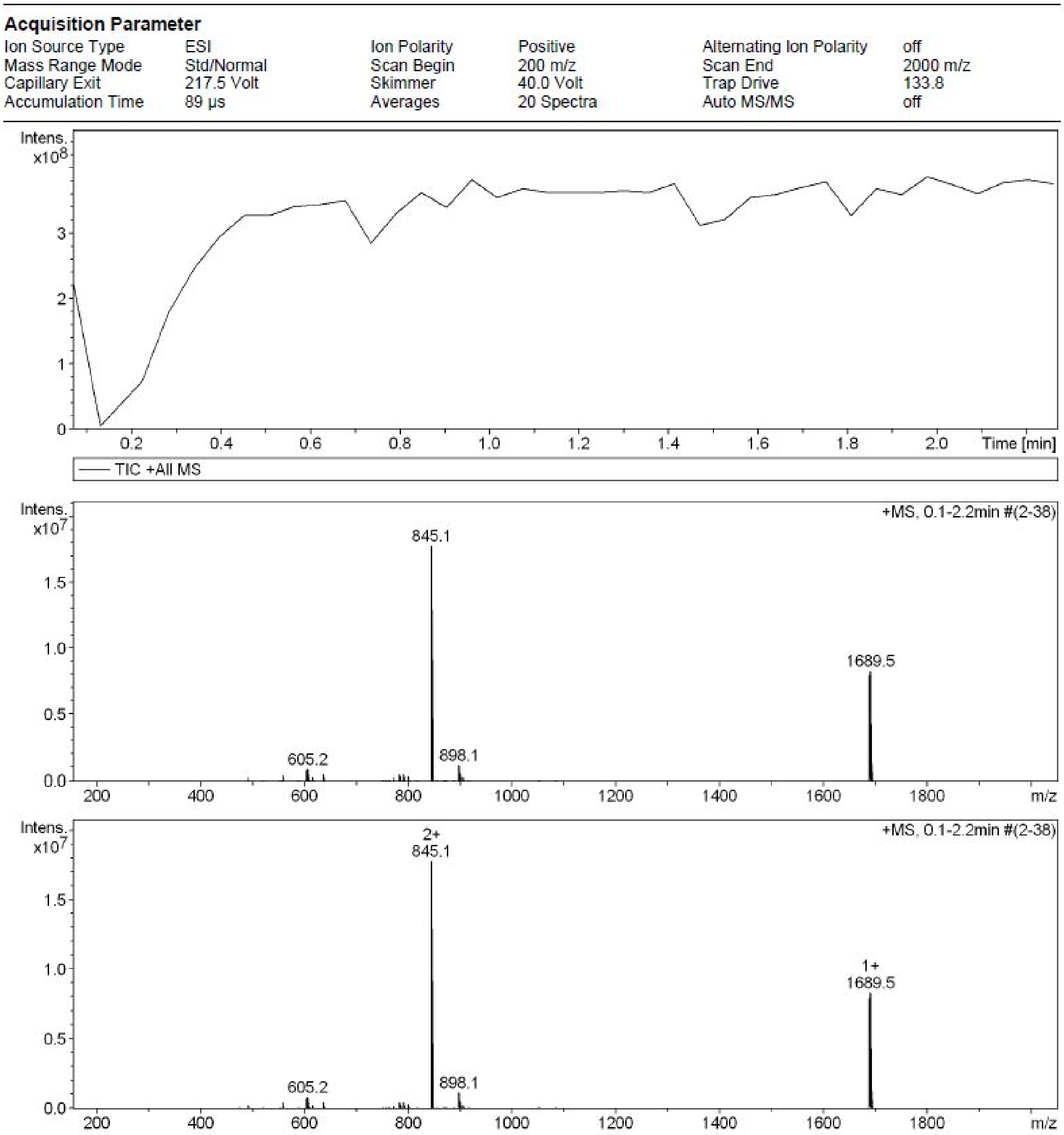

Analytical HPLC:

214 nm

Chromatogram and Results

Injection Details

Injection Name:	**DOTA-KP10s 214nm**	*Run Time (min):*	**24,00**
Vial Number:	**RB1**	*Injection Volume:*	**10,00**
Injection Type:	**Unknown**	*Channel:*	**UV_VIS_1**
Calibration Level:		*Wavelength:*	**214**
Instrument Method:	**5_95 in 17min 0,8ml 214nm Auto**	*Bandwidth:*	**n.a.**
Processing Method:	**Basic Quantitative**	*Dilution Factor:*	**1,0000**
Injection Date/Time:	**25.Jul.25 14:39**	*Sample Weight:*	**1,0000**

Chromatogram

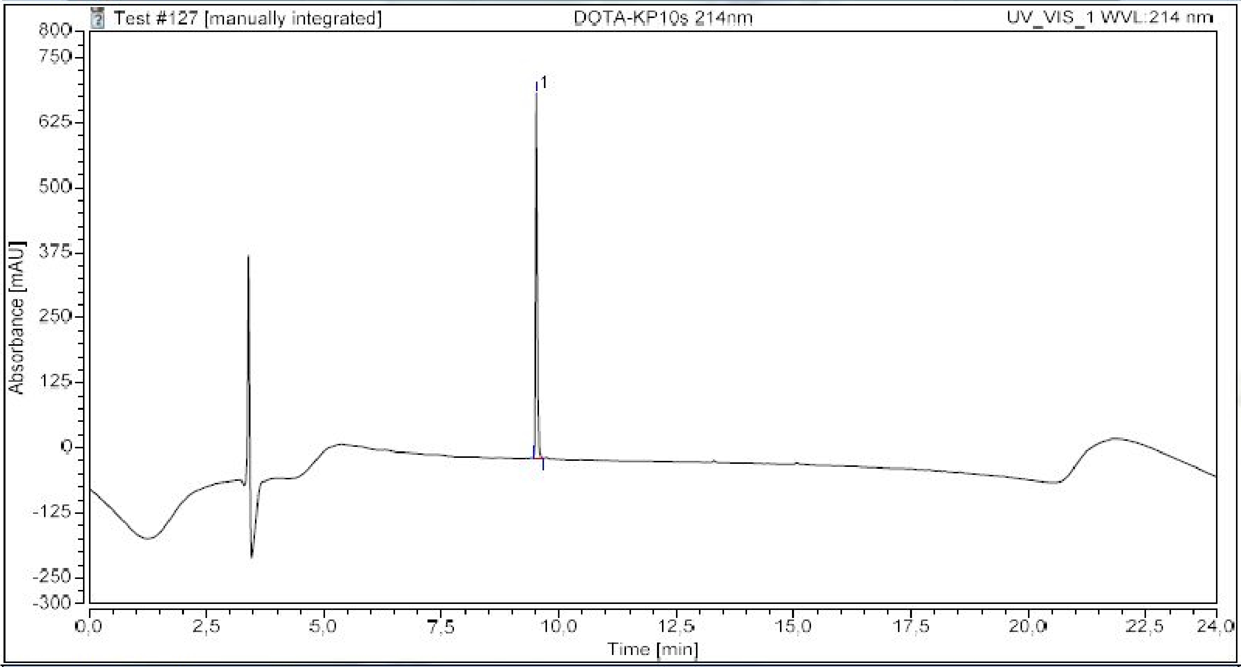

Integration Results

No.	Peak Name	Retention Time min	Area mAU*min	Height mAU	Relative Area %	Relative Height %	Amount n.a.
1	n.a.	9,522	29,077	701,632	100,00	100,00	n.a.
**Total:**			**29,077**	**701,632**	**100,00**	**100,00**	

254 nm

Chromatogram and Results

Injection Details

Injection Name:	**DOTA-KP10s 254nm**	*Run Time (min):*	**20,00**
Vial Number:	**RB2**	*Injection Volume:*	**10,00**
Injection Type:	**Unknown**	*Channel:*	**UV_VIS_1**
Calibration Level:		*Wavelength:*	**254**
Instrument Method:	**5_95 in 17min 0,8ml 254nm Auto**	*Bandwidth:*	**n.a.**
Processing Method:	**Basic Quantitative**	*Dilution Factor:*	**1,0000**
Injection Date/Time:	**25.Jul.25 15:04**	*Sample Weight:*	**1,0000**

Chromatogram

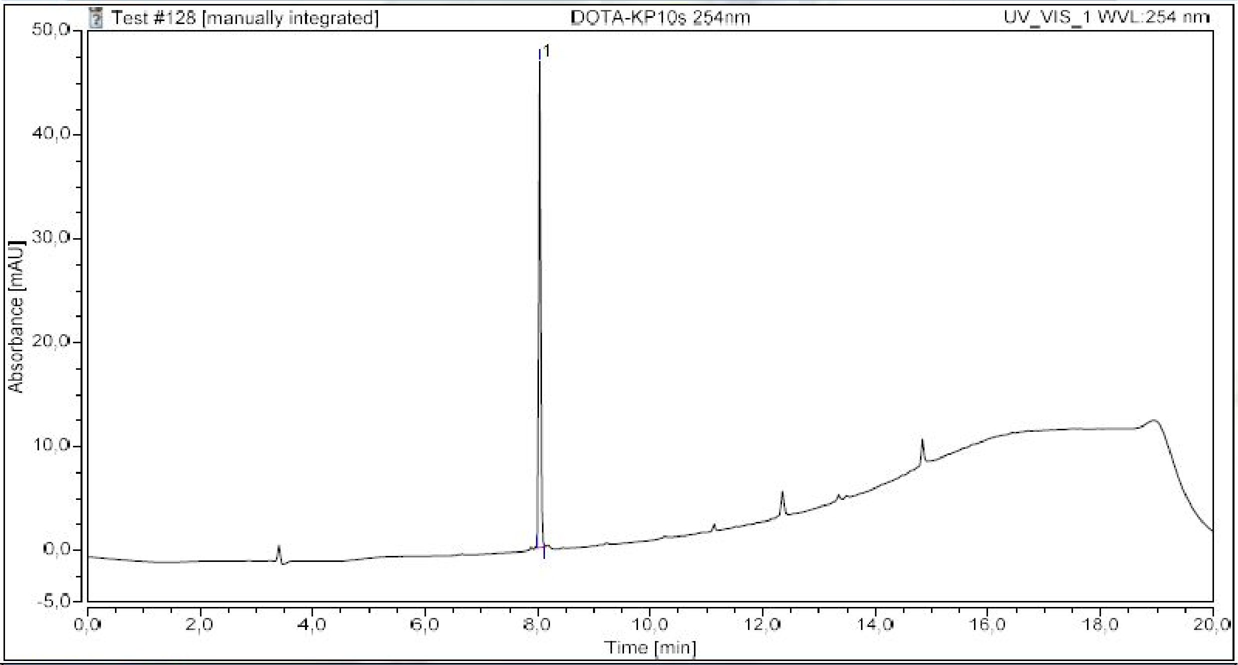

Integration Results

No.	Peak Name	Retention Time min	Area mAU*min	Height mAU	Relative Area %	Relative Height %	Amount n.a.
1	n.a.	8,035	1,813	46,817	100,00	100,00	n.a.
**Total:**			**1,813**	**46,817**	**100,00**	**100,00**	

KiSS-34s

HPLC chromatogram (RP):

(A) Water + 0.1% Trifluoroacetic acid (TFA), (B) ACN + 0.1% TFA; Gradient: 0–40 min 5–60% B; Flow: 30 mL/min; Wavelength: 214 nm; Temperature: RT.

Retention time: 25.85 min

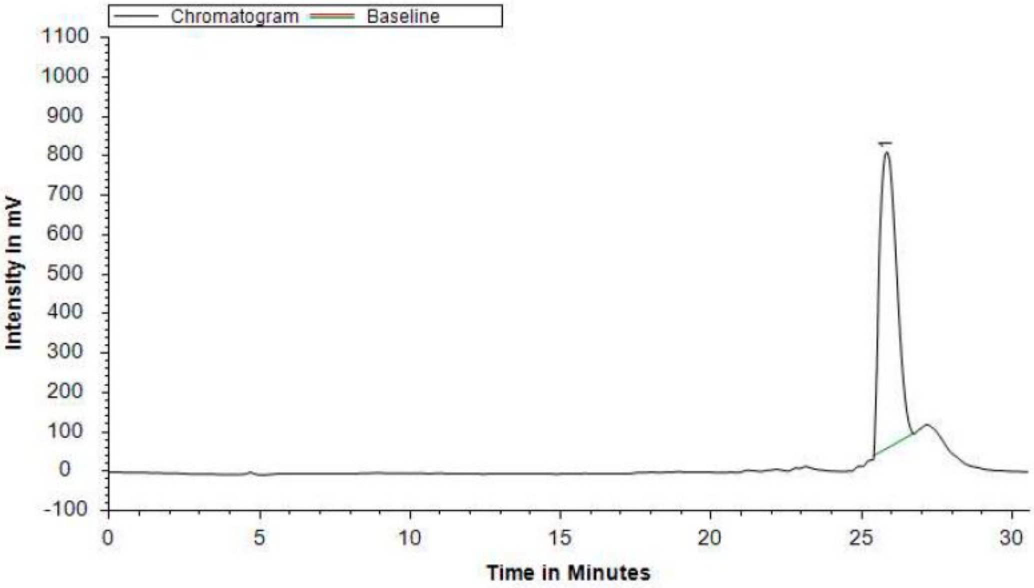

Result Table

No.	Ret. Time	Height	Area	Percent	Name
1	25.85	750.8494	2.979904E+07	100	

ESI-MS:

Target mass: 860.0 g/mol

Detected mass: 860.2 $[M]^+$, 430.6 $[M/2]^+$

Acquisition Parameter

Ion Source Type	ESI	Ion Polarity	Positive	Alternating Ion Polarity	off
Mass Range Mode	Std/Normal	Scan Begin	200 m/z	Scan End	1500 m/z
Capillary Exit	133.2 Volt	Skimmer	40.0 Volt	Trap Drive	57.9
Accumulation Time	422 µs	Averages	10 Spectra	Auto MS/MS	off

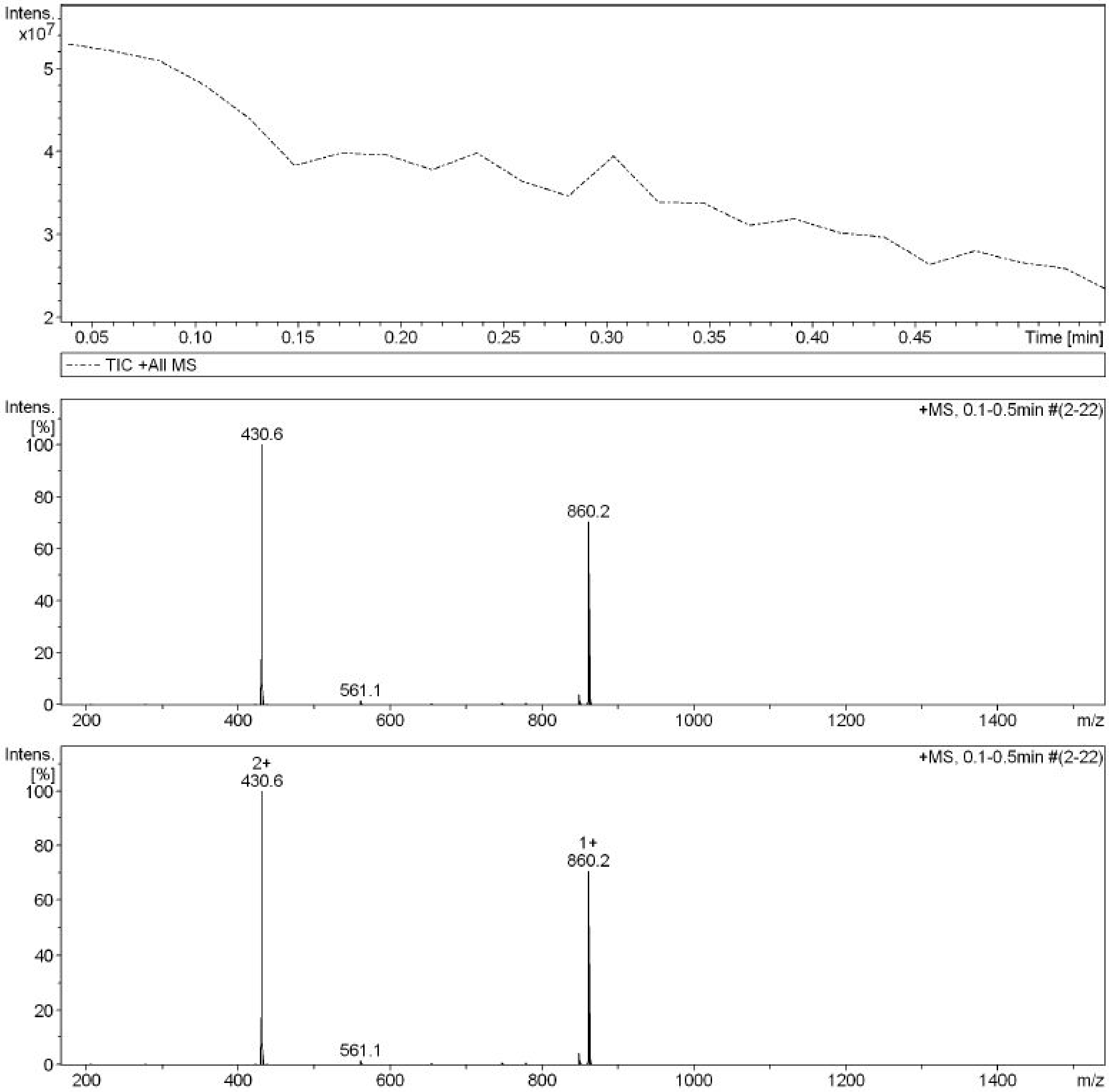

Analytical HPLC:

214 nm

Chromatogram and Results

Injection Details

Injection Name:	**Kp-34s 214nm**	*Run Time (min):*	**24,00**
Vial Number:	**RB1**	*Injection Volume:*	**10,00**
Injection Type:	**Unknown**	*Channel:*	**UV_VIS_1**
Calibration Level:		*Wavelength:*	**214**
Instrument Method:	**5_95 in 17min 0,8ml 214nm Auto**	*Bandwidth:*	**n.a.**
Processing Method:	**Basic Quantitative**	*Dilution Factor:*	**1,0000**
Injection Date/Time:	**30.Jun.25 16:04**	*Sample Weight:*	**1,0000**

Chromatogram

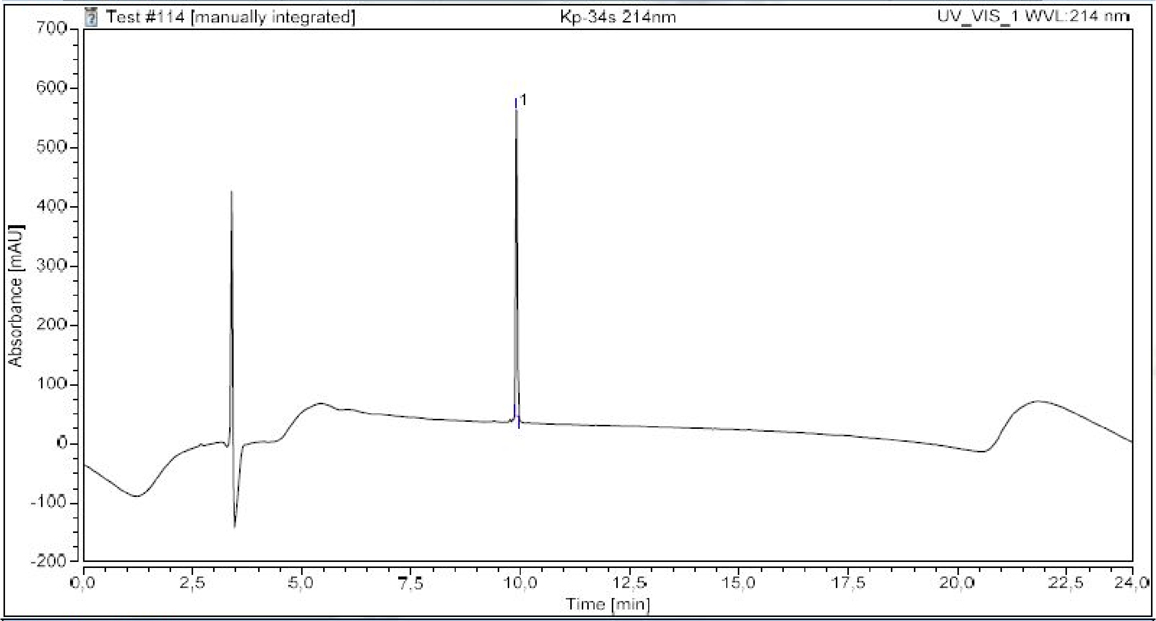

Integration Results

No.	Peak Name	Retention Time min	Area mAU*min	Height mAU	Relative Area %	Relative Height %	Amount n.a.
1	n.a.	9,905	20,716	517,965	100,00	100,00	n.a.
**Total:**			**20,716**	**517,965**	**100,00**	**100,00**	

254 nm

Chromatogram and Results

Injection Details

Injection Name:	**K34s 254nm**	*Run Time (min):*	**20,00**
Vial Number:	**RC1**	*Injection Volume:*	**10,00**
Injection Type:	**Unknown**	*Channel:*	**UV_VIS_1**
Calibration Level:		*Wavelength:*	**254**
Instrument Method:	**5_95 in 17min 0,8ml 254nm Auto**	*Bandwidth:*	**n.a.**
Processing Method:	**Basic Quantitative**	*Dilution Factor:*	**1,0000**
Injection Date/Time:	**30.Jun.25 12:30**	*Sample Weight:*	**1,0000**

Chromatogram

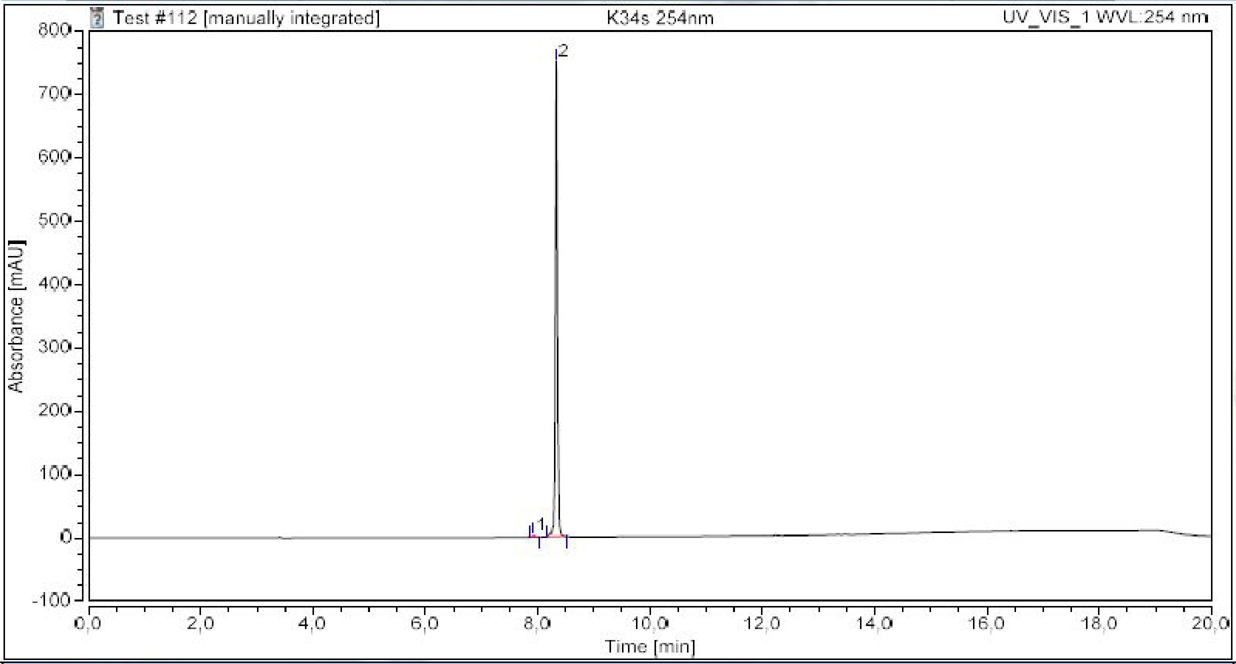

Integration Results

No.	Peak Name	Retention Time min	Area mAU*min	Height mAU	Relative Area %	Relative Height %	Amount n.a.
1	n.a.	7,925	0,098	3,061	0,31	0,41	n.a.
2	n.a.	8,328	31,665	751,272	99,69	99,59	n.a.
**Total:**			**31,763**	**754,332**	**100,00**	**100,00**	

AF-488-KiSS-34s

HPLC chromatogram (HILIC):

(A) Water + 0.2% Formic acid (FA), (B) ACN + 0.2% FA; Gradient: 0–40 min 97–50% B; Flow: 15 mL/min; Wavelength: 214 nm; Temperature: RT.

Retention time: 30.68 min

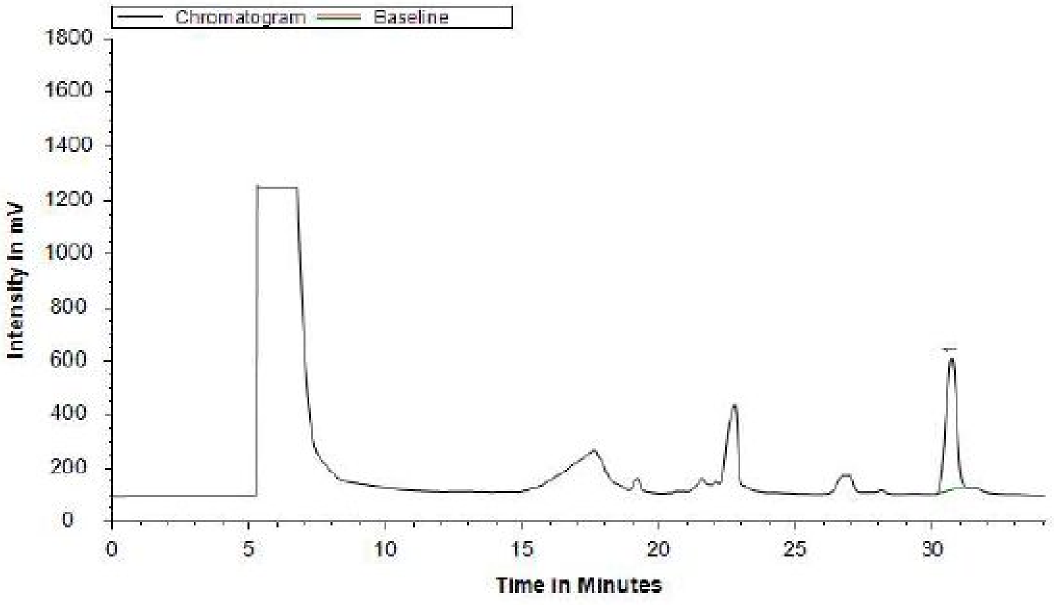

Result Table

No.	Ret. Time	Height	Area	Percent	Name
1	30,68333	492,1078	1,280198E+07	100	

ESI-MS

Target mass: 1376.5 g/mol

Detected mass: 1376.2 $[M]^+$, 688.5 $[M/2]^+$

Acquisition Parameter

Ion Source Type	ESI	Ion Polarity	Positive	Alternating Ion Polarity	off
Mass Range Mode	Std/Normal	Scan Begin	400 m/z	Scan End	1600 m/z
Capillary Exit	215.5 Volt	Skimmer	40.0 Volt	Trap Drive	132.0
Accumulation Time	269 µs	Averages	20 Spectra	Auto MS/MS	off

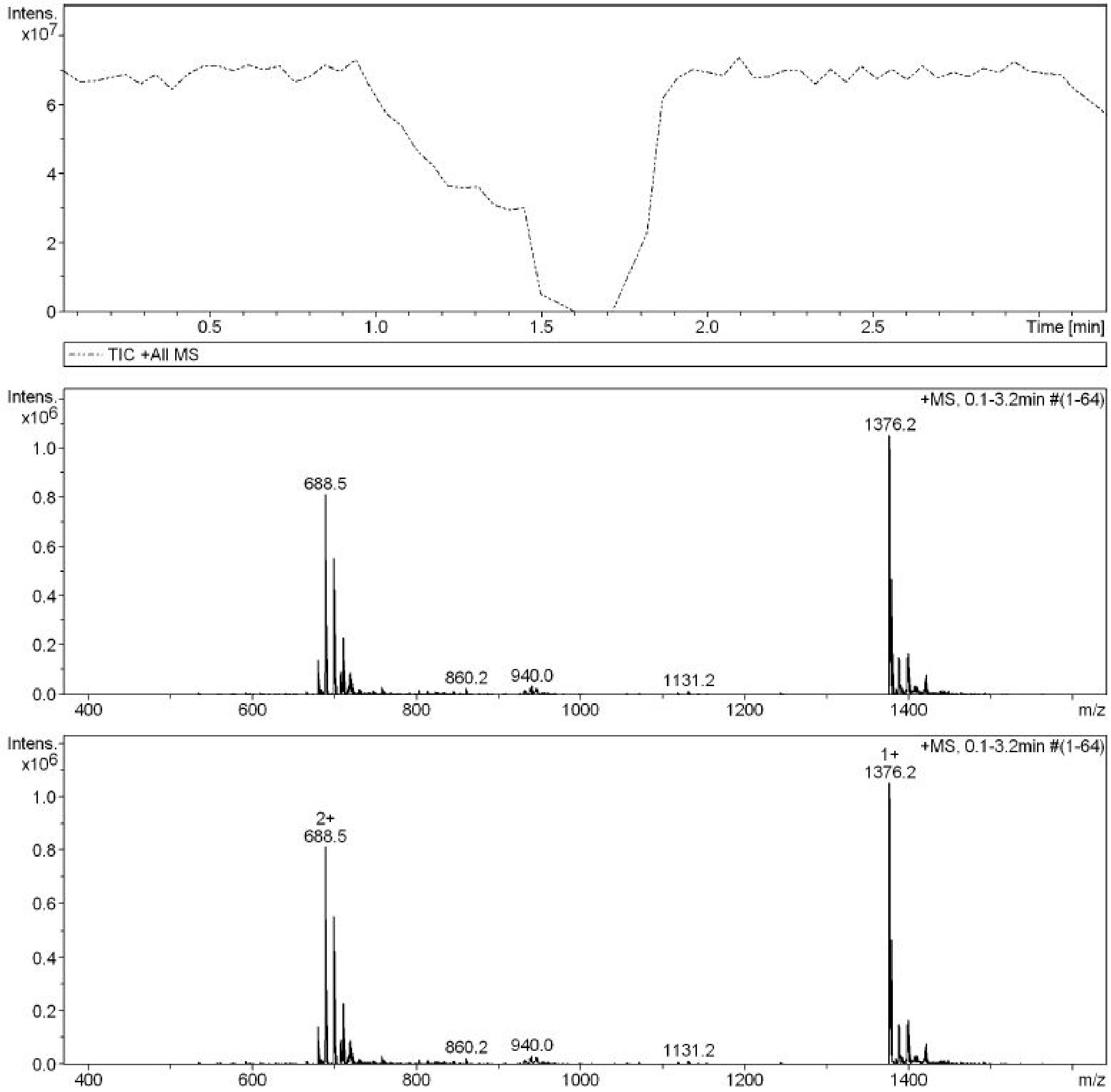

Analytical HPLC:

214, 254, 220 nm

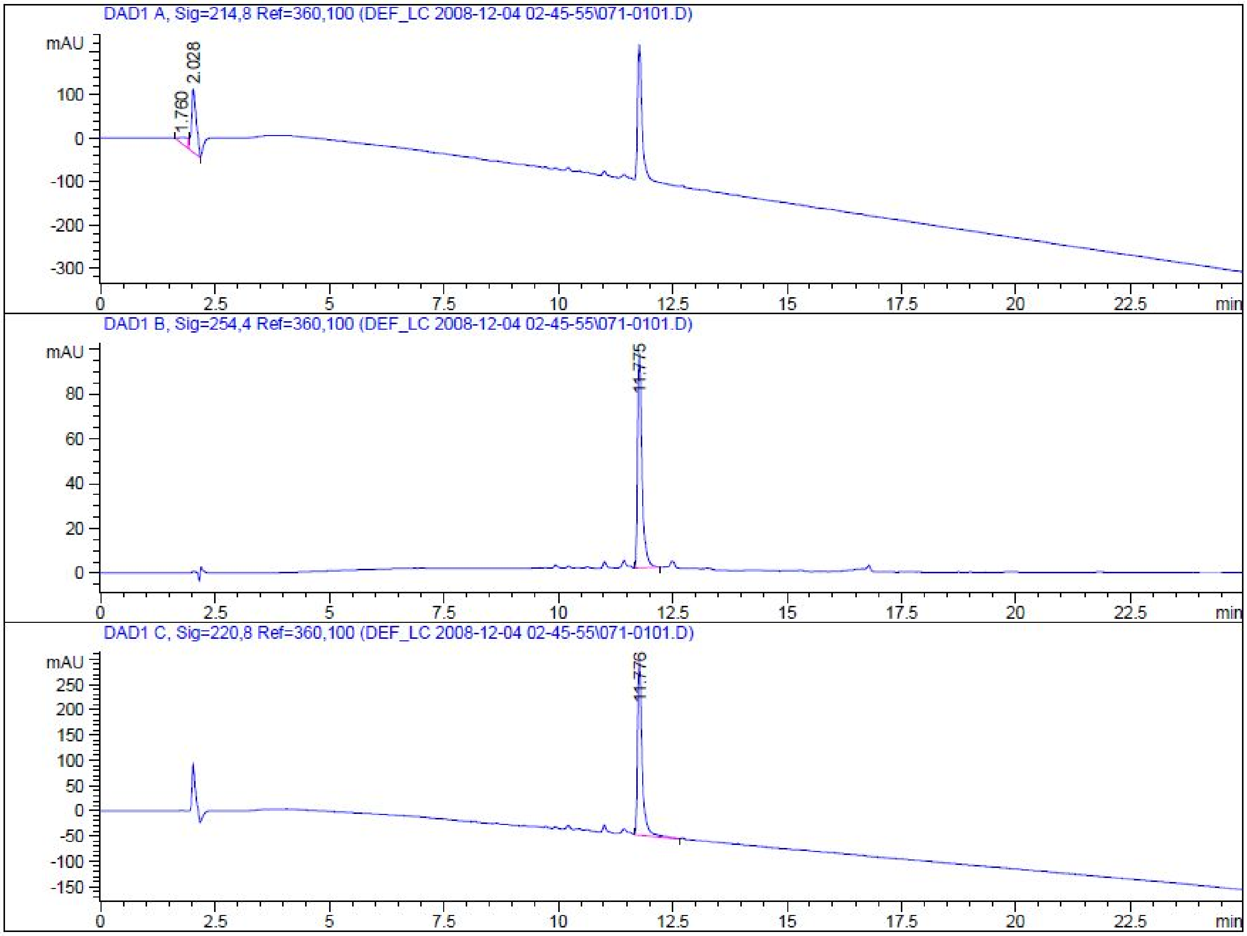

DOTA-KiSS-34s

HPLC chromatogram (RP):

(A) Water + 0.1% Trifluoroacetic acid (TFA), (B) ACN + 0.1% TFA; Gradient: 0–40 min 5–60% B; Flow: 30 mL/min; Wavelength: 214 nm; Temperature: RT.

Retention time: 25.05 min

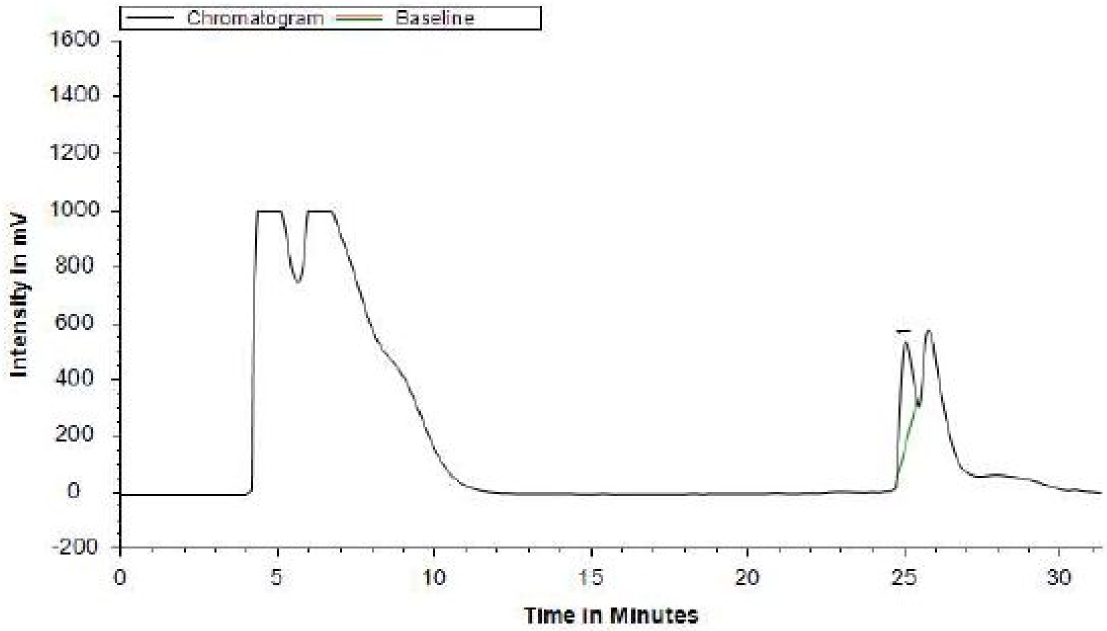

Result Table

No.	Ret. Time	Height	Area	Percent	Name
1	25,05	369,1297	8685247	100	

ESI-MS:

Target mass: 1246.4 g/mol

Detected mass: 1246.4 $[M]^+$, 623.6 $[M/2]^+$

Acquisition Parameter

Ion Source Type	ESI	Ion Polarity	Positive	Alternating Ion Polarity	off		
Mass Range Mode	Std/Normal	Scan Begin	15 m/z	Scan End	1500 m/z		
Capillary Exit	184.4 Volt	Skimmer	40.0 Volt	Trap Drive	104.0		
Accumulation Time	200000 µs	Averages	10 Spectra	Auto MS/MS	off		

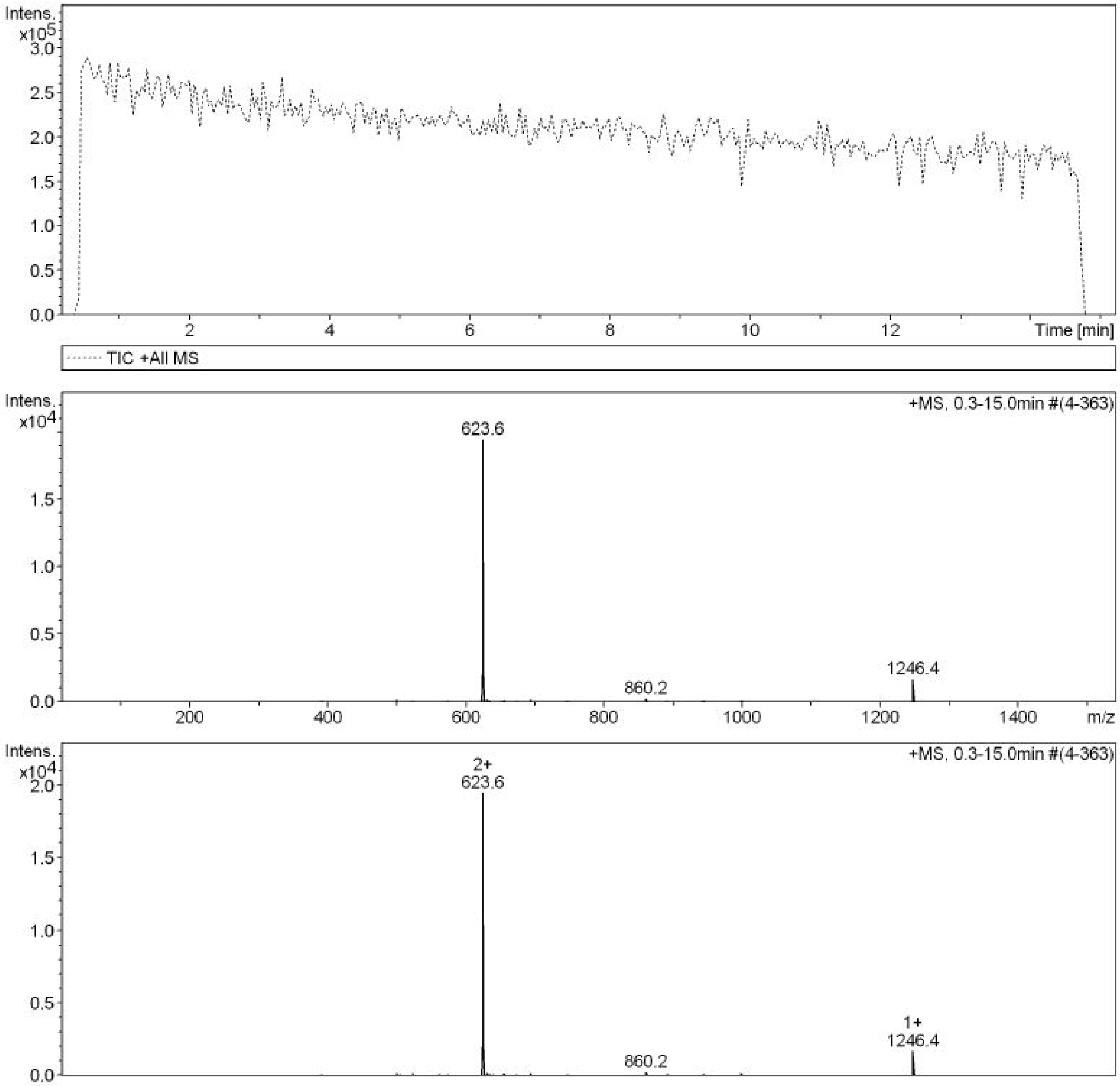

Analytical HPLC:

214 nm

Chromatogram and Results

Injection Details

Injection Name:	**DOTA-KP34s 214nm**	*Run Time (min):*	**24,00**
Vial Number:	**RC1**	*Injection Volume:*	**10,00**
Injection Type:	**Unknown**	*Channel:*	**UV_VIS_1**
Calibration Level:		*Wavelength:*	**214**
Instrument Method:	**5_95 in 17min 0,8ml 214nm Auto**	*Bandwidth:*	**n.a.**
Processing Method:	**Basic Quantitative**	*Dilution Factor:*	**1,0000**
Injection Date/Time:	**25.Jul.25 13:53**	*Sample Weight:*	**1,0000**

Chromatogram

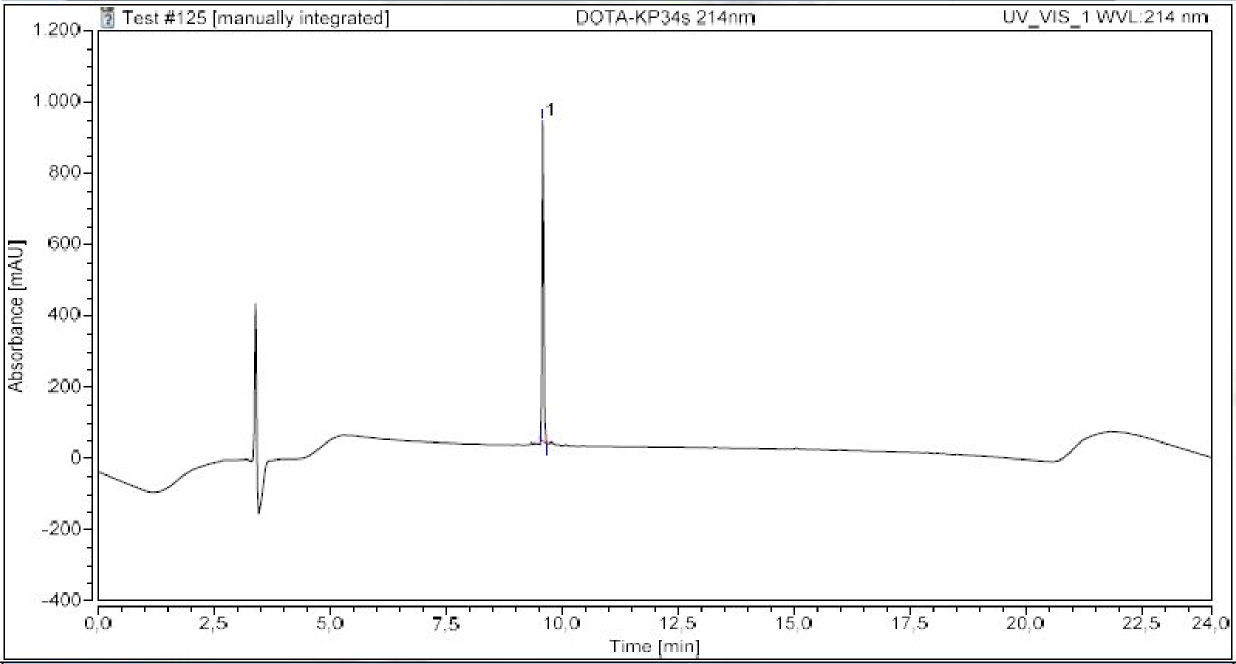

Integration Results

No.	Peak Name	Retention Time min	Area mAU*min	Height mAU	Relative Area %	Relative Height %	Amount n.a.
1	n.a.	9,583	36,317	900,265	100,00	100,00	n.a.
**Total:**			**36,317**	**900,265**	**100,00**	**100,00**	

254 nm

Chromatogram and Results

Injection Details

Injection Name:	**DOTA-KP34s 254nm**	*Run Time (min):*	**20,00**
Vial Number:	**RC2**	*Injection Volume:*	**10,00**
Injection Type:	**Unknown**	*Channel:*	**UV_VIS_1**
Calibration Level:		*Wavelength:*	**254**
Instrument Method:	**5_95 in 17min 0,8ml 254nm Auto**	*Bandwidth:*	**n.a.**
Processing Method:	**Basic Quantitative**	*Dilution Factor:*	**1,0000**
Injection Date/Time:	**25.Jul.25 14:18**	*Sample Weight:*	**1,0000**

Chromatogram

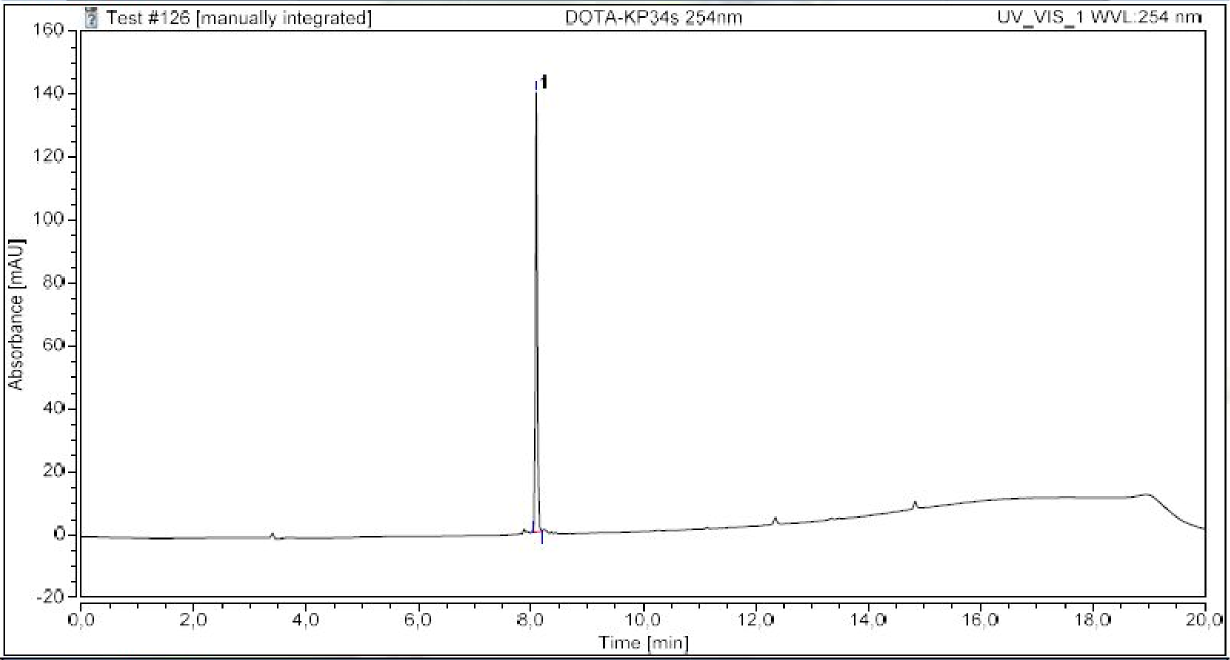

Integration Results

No.	Peak Name	Retention Time min	Area mAU*min	Height mAU	Relative Area %	Relative Height %	Amount n.a.
1	n.a.	8,095	5,450	139,753	100,00	100,00	n.a.
**Total:**			**5,450**	**139,753**	**100,00**	**100,00**	

SI2 – Target Receptor Studies

1. FACS

Adherent cells were washed twice with PBS (without Mg^{2+}/Ca^{2+}; BiochromAG #L1825) and detached with non-enzymatic dissociation buffer (Gibco #13151-014). A detailed list of examined cell lines and corresponding media is available in **Table S5**.

2×10^5 viable cells per well were seeded into 96-well plates. For antibody staining, Abs were added as a master mix with double Ab concentration to compensate the dilution by addition of 25 µL Fc-block (BD Biosciences #564220). Cells were incubated with primary antibodies for 20 minutes (on ice, in the dark, and gentle shaking). Afterwards, cells were washed twice using FACS buffer (800 g, 2 min, 4°C) and incubated with PE-labeled secondary antibodies (10 µg/mL, 30 min, 4°C, in the dark, gentle shaking), washed again and resuspended in 1:1000 diluted Sytox Blue. Isotype controls were conducted to confirm specificity of FACS experiments. A detailed list of primary antibodies, including isotype control, is available in **Table S6**.

For direct staining, cells were incubated with 500 nM of AF-488-KPs for 5 to 60 minutes, depending on the experimental setup, at 4°C. Instead of Fc-block, 25µL of FACS-Buffer (PBS -/- with 3% FCS; Sigma #F2442) was added. A list of applied AF-488-KPs is available in **Table** S7.

Cell lines were gated for living cells first. Afterwards, doublets were discriminated before KISS1R expression investigated (**Figure S1**). In selected cell lines, dead or apoptotic cells were examined instead as evidence suggests upregulation of KISS1R expression in these cells (data not shown). Samples were measured on a BD FACS Canto II (Voltages: FSC: 200 (lin); SSC: 300 (log); FITC (AF-488): 300 (log); PE: 350 (log); Pacific Blue: 350) and analyzed using FlowJo software. All experiments were conducted in triplicates. In all cases, flow cytometry analyses either failed or delivered inconsistent results. This was observed with both commercially available antibodies and AF-488-labeled KPs. Changes to experimental conditions, such as incubation time, temperature, fixation and permeabilization did not improve the binding efficiency and specificity, respectively.

Table S5. List of cell lines with cell culture medium stained for KISS1R expression.

Cell Line	Cell Culture Medium
**CHO-K1 KISS1R**<br>**Chinese Hamster Ovary,**<br>**(Overexpressing reporter cell line)**	DMEM / Hams F12 with Glutamax (Gibco #31331) + 10% FCS Superior (heat inactivated); (SIGMA #S0615) + 0,15% Sodium Bicarbonat (Gibco #25080-060) + 20 mM HEPES (Gibco #15630-056) + 1,35 mM Sodium Pyruvat (Gibco # 11360-70) + Penicillin/Streptomycin (Gibco #15140) + 2 mg/ml G418 Sulfate (Corning #30-243-CI)
**CHO-luc hEP4**<br>**Chinese Hamster Ovary**<br>**(Negative cell line)**	Dulbecco´s MEM/ Ham's F12 (Gibco #31331-028), 10% FCS Superior (heat inactivated); (SIGMA #S0615), 500 µg/ml G418 Sulfate (Corning #30-243-CI)
**MCF-7**<br>**Ductal Breast Carcinoma**	RPMI 1640 with Glutamax (Gibco #61870) + 15% FCS Superior (heat inactivated); (SIGMA #S0615) + 1x Non-Essential Amino Acids (Gibco #11140-035) + 1 mM Sodium Pyruvat (Gibco # 11360-70) + 10 µg/ml human insulin (SIGMA #I9278)
**LNCaP**<br>**Prostate Carcinoma**<br>**(Lymph nodes)**	RPMI 1640 with Glutamax (Gibco #61870) + 20% FCS Superior (heat inactivated); (SIGMA #S0615)
**TALL-1**<br>**T-Cell Leukemia**	RPMI 1640 with Glutamax (Gibco: 61870) + 15% FCS Superior (heat inactivated); (SIGMA #S0615)
**HepG2**<br>**Hepatocellular Liver Carcinoma**	RPMI 1640 with Glutamax (Gibco: 61870) + 10% FCS Superior (heat inactivated); (SIGMA #S0615)
**VCaP**<br>**Prostate Neoplasms**	Dulbecco´s MEM/ Ham's F12 (Gibco #31331-028), 10% FCS Superior (heat inactivated); (SIGMA #S0615)

Table S5 continuation. List of cell lines with cell culture medium stained for KISS1R expression.

Cell Line	Cell Culture Medium
**TT**<br>**Thyroid Neoplasms**	Kaighn's Modification of Ham's F12 (Gibco #21127-022) + 10% FCS Superior (heat inactivated); (SIGMA #S0615)
**NCI-H1781**<br>**Lung Adenocarcinoma**	RPMI 1640 with Glutamax (Gibco: 61870) + 10% FCS Superior (heat inactivated); (SIGMA #S0615)
**NUGC-4**<br>**Signet Ring Cell Stomach Carcinoma**	RPMI 1640 with Glutamax (Gibco: 61870) + 10% FCS Superior (heat inactivated); (SIGMA #S0615)
**RPMI-8226**<br>**Multiple Myeloma**	RPMI 1640 with Glutamax (Gibco: 61870) + 10% FCS Superior (heat inactivated); (SIGMA #S0615)
**T-47D**<br>**Ductal Breast Neoplasms**	RPMI 1640 with Glutamax (Gibco #61870) + 15% FCS Superior (heat inactivated); (SIGMA #S0615) + 10 µg/ml human insulin (SIGMA #I9278)
**MDA-MB-231**<br>**Breast Adenocarcinoma**	Dulbecco´s MEM/ Ham's F12 (Gibco #31331-028), 10% FCS Superior (heat inactivated); (SIGMA #S0615)
**MDA-MB-435**<br>**Melanoma**	RPMI 1640 with Glutamax (Gibco: 61870) + 10% FCS Superior (heat inactivated); (SIGMA #S0615)

Table S6. List of commercial antibodies used in KISS1R detection experiments.

Antibody	Company (Cat.No)	Tested Concentration
**rabbit IgG1 Isotype Control**	Thermo Scientific (#PA5-23094)	40 µg/mL
**KISS1R-Specific Rabbit Polyclonal antibody**	Proteintech (#15505-1-AP)	2.5 µg/mL
**anti-KISS1R (GPR54), Rabbit**	alomone labs (#AKR-001)	40 µg/mL
**AffinePure F(ab')$_2$ Fragment Goat Anti-Rabbit IgG (H+L), PE-labeled**	Jackson Immuno Research (#111-116-144)	10 µg/mL

Table S7. List of AF-488-KPs and unlabeled KPs used for KISS1R detection.

Peptide	Usage	Tested Concentration
**AF-488-KP-10**	Detection of KISS1R	500 nM
**AF-488-KP-54**	Detection of KISS1R	500 nM
**KP-54**	Block for AF-488-KP-10, and AF-488-KP-54	10 µM

Gating strategy

All cell lines have been gated for living cells first. Afterwards doublets were discriminated before KISS1R expression was examined (**Figure S1**).

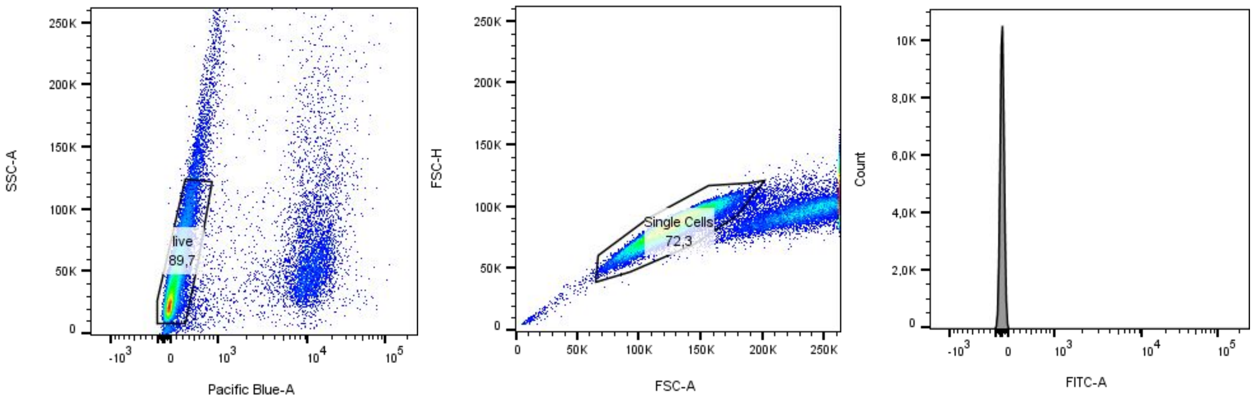

Figure S1. Exemplary presentation of gating strategy in FlowJo.

2. IHC

IHC staining with Abs were conducted following the Nuvisan best-practice protocol. Abs titration was performed on FFPE cell pellets and humane tissue. For this purpose, placenta samples of high KISS1R expression (52.8 tpm) was used with low-expressing colon tissue (9 tpm) as background. Applied Abs and corresponding results are described in **Table S9**.

Ligand-based stainings were conducted using AF-488-KPs on RPMI-8226 and TALL-1 cryo sections (5µm). Cryo sections were fixed with ice-cold aceton for 5 min at –20°C and dried at RT for 30 min. Followed by rehydrated in washing buffer (1 x PBS, 0.05% Tween-80), blocked for 60 min at RT using 5% BSA in washing buffer. AF-488-based ligands were incubated at 10 and 100 µM in blocking buffer and incubated on the cryo sections for 90 min at RT in the dark. Afterwards, cryo sections were washed with 2 x 2.5 min in washing buffer, 1 x 1 min in PBS, followed by 1 x 1 min bidistilled water. Finally, slides were covered using a vectashield with DAPI and were dried overnight, before being scanned using a panoramic scanner. Results and experimental conditions are listed in **Table S10**.

Table S8. List of cell material used in KISS1R IHC experiments.

	Description	KISS1R Expression Level (CCLE)	Indication
**Cell pellets**	NCI-H1048	5.60	Non-Small Cell Lung Cancer
	CAL-51	3.33	Breast Cancer
	HCT15	2.22	Colorectal Cancer
	HT29	0.08	Colorectal Cancer
	A549	0.01	Non-Small Cell Lung Cancer
	MDA-MB-453	0.13	Breast Cancer

	Description	KISS1R Expression Level (tpm)	Comment
**Human tissue**	Placenta	52.8	High expression
	Colon	9	Background Control

	Description		Comment
**Cryo sections**	RPMI8226	KISS1R positive	5 µm cryo sections
	TALL1	KISS1R negative	Control cell line

Table S9. List of commercial antibodies used in KISS1R IHC experiments.

Antibody	Tested Concentration	Results
**KISS1R Antibody (rabbit, polyclonal)** Thermo Scientific (#PA596221)	**Cell pellets:** 1:100 (5,2 µg/mL) 1:300 (1,7 µg/mL) 1:1000 (0,52 µg/mL)	Non-specific staining of negative controls at 5 µg/mL, no staining at lower concentrations
**Anti-KISS1R antibody (rabbit, polyclonal)** Merck (#HPA071913)	**Cell pellets:** 1:30 (1,7 µg/mL) 1:100 (0,5 µg/mL)	Non-specific staining
**KiSS1R/GPR54 Antibody, BSA Free (rabbit, polyclonal)** Novusbio (#NLS1927)	**Cell pellets:** 1:200 (5 µg/mL) 1:600 (1,7 µg/mL) 1:2000 (0,5 µg/mL)	Non-specific staining of negative controls at 5 µg/mL, no staining at lower concentrations
**Anti-KISS1R Ab (rabbit polyclonal, C-terminal)** Abcam (#ab188995) C-terminal	**Cell pellets:** 1:200 (5 µg/mL) 1:600 (1,7 µg/mL) 1:2000 (0,5 µg/mL)	High non-specific staining
	**hTissue:** 1:2000, 1:4000	High non-specific staining
**KISS1R (D9D7C) (rabbit, monoclonal Ab)** Cell Signaling Technology (#13776, clone D9D7C, C-terminal)	**Cell pellets:** 1:10 (2,8 µg/mL) 1:100 (0,28 µg/mL) 1:300 (0,09 µg/mL) 1:1000 (28 ng/mL)	No staining

Table S10. List of AF-488-KPs tested on RPMI8226 and TALL1 cryo sections for IHC studies.

Peptide	Tested Concentration	Results
**AF-488-KP-10**	10, 100 μM	Staining of RPMI8226 and TALL1 (KISS1R-negative) at high concentrations (100 μM),<br>FITC signals too weak at high peptide concentrations of 10 μM
**AF-488-KP-54**		Very few FITC signals at 100 μM only,<br>No signals at 10 μM,<br>Compared to KP-10, not suitable for IHC
**AF-488-KP-10-EEE**		Staining of RPMI8226 and TALL1 (KISS1R-negative) at high concentrations (100 μM)

3. Proteomics / MS

Table S11. KISS1R-specific peptide sequences used for KISS1R detection via Proteomics/MS.

Sequence	Charge	Rt [min]	Windows [min]
AYCSEAFPSR	2	11	10
VAVRPAPADSALQGQVLAER	3	20	10
WYVTVFPLR	2	39	10
LGSHPAPAR	2	25	10

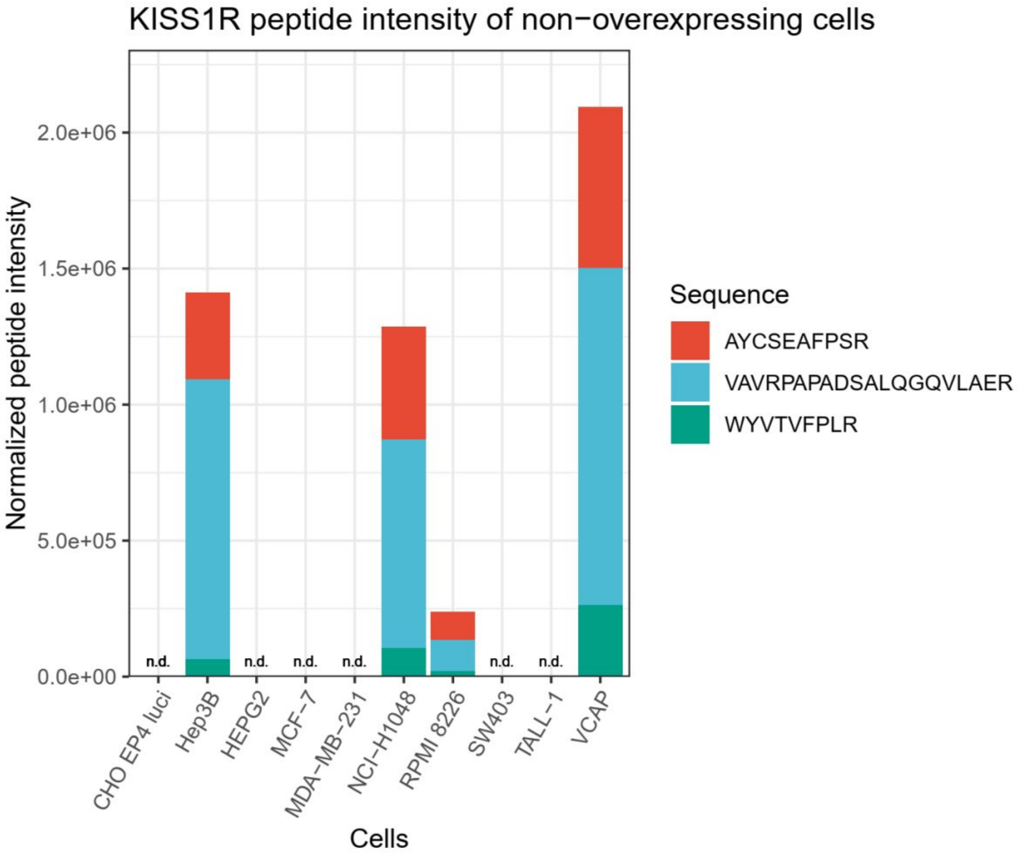

Figure S2. Detection of KISS1R-specific peptide sequences in KISS1R-positive cell lines.

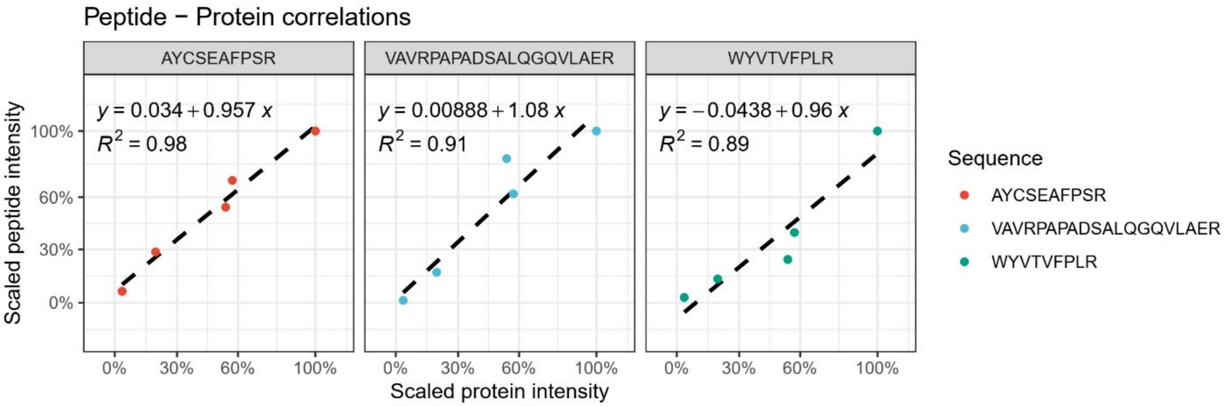

Figure S3. Peptide-protein correlations of KISS1R-specific peptide sequences.

4. Live-cell imaging

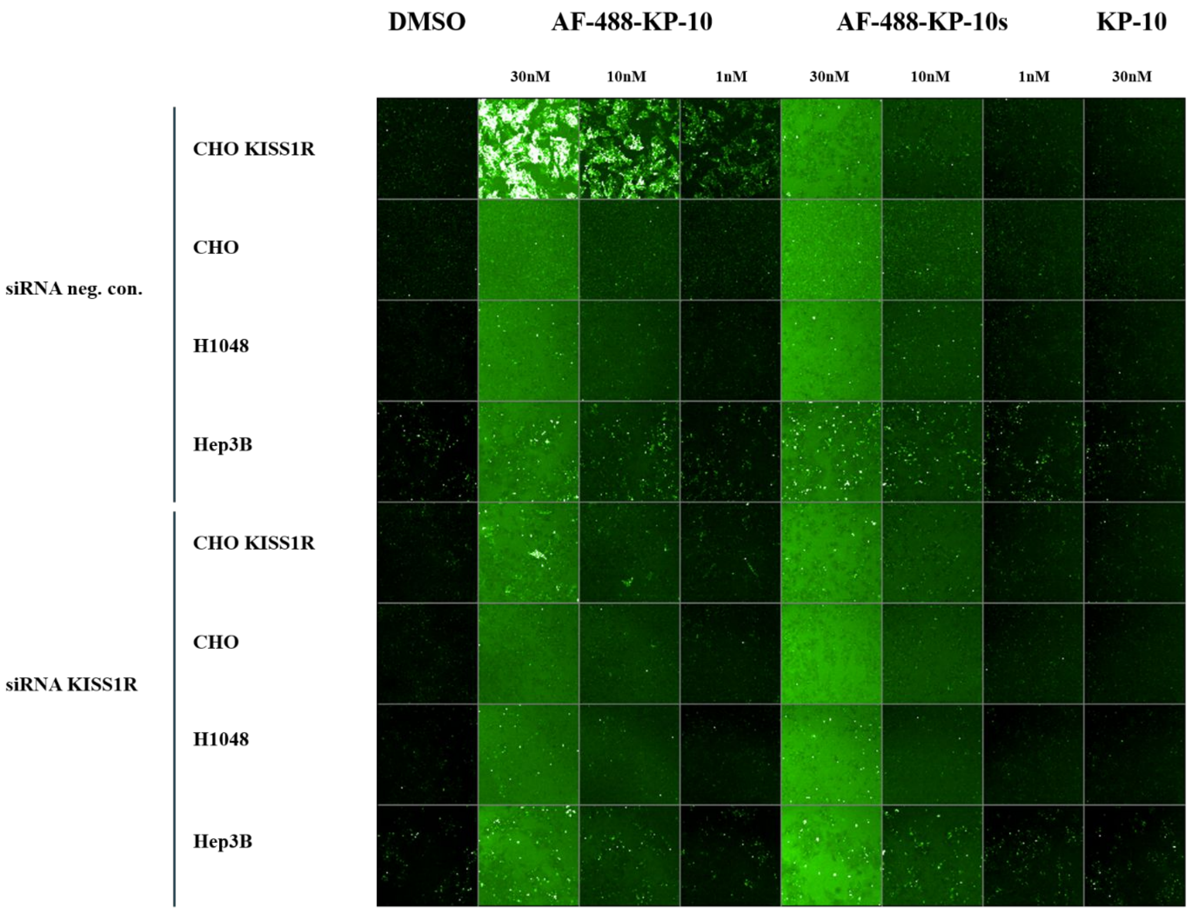

Figure S4. Binding and internalization studies in CHO-KISS1R, CHO-WT without KISS1R expression, and native cancer cells (H1048, Hep3B) following siRNA-mediated knockdown of KISS1R. AF-488-KP-10 and AF-488-KP-10s were incubated with different concentrations (30, 10, 1 nM) for 1 hour at 37°C and 5% CO_2. Used objective: 20x water objective, confocal.

SI3 – Internalization Assays

Concentration-based Cellular Uptake

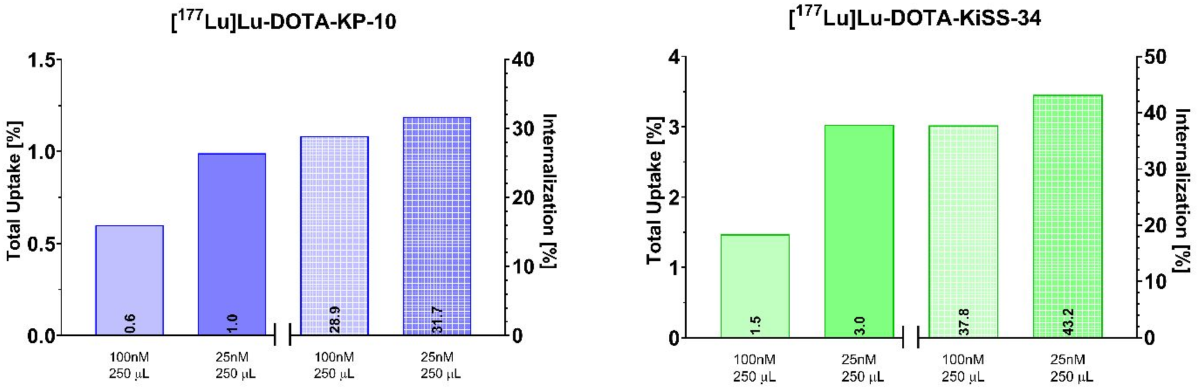

Figure S5. Impact of radioligand concentration (25, 100 nM) on total binding and internalization rates. Cells were incubated for 1 h at 37°C, used cell medium volume = 250 µL.

Concentration- and Volume-based Cellular Uptake

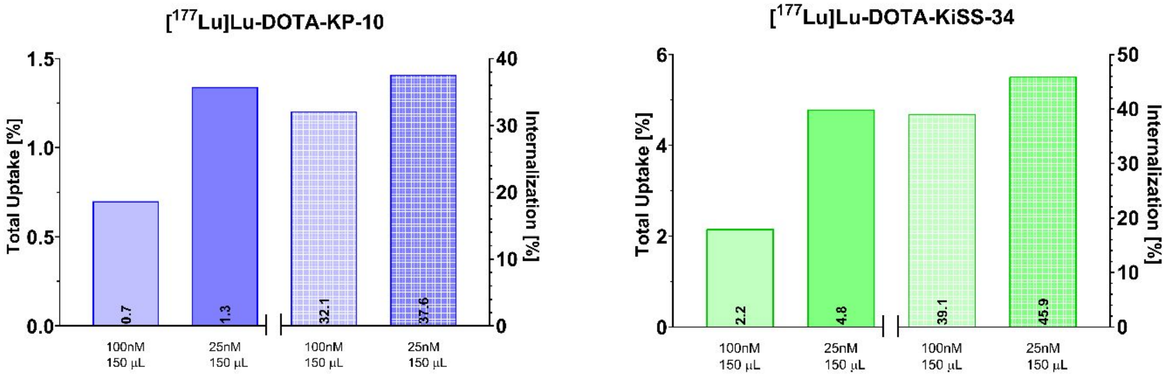

Figure S6. Impact of radioligand concentration (25, 100 nM) and cell medium volume (150 µL) on total binding and internalization rates. Cells were incubated for 1 h at 37°C.

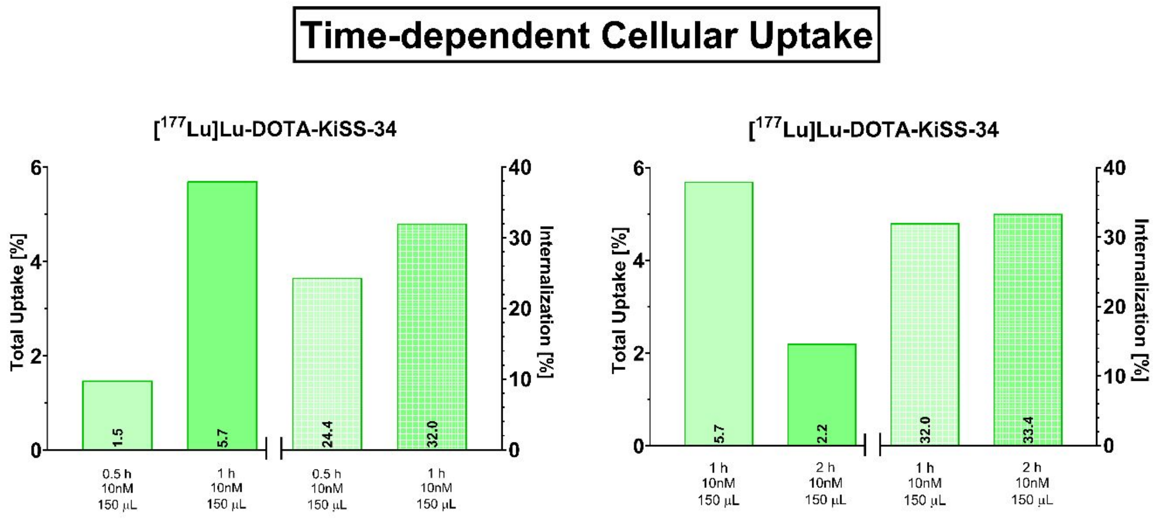

Figure S7. Impact of incubation time (0.5, 1, 2 h) on total binding and internalization rates. Cells were incubated at 37°C with 10 nM [^{177}Lu]Lu-DOTA-KiSS-34 in 150 µL Opti-MEM.

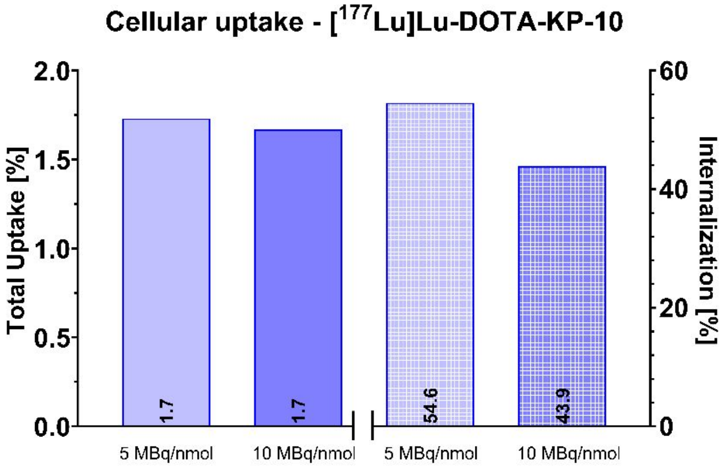

Figure S8. Impact comparison of different applied specific activities on the general cellular uptake with [^{177}Lu]Lu-DOTA-KP-10. Cells were incubated for 1 h at 37°C, used cell medium volume = 150 µL.

SI4 – Oil-based binding kinetics assay

Table S12. Total uptake kinetics with various [^{177}Lu]Lu-DOTA-KPs in KISS1R-negative SKOV3 cells.

Time [min]	Total uptake [%]	
	**[^{177}Lu]Lu-DOTA-KP-10**	**[^{177}Lu]Lu-DOTA-KiSS-34**
0	0.1	0.2
5	0.5	0.5
15	0.5	0.1
30	0.1	0.1
60	0.2	0.1

5×10^6 cells; conducted in a mixture of silicon-mineral oil (4:1 ratio), c ([^{177}Lu]Lu-DOTA-KP) = 10 nM.
